# Extravertebral low back pain: a scoping review

**DOI:** 10.1186/s12891-024-07435-9

**Published:** 2024-05-07

**Authors:** Anna Kunow, Julia Freyer Martins Pereira, Jean-François Chenot

**Affiliations:** Department of General Practice, University Medicine Greifswald, 17475 Fleischmannstraße, Greifswald, Germany

**Keywords:** Extravertebral, Non-vertebral, Non-spinal, Low back pain

## Abstract

**Background:**

Low back pain (LBP) is one of the most common reasons for consultation in general practice. Currently, LBP is categorised into specific and non-specific causes. However, extravertebral causes, such as abdominal aortic aneurysm or pancreatitis, are not being considered.

**Methods:**

A systematic literature search was performed across MEDLINE, Embase, and the Cochrane library, complemented by a handsearch. Studies conducted between 1 January 2001 and 31 December 2020, where LBP was the main symptom, were included.

**Results:**

The literature search identified 6040 studies, from which duplicates were removed, leaving 4105 studies for title and abstract screening. Subsequently, 265 publications were selected for inclusion, with an additional 197 publications identified through the handsearch. The majority of the studies were case reports and case series, predominantly originating from specialised care settings. A clear distinction between vertebral or rare causes of LBP was not always possible. A range of diseases were identified as potential extravertebral causes of LBP, encompassing gynaecological, urological, vascular, systemic, and gastrointestinal diseases. Notably, guidelines exhibited inconsistencies in addressing extravertebral causes.

**Discussion:**

Prior to this review, there has been no systematic investigation into extravertebral causes of LBP. Although these causes are rare, the absence of robust and reliable epidemiological data hinders a comprehensive understanding, as well as the lack of standardised protocols, which contributes to a lack of accurate description of indicative symptoms. While there are certain disease-specific characteristics, such as non-mechanical or cyclical LBP, and atypical accompanying symptoms like fever, abdominal pain, or leg swelling, that may suggest extravertebral causes, it is important to recognise that these features are not universally present in every patient.

**Conclusion:**

The differential diagnosis of extravertebral LBP is extensive with relatively low prevalence rates dependent on the clinical setting. Clinicians should maintain a high index of suspicion for extravertebral aetiologies, especially in patients presenting with atypical accompanying symptoms.

## Background

Fundamentally, low back pain (LBP) represents a symptom rather than an aetiological diagnosis *per se*. Since establishing a definite pathophysiological diagnosis is often neither necessary nor possible, most clinical guidelines pragmatically distinguish between non-specific LBP, specific LBP, and sciatica/radiculopathy [1]. Furthermore, extravertebral or non-spinal medical disorders may mimic or present clinically as LBP. Consequently, some guidelines recommend considering extravertebral or non-spinal diseases in the differential diagnosis. Recognising extravertebral causes is crucial to avoid misdiagnosis and inappropriate management of potentially life-threatening diseases. In settings where patients have direct access to specialised care, such as orthopaedics and physiotherapy, the likelihood of considering non-musculoskeletal disease may be lower [2]. Deyo and Weinstein once estimated that approximately 2% of patients presenting with LBP in primary care have what they referred to as “visceral” disease. However, this percentage lacks a specific data source [3], yet it has been consistently cited in subsequent literature [4–16]. Within a specialist setting, it has been estimated that up to 10-25% of patients presenting with back pain do not have a vertebral pathology [17].

**Fig. 1:**
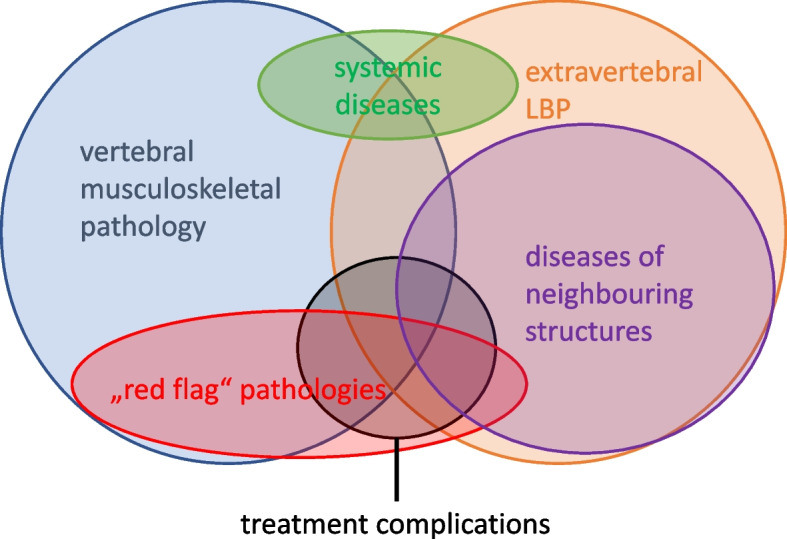
Overlap of definitions with bubble sizes approximately representing epidemiology. LBP = low back pain

While LBP caused by conditions such as abdominal aortic aneurysm, peripheral arterial disease [18–20], or renal calculus can unambiguously be classified as extravertebral or non-spinal diseases, in many instances, categorisation remains challenging. For example, determining whether conditions involving intramedullary tumours, metabolic diseases (e.g., spinal gout), or hip pathology [21] mimicking radiculopathy should be attributed to spinal or extravertebral causes of pain remains debatable (Fig. 1). At times, these conditions are collectively referred to as unusual or rare causes for LBP [22–34]. The term “extravertebral” or “non-spinal” LBP is anatomically incorrect since it refers only to the bony structures of the back. Currently, there is no consensus on a definition or terminology to classify serious LBP not typically covered by “red flags”. Red flags are warning signs related to pathologies like tumours, fractures, inflammations or infections of the spine [35–37].

The aim of this scoping review is to summarise what is known on the epidemiology and presentation of extravertebral or non-spinal LBP, in order to help clinicians assessing patients with LBP to recognise when it is appropriate to include this in the differential diagnosis.

## Methods

This is a scoping review conducted according to the PRISMA extension for scoping reviews (Appendix 1) [38]. Since PROSPERO does not register scoping reviews, a protocol was not registered. A scoping review was chosen due to several factors, including the absence of a universally accepted definition, the broad spectrum of diseases, and the limited existing literature on the topic. Furthermore, this methodology was selected to facilitate a comprehensive overview of the field, identify current research gaps, and provide recommendations for future research.

### Search strategy

**Fig. 2:**
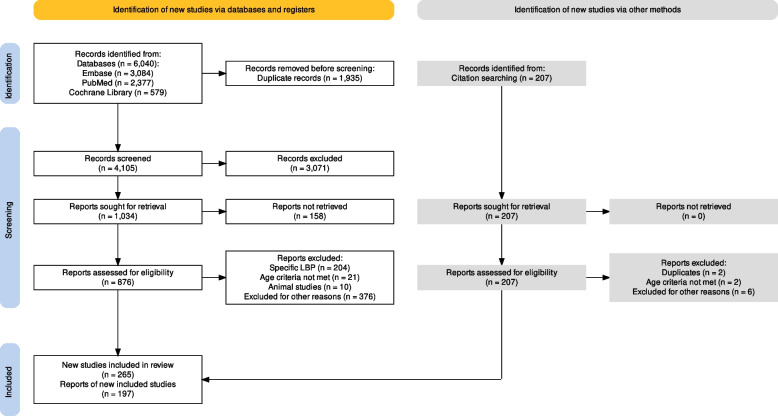
Search flow diagram of the literature review process for studies on extravertebral low back pain according to the PRISMA2020 Statement [39]. LBP = low back pain

The authors searched three electronic databases (MEDLINE, Embase, and the Cochrane Library). In 2001, Deyo and Weinstein published the first major narrative review of extravertebral causes of LBP [3]. Therefore, the search scope was limited to publications from 1 January 2001 to 31 December 2020. The detailed search strategy is outlined in Fig. 2, and the specific search terms used are available in Appendix 2. Where required, search terms were amalgamated using Boolean logic and database-specific filters. All publications available in either German or English languages were included.

### Study selection

There is no universally accepted definition allowing to separate “extravertebral” unambiguously from “vertebral” LBP (Fig. 1). Furthermore, the terminologies are not formulated clearly and are insufficient for classifying both included and excluded diseases. Alternatively, the terms “extraspinal” or “non-spinal” back pain are also used. During the review process, the authors encountered difficulties in finding an accurate definition due to overlapping terms and classification systems. Nevertheless, an attempt was made to classify the diseases related to low back pain.

Inclusion criteria comprised publications of case reports, case series, case-control studies, cohort studies, randomised controlled trials, observational studies, and reviews reporting low back pain as a symptom of non-primary vertebral/musculoskeletal disease in adults, including systemic diseases. The MeSH-terms are available in Appendix 2.

Exclusion criteria comprised publications with patients under the age of 18 and pain primarily reported in thoracic and cervical spine. Furthermore, “red flags” indicating pathologies, such as infections, rheumatic diseases, tumours, and fractures were excluded. A complete summary of excluded pathologies can be seen in Appendix 3.

After the removal of duplicates, two authors screened the titles and abstracts independently. The included articles were discussed among the authors according to relevance, data extraction, and quality. Dissents were solved by consensus. This was followed by a handsearch.

### Clinical guideline selection

All clinical guidelines listed in the most recent review of LBP guidelines [1] written in English or German language were reviewed to find recommendations regarding extravertebral LBP.

### Data extraction

Descriptive characteristics were extracted from each manuscript, including, author’s name(s), year of publication, country, study design and setting. Depending on the type of publication, further data was extracted.

For case reports and case series, extracted data included participant characteristics (sample size, sex, age, and co-morbidities), pain characteristics (acute vs chronic LBP, pain description, neurological and other symptoms), diagnostic characteristics (laboratory results, imaging, biopsy/other diagnostic, diagnostic confirmation, and physical examination) and differential diagnosis.

For case-control or cohort studies, the following data were extracted: data collection (retrospective/prospective), inclusion criteria, baseline, follow-up, LBP (acute/chronic), other symptoms, pain description, differential diagnosis and other information (e.g., epidemiological information, risk factors, and physical examination findings).

For reviews encompassing LBP in general, the following data points were extracted: classification, terminology, causes, estimated prevalence, symptoms pointing toward a non-spinal pathology, diagnostic values (e.g., patient history, physical examination, laboratory results, and imaging) and whether Weinstein and Deyo 2001 was cited or not. If specific pathologies were mentioned, only unique information about LBP associated with those diseases were extracted.

## Results

**Fig. 3:**
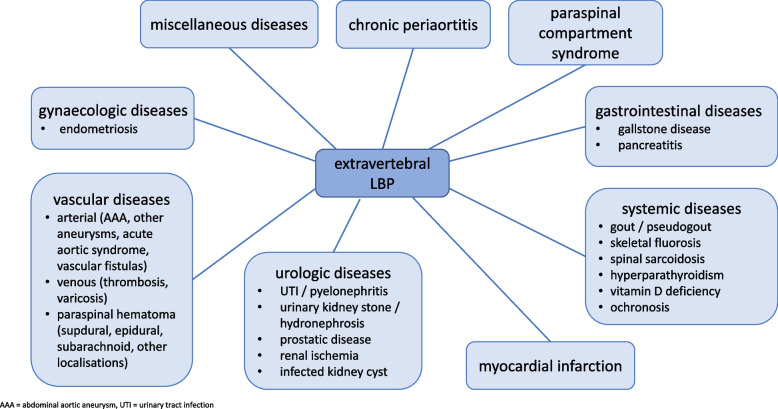
Overview: causes of extravertebral low back pain organised by pathologies. AAA = abdominal aortic aneurysm. LBP = low back pain. UTI = urinary tract infection

After elimination of duplicates and resolving disagreement between the reviewers, a total of 4105 manuscripts were screened. Additionally, a handsearch was carried out and a further 197 manuscripts were included in the review (Fig. 2), thereby bring the total number of manuscripts included in this review to 462. Various extravertebral causes of LBP were identified and are illustrated in Fig. 3.

### Description of studies

#### Case reports and case series

Various case reports mentioned LBP as part of the clinical presentation, but it often remained unclear, if LBP was the chief complaint. Case reports, where back pain was mentioned but no connection to the final diagnosis could be made or it seemed that back pain was a coincidence, were excluded. None of the case reports claimed adherence to the standards of reporting from CARE [40].

#### Case control and cohort studies

Only a few case-control or cohort studies were included. In these studies, various diseases, their therapies, and diagnostic methods were examined. If LBP was reported, comparisons were often made between pre- and post-interventional symptoms to draw conclusions regarding the association between pathology and LBP.

#### Narrative and systematic reviews

The content of narrative or systematic reviews often dealt with low back pain in general or in association with other pathologies, where LBP was a reported symptom. Reviews of other pathologies often omitted information regarding duration or localisation of LBP, while frequently including associated symptoms.

#### Guidelines

The included guidelines were examined with regards to the recommendations for dealing with extravertebral low back pain.

### Results of the guideline review

Guidelines featured in the latest review of clinical practice guidelines on LBP were examined by Oliveira et al. 2018 [41]. They presented a total of 15 guidelines across various countries, of which 11 guidelines available in German or English were reviewed. Four guidelines mentioned extravertebral, non-vertebral, or systemic causes of LBP. One guideline reported that an “alternative diagnosis” should be considered [42]. The rest did not mention the possibility of an extravertebral origin of low back pain.

### Results organised by pathology

#### Systemic diseases

##### Spinal gout

Gout is a systemic disease where monosodium urate crystal deposit in various joints, such as, facet, sacroiliac or interverbal joints as well as discs. Rheumatic diseases are pathologies indicated by red flags and usually refer to axial spondyloarthropathies excluding gout. Spinal or axial gout was first described in 1950 [43]. Since then, several case reports and case series have been published (Table 1). Toprover et al. published a review of 131 cases of spinal gout. We decided to disregard all case reports featured in their review in Table 1 [30, 44–52]. Furthermore, many of them were not within the time frame of this review. The majority (roughly 75%) had a history of gout. It is frequently concluded that axial gout is more common than assumed. However, no conclusions about the prevalence of axial gout can be drawn from the case reports and case series. The case series reveal a prevailing trend wherein a significant number of patients with axial gout have a confirmed diagnosis of gout, frequently accompanied by the presence of peripheral tophi. In most cases, the diagnosis was confirmed through intraoperative biopsies or fluoroscopy [43]. The case series conducted by de Mello et al. [53], stands out due to its investigation of spinal computed tomography (CT) scans in individuals who had a confirmed diagnosis of gout. Possible evidence of axial gout was found in 12/42 (29%) and peripheral tophi were associated with CT-findings suggestive for gout. The findings were not associated with current pain. Therefore, the claim that spinal gout is more frequent than assumed is weak, assuming that despite radiological findings, many patients with urate deposition in the spine are asymptomatic.

**Table 1:** Case reports of gout presenting with low back pain

**Author / year / country**	**Setting**	**Patient**	**Spinal symptoms**	**Extraspinal symptoms**	**Diagnostic confirmation / differential diagnosis**
Cardoso et al., 2014, Brazil [54]	ED send by GP	male, 55 y	chronic LBP, paraparesis	fever, no h/o gout, chronic kidney disease, diabetes	elevated ESR, CT-guided biopsy
Lu et al., 2017, China [55]	ED	male, 68 y	chronic LBP	no h/o gout	biopsy during surgery
Wang et al., 2017, China [56]	ED	male, 62 y	chronic LBP	h/o gout	biopsy during surgery
Ribeiro et al., 2018, Portugal [57]	ED	male, 77 y	acute LBP, paraparesis	h/o gout	biopsy during surgery
Qin et al., 2018, China [58]	ED	male, 56 y	subacute LBP	h/o gout	biopsy
Alqatari et al., 2018, Ireland [59]	specialist care	male, 55 y	chronic LBP	h/o gout, tophi and psoriatic arthropathy with non-response to TNF-blocker	elevated ESR, imaging (MRI, CT)
Zou et al., 2019, China [60]	specialist care	male, 55 y	acute LBP	intermittent claudication, h/o gout	elevated ESR, biopsy during surgery
Chen et al., 2020, China [61]	ED	male, 55 y	chronic LBP	no h/o gout	biopsy during surgery

*CT* computer tomography*, ED* emergency department*, ESR* erythrocyte sedimentation rate*, GP* general practitioner, *h/o* history of*, LBP* low back pain*, MRI* magnetic resonance imaging*, TNF-blocker* tumour necrosis factor blocker*, y* years

##### Pseudogout

Pseudogout is a rare disease with calcium-pyrophosphate deposition, which can affect any joint, including facet joints, causing inflammatory arthritis. Five case reports and case series of pseudogout presenting with low back pain (Table 2) were included. Symptoms are non-specific and diagnosis is usually made incidentally due to suspicious findings leading to operative exploration with biopsies. Only one patient had a history of pseudogout.

**Table 2:** Case reports or case series of pseudogout presenting with low back pain

**Author / year / country**	**Setting**	**Patient(s)**	**Presentation / clinical history**	**Diagnostic confirmation**
Fujishiro et al., 2002, Japan [62]	ED	female, 71 y	acute LBP, h/o pseudogout, elevated ESR and CRP	joint aspiration
Gadgil et al., 2002, UK [63]	ED	female, 67 y	chronic LBP, sciatica, CT-Scan with calcified cyst with nerve compression	biopsy during surgery
Mahmud et al., 2005, UK [44]	not reported	6 patients (4 with gout, 2 with pseudogout): female, 70 y	subacute LBP, sciatica, MRI-Scan with cyst and nerve compression	biopsy during surgery
		male, 81 y	chronic LBP, claudication, MRI-scan with severe spinal stenosis due to deformity and cysts	
Namazie et al., 2012, New Zealand [64]	not reported	female, 69 y	subacute LBP, h/o scleroderma, CT-Scan with large, calcified mass	biopsy
Shen et al., 2019, China [65]	ED	male, 53 y	chronic LBP, h/o gout, elevated ESR and CRP, CT-Scan with mass and bone destruction of facet joints	biopsy

*CRP* c-reactive protein*, CT* computer tomography*, ED* emergency department*, ESR* erythrocyte sedimentation rate*, h/o* history of, *LBP* low back pain*, MRI* magnetic resonance imaging*, y* years

##### Skeletal fluorosis

Two case reports were identified where the diagnosis of skeletal fluorosis, contributing to the onset of chronic metabolic bone disease, was associated with chronic LBP (Table 3). Skeletal fluorosis is a rare disease caused by increased ingestion of fluoride. It is endemic in some parts of Asia (e.g., China, India), where elevated fluoride concentrations are found in soil and water. Industrial exposure, accidental ingestion of fluoride containing medication or toothpaste and substance abuse are other possible causes. Mottling of teeth is a clinical sign of excessive exposure to fluoride as an infant. The condition is typically diagnosed incidentally based on osteosclerosis and ligamentous calcification on X-ray. There is no established treatment.

**Table 3:** Case reports of skeletal fluorosis presenting with low back pain

**Author / year / country**	**Setting**	**Patient**	**Clinical history**	**Diagnostic confirmation**	**Comment**
Peicher et al., 2017, USA [66]	not reported	male, 33 y	progressive LBP for 3 years	X-ray (generalised osteosclerosis)	fluoride inhalation (huffing of cleaner)
Shetty et al., 2015, India [67]	not reported	male, 35 y	compressive myelopathy with paraparesis with LBP for 3 years, mottling of teeth	X-ray (generalised osteosclerosis and calcifications of the longitudinal ligament)	most likely endemic due to increased fluoride in drinking water

*LBP* low back pain,* y* years

##### Spinal sarcoidosis

Sarcoidosis is a multisystem granulomatous disease, which most commonly affects the lung. It is estimated that 1-3% of patients with sarcoidosis have some form of osseous disease, which is mostly asymptomatic. A total of 6 case reports highlighting spinal sarcoidosis associated with LBP were included (Table 4). Back pain can be caused by either spinal osseous involvement or medullary disease. Improvement following treatment, e.g., with corticosteroids, has been reported. In some patients, the diagnosis of sarcoidosis was pre-existing, while in other cases, suspicious findings on magnetic resonance imaging (MRI) prompted bone biopsies that lead to diagnosis [68, 69]. The radiological findings, however, lack specificity. Given the array of potential differential diagnoses encompassing osseous metastasis, myeloma, lymphoma, tuberculosis, and osteomyelitis, the verification of the diagnosis primarily relied on bone biopsy. No discernible clinical clue beyond a pre-existing diagnosis of sarcoidosis were evident.

**Table 4:** Case reports of sarcoidosis presenting with low back pain

**Author / year / country**	**Setting**	**Patient**	**Spinal symptoms**	**Extraspinal symptoms**	**Diagnostic confirmation**
Ludwig et al., 2003, USA [68]	not reported	female, 51 y	LBP (no duration reported)	-	MRI, PET-Scan, bone biopsy
Ashamalla et al., 2016, USA [69]	not reported	female, 60 y	LBP (no duration reported)	h/o Crohn’s disease	MRI, PET-Scan, bone biopsy
Rice et al., 2011, UK [70]	not reported	female, 62 y	LBP (no duration reported)	h/o sarcoidosis	MRI, bone biopsy, response to therapy with corticosteroids
Valencia et al., 2009, USA [71]	not reported	female, 48 y	LBP (no duration reported)	diffuse arthralgia, abdominal pain, dyspnoea	MRI, PET-Scan, bone biopsy
Packer et al., 2005, USA [72]	specialist care	male, 47 y	chronic LBP	h/o pulmonary sarcoidosis, weight loss	MRI, PET-Scan, bone biopsy
Barazi et al., 2008, UK [73]	not reported	female, 44 y	chronic LBP, altered sensation in the lower limbs	-	MRI, bone biopsy

*h/o* history of*, LBP* low back pain, *MRI* magnetic resonance imaging*, PET* positron emission tomography*, y* years

##### Hyperparathyroidism

Hyperparathyroidism is another rare condition that can arise from either primary origin, such as, adenomas (and rarely carcinomas), or as a secondary manifestation of end stage kidney disease (ESKD). Presenting complaints typically include general and non-specific symptoms such as weakness, thirst, polyuria, weight loss, and musculoskeletal pain. Only five case reports describing hyperparathyroidism as a cause of LBP were found and included (Table 5). Up to 3% of individuals with hyperparathyroidism will develop brown tumours (osteitis fibrosa cystica), which are neoplastic and can cause LPB and neurological symptoms due to compression if located in the spine. The presence of the mass lesion is typically identified through imaging as a consequence of neurological symptoms [74]. Symptoms suggesting the need to consider hyperparathyroidism in the differential diagnosis include a patient’s previous history of urolithiasis and ESKD in the context of chronic LBP. The diagnosis is likely with elevated serum calcium and alkaline phosphatase, low serum level of phosphate and confirmed by measuring serum hyperparathyroid hormone.

**Table 5:** Case reports and reviews of hyperparathyroidism presenting with low back pain

**Author / country / year**	**Design / setting**	**Patient(s)**	**Spinal symptoms**	**Extraspinal symptoms**	**Diagnostic confirmation**
Khalatbari et al., 2014, Iran [74]	CR and	4 cases with brown tumours (50% females), but only 2 in the lumbar spine	chronic LBP and progressive weakness of the lower extremity, radicular pain	muscular weakness	laboratory work up, surgery
	review of 15 previously reported CR / not reported	15 cases of brown tumour, only 6 in the lumbar spine			
Hoshi et al., 2008, Japan [75]	CR / outpatient clinic	female, 23 y	chronic LBP	h/o urolithiasis	laboratory work up, biopsy
Yu et al., 2012, Taiwan [76]	CR / not reported	female, 28 y	chronic LBP	weakness, thirst, constipation, h/o urolithiasis	laboratory work up, surgery
Anastasilakis et al., 2011, Greece [77]	CR / outpatient clinic	female, 70 y	chronic LBP, kyphosis	weakness, arthralgias	laboratory work up, surgery
Wiederkehr, 2020, USA [78]	CR / not reported	female, 33 y	chronic LBP	end stage kidney disease	laboratory work up, biopsy

*CR* case report, *h/o* history of, *LBP* low back pain,* y* years

##### Vitamin D deficiency / insufficiency

Osteomalacia arises from a deficiency in vitamin D, an essential substance for maintaining bone health, consequently leading to the manifestation of LBP [79]. However, most people with low 25-hydroxyvitamin (25-OH) D3 (calcidiol) level do not develop osteomalacia. A connection between low calcidiol and LBP was initially made by Al Faraj et al. [80], in an observational study, which subsequently resulted in numerous studies (Table 6). The documented deficiency of calcidiol in 83% of individuals experiencing lower back pain (LBP), along with the observed cessation of LBP in all patients with low levels following supplementation within an unspecified time frame, prompted the initiation of cross-sectional and case-control studies into the potential correlation between LBP and vitamin D insufficiency or deficiency. (Table 6). Most studies, except one [81], concluded that vitamin D deficiency was contributing to LBP and even recommended screening patients with chronic LBP. However, no specific symptoms, which could help to identify patients with vitamin D deficiency, were described. Additionally, the definitions of vitamin D deficiency or insufficiency were heterogenous. A Cochrane review on the effectiveness of vitamin D for chronic pain, including LBP, found no consistent evidence for the effectiveness of vitamin D substitution [82].

**Table 6:** Studies reporting on vitamin D and low back pain

**Author / country / year**	**Design / setting**	**Patients**	**Inclusion criteria / exclusion criteria**	**25-OH Vitamin D3 status**	**Main finding and conclusion**
Al Faraj et al., 2003, Saudi Arabia [80]	observational study / specialist clinic	360 patients (90% female)	LBP, 15-52 years (90 % female), no red flag pathologies, no renal impairment or chronic liver disease	299 (83%) had low 25-OH-vit D (< 22.5 nmol/L)	substitution of vitamin D: cessation of LBP (95%) (100% with low vitamin D and 69% with normal vitamin D level)
Rkain et al., 2013, Morocco [83]	case control study / specialist clinic	105 cases / 44 controls (100% female)	postmenopausal women 42-80 years with LBP, no red flag pathologies	79 % cases vs 61.4 % controls had low vitamin D (<20 ng/ml)	association between vitamin D deficiency and chronic LBP in Moroccan post-menopausal women
Johansen et al., 2013, Denmark [81]	cohort study / specialist clinic	902 patients screened; 152 patients included (48% female)	chronic LBP 19-64 years, no specific LBP, disk herniation or spinal stenosis	99 (65.1%) had normal vitamin D levels (> 50 nmol/L), 36 (23.7%) had mild vitamin D deficiency (25–50 nmol/L) and 17 (11.2%) patients had moderate/severe deficiency (< 25 nmol/L)	vitamin D deficiency not more common than in the general population, no relation to clinical symptoms
Baykara et al., 2014, Turkey [84]	case control study / specialist clinic	60 cases (62% female) / 30 controls (63% female)	chronic LBP, 20-50 years, no red flag pathologies	53 (88%) cases & 11 (37%) controls had low vitamin D (<20 ng/ml)	vitamin D level: significantly lower in the patient group
Rehman et al., 2020, Pakistan [85]	cross-sectional observational study / outpatient clinic	182 patients (36% female)	LBP, no exclusion criteria described	20 (11%) with vitamin D deficiency (< 50 ng/ml), 132 (74%) with vitamin D insufficiency (< 20 ng/ml)	vitamin D: contributing to LBP; conclusion not justified by study design.
Bahinipati et al., 2020, India [86]	cross-sectional observational study / specialist clinic	196 patients (61% female)	chronic LBP	35 (18%) had normal vitamin D levels (> 30 ng/ml), 59 (30%) had insufficiency (21–29 ng/ml) and 52 (52%) had deficiency (< 20 - ng/ml)	pain intensity measured by visual analogue scale score: significantly higher with decrease in vitamin D levels.

*LBP* low back pain, *25-OH-vit D* 25-hydroxyvitamin D

##### Ochronosis / Alkaptonuria

Ochronosis, also known as alkaptonuria, is a rare autosomal recessive genetic disorder leading to accumulation of homogentisic acid in the body. Ochronotic arthritis gives rise to chronic back pain typically occurring during the fourth and fifth decade of life, mimicking ankylosing spondylitis including the marked spine stiffness. However, this condition commonly extends its involvement to other joints as well. A total of 12 case reports were found, all focussing on chronic LBP (Table 7). Diagnostic signs, such as pigmentation of the sclera and ear, and darkening of morning urine, were not always present. Intervertebral disc calcification on imaging can be considered pathognomonic and was observed in all case reports. The diagnosis was confirmed with measurement of homogentisic acid in the urine or sometimes from specimens obtained during surgery [87–89]. Only a few patients had been previously diagnosed during childhood, and one patient received a diagnosis during prior surgery.

**Table 7:** Case reports^a^ of ochronosis presenting with chronic low back pain

**Author / country / year**	**Setting**	**Patient**	**Spinal symptoms**	**Extraspinal symptoms**
Capkin et al., 2007, Turkey [90]	outpatient clinic	male, 50 y	stiffness, restriction of movements	yellowish green ochronotic pigmentation of cartilage & ears, reduced chest expansion
Al-Mahfoud et al., 2008, UK [91]	outpatient clinic	female, 58 y	not reported	darkening of the urine, diagnosis established previously during surgical intervention (hip replacement)
Grasko et al., 2009, Australia [92]	outpatient clinic	male, 38 y	progressive severe LBP, decreased lumbar flexion	hip pain, urine discolouration changing to black after prolonged standing, renal colic, passing black calculus, angular pigmentation in sclera and bluish discolouration of auricle
Ahmed et al., 2010, Pakistan [93]	not reported	male, 38 y	progressively increasing stiffness, reduced ability to bend forward	degenerative changes of both radiocarpal joints and metatarso-phalangeal joints
Effelsberg et al., 2010, Switzerland [94]	outpatient clinic	male, 38 y	limited spine mobility, slight scoliosis	none, diagnosis of ochronosis established in childhood
Amiri et al., 2012, Iran [95]	hospital	female, 54 y	severe low back pain and limitation of motion	discolouration of the sclera, knee pain, renal colic and subsequently passing black urine
Sebastian et al., 2012, South Africa [28]	rheumatology clinic	male, 46 y	limited extension of the spine	bluish discolouration of the pinnae bilaterally, 2 mm bilateral blue nodules between the joints on the thumbs
Seidhamed et al., 2012, Qatar [32]	emergency department	male, 45 y	not reported	generalised joint pain
Mirzashahi et al., 2016, Iran [87]	outpatient clinic	male, 51 y	weakness of both lower limbs	none
Etzkorn et al., 2014, USA [96]	not reported	female, 55 y	decreased range of motion, morning stiffness, scoliosis	yellowish green ochronotic pigmentation of cartilage and ears, reduced chest expansion
Bozkurt et al., 2017, Turkey [89]	outpatient clinic	male, 47 y	tingling, and numbness and weakness in both legs, increased kyphosis	darkening of the urine
Alkasem et al., 2019, Irak [88]	not reported	female, 56 y	severe kyphosis, inability to stand straight	hip pain, discoloration of urine that changed to black after urination, three angular pigmentations in sclera and bluish discolouration of auricle

*LBP* low back pain, y years

^a^all reported chronic low back pain

### Vascular diseases - arterial

#### Abdominal aortic aneurysm

An aneurysm is an outward bulging of the vessel wall usually caused by wall weakness. Most aneurysms develop slowly and are initially asymptomatic. Symptoms of abdominal aortic aneurysm (AAA), such as LBP and abdominal pain, can vary depending on the location and type, i.e., acute versus chronic contained ruptured. Several case reports, three cohort studies and nine narrative reviews were identified documenting AAA featuring LBP within the clinical manifestation (Table 8). Mainly middle-aged to older male individuals were affected. Initially 8-18 % of non-inflammatory AAA are symptomatic, while patients with inflammatory AAA exhibit symptoms in 65 to 90% of cases [97]. The majority of symptomatic patients report chronic LBP, occasionally characterised by a progressive exacerbation. Acute or subacute back pain presentations are also possible. The presence of LBP as part of the clinical presentation of an AAA ranged from 32% [98] to 72% [99]. The co-occurrence of LBP and abdominal pain was 29.4% [100]. Furthermore, abdominal pain and a pulsatile abdominal mass in patients with LBP were indicative of the presence of an abdominal aortic aneurysm [97, 99–123]. The presence of the complete triad of LBP or abdominal pain with hypotension and a pulsatile abdominal mass is rather low at 21% [100] and usually observed during rupture [117, 118, 124]. In some cases, history of smoking was reported [97, 102–104, 106, 108, 110, 111, 115, 117, 119, 121–123, 125–132]. Other common risk factors are atherosclerotic disease, hypertension, positive family history for AAA and other aneurysms, collagen vascular disease and Marfan and Ehlers-Danlos-syndromes [106, 117, 119, 121–123, 133].

**Table 8:** Abdominal aortic aneurysm presenting with back pain

**Author / year / country**	**Design / setting**	**Patient(s)**	**Spinal symptoms**	**Extraspinal symptoms**	**Diagnostic confirmation**	**Other information**
Al-Koteesh et al., 2005, UK [134]	CR / not reported	male, 46 y	acute LBP, numbness (upper left thigh)	-	laparotomy	type: chronic contained rupture of AAA, similar attacks for past years
Arici et al., 2012, Italy [135]	CR+NR / not reported	male, 73 y	chronic LBP	uncontrolled hypertension	spinal MRI	type: chronic contained rupture of AAA; review: most prevalent symptom = LBP (78.6%)
Aydogan et al., 2008, Turkey [101]	CR / hospital	male, 51 y	chronic LBP	-	MRI	type: chronic contained rupture of AAA; physical examination: pulsatile mass
Caynak et al., 2008, Turkey [103]	CR / hospital	male, 75 y	chronic LBP, increased	-	CTA	type: chronic contained rupture of AAA; h/o smoking; physical examination: palpable mass
Copetti et al., 2017, Italy [104]	CR / ED	male, 85 y	chronic LBP	-	abdominal CT scan	type: chronic contained rupture of AAA; h/o smoking; physical examination: pulsatile abdominal mass
Dobbeleir et al., 2007, Belgium [105]	CR / ambulatory care	male, 79 y	acute LBP	dyspnoea	CT	type: chronic contained rupture of AAA; physical examination: palpable mass
Gandini et al., 2007, Italy [136]	CR / hospital	male, 69 y	LBP (no duration reported)	fever	msC T-guided needle aspiration	type: chronic contained rupture of AAA
Martos et al., 2018, Spain [137]	CR / ambulatory care	male, 73 y	chronic LBP	-	plain radiology	type: AAA without rupture
Henderson et al., 2003, USA [125]	CR / ED	male, 67 y	acute LBP	-	CT	type: rupture of AAA; h/o smoking (10 cigarettes per day)
Horowitz et al., 2005, Israel [138]	CR / not reported	male, 82 y	LBP (no duration reported)	-	MRI lumbar spine	type: AAA without rupture
Jimenez Viseu Pinheiro et al., 2014, Spain [126]	CR / hospital	female, 75 y	chronic LBP, difficulties walking properly	-	CTA	type: chronic contained rupture of AAA; 45-pack-year history
Kim et al., 2013, Korea [139]	CR / hospital	male, 73 y	chronic LBP, claudication, left leg paralysis	pitting oedema of both legs	abdominal contrast-enhanced CT	type: AAA without rupture
Lai et al., 2008, Taiwan [140]	CR / not reported	male, 67 y	chronic LBP	-	CT and radiography	type: chronic contained rupture of AAA
Lucas et al., 2018, Portugal [141]	CR / ED	male, 74 y	acute LBP, radiating	-	abdominal ultrasonography	type: AAA without rupture
Mechelli et al., 2008, Italy [127]	CR / ambulatory care	male, 38 y	subacute LBP, unable to run	frequent awakening	CT	type: AAA without rupture; h/o smoking (10 cigarettes per day)
Moos et al., 2009, USA [106]	CR / ambulatory care	female, 31 y	chronic LBP	chronic abdominal pain	CT aortogram	type: AAA without rupture; h/o smoking, physical examination: pulsatile abdominal mass
Nakano et al., 2013, Japan [142]	CR / not reported	male, 62 y	acute LBP, right leg pain	-	open biopsy	type: chronic contained rupture of AAA
Nguyen et al., 2018, Vietnam [107]	CR / not reported	male, 49 y	subacute LBP	-	biopsy, spinal surgery	type: chronic contained rupture of AAA; physical examination: pulsatile abdominal mass
Patel et al., 2005, USA [124]	CR / not reported	male, 69 y	acute LBP, radiating, weakness right leg	-	CTA	type: AAA without rupture
Seçkin et al., 2006, Turkey [143]	CR / not reported	female, 38 y	acute LBP, difficulty walking, right buttock pain	-	MR	type: AAA without rupture
Tan et al., 2019, Singapore [128]	CR / not reported	male, 81 y	acute LBP	hoarseness	CT	type: AAA without rupture; 4-pack / year-history (has quit 35 years ago)
Terai et al., 2015, Japan [144]	CR / not reported	male, 89 y	LBP (no duration reported), bilateral weakness in lower extremities	-	CT	type: AAA without rupture
Wyngaarden et al., 2014, USA [108]	CR / not reported	male, 58 y	acute LBP	abdominal pain, difficulty falling asleep	CT	type: AAA without rupture; h/o smoking early 1970s, abdominal pulsation
Walker et al., 2017, USA [129]	CR / ED	male, 57 y	acute LBP	weight loss	MRI	type: chronic contained rupture of AAA; 20 pack-year history
Jukovic et al., 2016, Serbia [130]	CR / hospital	male, 60 y	chronic LBP	-	CT	type: chronic contained rupture of AAA; h/o smoking
Alshafei et al., 2015, Bahrain [131]	CR / not reported	male, 63 y	chronic LBP, 1-year history of bilateral intermittent claudication, later: rigors	later: fever, elevated white cell count and a rise of CRP	surgery	type: chronic contained rupture of AAA; h/o smoking
Bogie et al., 2008, The Netherlands [109]	CR / ED	male, 55 y	acute LBP, sudden onset, loss of motor function in both legs	nausea, abdominal pain, 3 days previously: abdominal discomfort and nausea	CT	type: AAA without rupture; no h/o smoking; used antihypertensive medication
Chieh et al., 2003, USA [145]	CR / ambulatory care	female, 23 y	back pain (no duration or localisation reported)	dyspnoea on exertion	CT	type: multiple isolated aneurysms in Takayasu’s aortitis
De Boer et al., 2010, The Netherlands [110]	CR / ambulatory care	male, 74 y	chronic LBP (slowly developing, 5/10 pain scale, radiating to the knee, worsening with walking, standing, stair climbing, diminished when lying down)	later: abdominal pain while lying supine, aggravated by lying – sharp intermittent pain	ultrasound, surgery	type: AAA without rupture; previous treatments without permanent results, heavy prior cigarette use but stopped 15 years ago
Defraigne et al., 2001, Belgium [146]	CR / not reported	5 patients (1 with LBP): male, 73 y	subacute LBP (sciatic pain), crural neuropathy	-	CT, ultrasound	type: chronic contained rupture of AAA; no inflammatory syndrome, no vertebral erosion
Dorrucci et al., 2001, Italy [147]	CR / hospital	female, 87 y	subacute back pain (no localisation reported)	mild jaundice, abdominal fullness, dyspepsia	CT, surgery	type: chronic contained rupture of AAA
Lombardi et al., 2016, Brazil [113]	CR / ED	male, 66 y	back pain (no duration or localisation reported), exacerbation, unsteady gait	lower left quadrant pain	CT	type: chronic contained rupture of AAA
Sakai et al., 2007, Japan [148]	CR / not reported	male, 72 y	chronic LBP	-	CT	type: chronic contained rupture of AAA
Whitwell et al., 2002, UK [149]	CR / ED	male, 70 y	acute back pain (no localisation reported), loss of power and sensation in both legs	collapse	laparotomy	type: AAA with rupture
Kamano et al., 2005, Japan [150]	CR / hospital	male, 40 y	LBP (no duration reported) (mild), bilateral lower limb paralysis	-	surgery	type: AAA with rupture
Hocaoglu et al., 2007, Turkey [115]	CR / not reported	male, 67 y	chronic LBP (radiating to the abdomen and shoulder, pressure sensation towards rectum, increasing at night), difficulty walking	flushing, sweating, abdominal distension associated with pain, weight loss (3 kg in 3 weeks)	ultrasound, CT / MRI	type: AAA without rupture; h/o smoking, LBP resistant to medication and rest
Metcalfe et al., 2016, USA [100]	CHS / hospital	85 (17.6% female), median age: 76 y	LBP (54.1%) (no duration reported)	abdominal pain (61.2%), groin pain (11.8%), atypically distributed pain (8.2%), loin pain (4.7%), palpable AAA (70%), distracting symptoms (38.8%), hypotension (37.6%), LOC (36.5%), tachycardia (18.8%), gastrointestinal symptoms (17.6%)	-	combination of abdominal and LBP (29.4%), complete triad of back or abdominal pain, hypotension, and palpable mass (21%)
Takeyachi et al., 2006, Japan [98]	CHS / hospital	34 (5.9% female), median age: 72 y	LBP (32%) (no duration reported)	not reported	-	-
Tsuchie et al., 2013, Japan [151]	CHS / hospital	23 (34.8% female), mean age: 77 y	LBP (52.2%) (no duration reported)	not reported	-	-
Crawford et al., 2003, Australia [116]	NR / -	-	mostly asymptomatic at the beginning; most common symptom: LBP (no duration reported)	fullness or pulsations in the abdomen, abdominal pain	diagnostic methods: physical examination (tender, palpable, pulsatile abdominal mass and bruit)	91% with symptoms at first presentation
Anderson et al., 2001, USA [117]	NR / -	age: 50-60 y, male:female = 5:1	back pain radiating to abdomen, back pain in case of vertebral body erosion (no duration or localisation reported)	palpation of a pulsatile abdominal mass, vague abdominal pain with radiation to flank, groin; early satiety / nausea / vomiting, gastrointestinal bleeding (in case of fistula), lower extremity ischaemia, venous thrombosis, flank / groin pain	diagnostic methods: US = preferred first method, diagnostic confirmation: CT / CTA or MRI / MRA	risk factors: cigarette use, hypertension, coronary artery disease, COPD, hyperlipidaemia
Assar et al., 2009, USA [118]	NR / -	-	severe back pain (no duration or localisation reported), chronic contained rupture: chronic LBP may radiate to the groin, maybe lumbar vertebral erosion, lumbar spondylitis-like symptoms; unusual: transient lower limb paralysis; chronic contained rupture: left lower limb weakness or neuropathy, crural neuropathy	right hypochondrial pain, nephroureterolithiasis, groin pain, testicular pain, testicular ecchymosis (blue scrotum sign of Bryant), iliofemoral venous thrombosis, inguinoscrotal mass mimicking a hernia; chronic contained rupture: left psoas muscle haematoma and obstructive jaundice	diagnostic methods: physical examination (pulsatile epigastric mass); diagnostic confirmation: CT	-
Isselbacher et al., 2005, USA [119]	NR / -	-	acute LBP, typically steady and gnawing lasting hours to days	pain (hypogastrium)	diagnostic methods: physical examination (pulsatile abdominal mass), ultrasound, CT / CTA, MRI	risk factors: cigarette use, age, hypertension, hyperlipidaemia, atherosclerosis, positive family history for AAAs, male:female = 10:1, abrupt onset associated with rupture
Kumar et al., 2017, USA [120]	NR / -	-	back pain (no duration or localisation reported)	mostly asymptomatic and often incidentally detected; unruptured aneurysms (uncommonly abdominal pain or a pulsatile mass), ruptured aneurysms (severe abdominal pain, hypotension and shock, high mortality: 59-83%)	diagnostic methods: ultrasound, CT / CTA, MRA, DSA	-
Metcalfe et al., 2011, UK [133]	NR / -	-	back pain (no duration or localisation reported)	from asymptomatic to showing overt signs of rupture; pain in the abdomen or loin; distal embolisation	diagnostic methods: physical examination (pulsatile mass), ultrasound	risk factors: age, positive family history (not otherwise specified), male sex, cigarette use, hypertension, ethnicity (white > Asian), diabetes)
Sakalihasan et al., 2005, Belgium [122]	NR / -	male: 1.3-8.9%, female: 1.0-2.2%	LBP, recent onset, severe (no duration reported)	non-ruptured: generally asymptomatic, essentially diagnosed incidentally; chronic vague abdominal pain, ureterohydronephrosis; ruptured: sudden-onset pain in the mid-abdomen or flank, shock, pulsatile abdominal mass	diagnostic methods: physical examination (pulsatile abdominal mass), CT / CTA, MRI / MRA, aortography	risk factor: cigarette use, familial clustering; causes: trauma, acute infection, chronic infection, inflammatory diseases, connective tissue disorders (Marfan syndrome, Ehlers-Danlos-syndrome)
Rogers et al., 2004, USA [123]	NR / -	4-8% older than 65 y have an AAA	back pain (no duration or localisation reported)	chest pain and neurological abnormalities, chest pain in association with abdominal pain and chest pain that radiates into the back	-	reasons for delayed diagnosis: absence classic triad; risk factors: atherosclerotic diseases, advanced age, cigarette use, male sex, hypertension, strong familial association, patients with connective tissue disorders (Ehlers-Danlos, Marfan), white race; DDx: urological diseases, gastrointestinal bleeding, neuropathy, diverticulitis
Winters et al., 2006, USA [9]	NR / -	male:female = 10:1, advanced age	more commonly: back pain, lower-extremity paraesthesia	triad of hypotension, abdominal pain, and a pulsatile abdominal mass (< 50% of patients); flank pain, left lower-quadrant pain, syncope	diagnostic methods: physical examination (palpable pulsatile mass, abdominal bruit, diminished lower-extremity pulses, tender left lower-quadrant mass)	risk factors: hypertension, h/o tobacco use, hyperlipidaemia, atherosclerotic vascular disease, diabetes, connective tissue disorders, positive family history for AAA

*AAA* abdominal aortic aneurysm*, CHS* cohort study, *COPD* chronic obstructive pulmonary disease*, CR* case report*, CRP* c-reactive protein *CT* computed tomography, *CTA* computed tomography angiography*, DSA* digital subtraction angiography *ED* emergency department,* h/o* history of, *LBP* low back pain, *LOC* loss of consciousness, *MR* magnetic resonance*, MRA* magnetic resonance angiography, *MRI* magnetic resonance imaging*, msCT* multi-slice computed tomography, *NR* narrative review, *US* ultrasound,* y* years

In summary, in middle-aged and elderly males with chronic back pain and a pulsatile mass, abdominal pain, or other present risk factors, an AAA should be considered. The median time to diagnosis of an AAA is 7.3 years [99], with imaging studies (CT, MRI) typically used to confirm the diagnosis. In patients presenting with LBP as chief complaint and without other accompanying symptoms, an AAA is usually an incidental finding in lumbar radiographs [124]. Subsequent differential diagnoses include spinal tumours, metastasis, retroperitoneal tumours, iliopsoas muscle abscess, rheumatoid arthritis, osteoporosis and osteomalacia [103], especially when AAA leads to vertebral erosion.

#### Other aneurysms

A total of twelve case reports and a cohort study revealed instances of patients with aneurysms in locations other than the abdominal artery experiencing LBP as part of their clinical presentation (Table 9). Visceral artery aneurysms, for example, account for 1-2 % of non-aortic aneurysms. Of these, 60% affect the splenic artery [152]. Here, LBP was mostly described as acute pain [25, 153–159]. Extraspinal symptoms varied depending on the location of the aneurysm. For example, a splenic artery aneurysm showed gastrointestinal symptoms [152], while an aneurysm of the artery of Adamkiewicz showed neurological/vegetative symptoms [153]. The aetiology of non-aortic aneurysms is diverse and also includes infections, such as Takayasu arteritis, albeit rarely [154]. Moreover, additional underlying diseases can further contribute. For example, the majority of intercostal artery aneurysms arise in association with neurofibromatosis.

**Table 9:** Other aneurysms presenting with back pain

**Author / year / country**	**Design / setting**	**Patient(s)**	**Affected artery**	**Spinal symptoms**	**Extravertebral symptoms**	**Diagnostic confirmation**
Hanschke et al., 2002, Germany [152]	CR / hospital	male, 46 y	splenic artery	chronic LBP, left sided, analgesic resistant	feeling full, stool irregularities	CT
Iihoshi et al., 2011, Japan [153]	CR / hospital	female, 60 y	artery of Adamkiewicz	acute LBP, severe, left lower limb pain	headache, nausea	CT, DSA
Kellner et al., 2019, Germany [25]	CR / hospital	male, 38 y	7. and 8. intercostal artery	acute LBP, immobilisation, increase in vigilance	vomiting, general malaise	CT
Matsumoto et al., 2015, Japan [154]	CR / hospital	female, 51 y	superior mesenteric artery	acute LBP	sustained fever	angiography
Nakamura et al., 2020, Japan [155]	CR / not reported	male, 66 y	artery of Adamkiewicz	acute LBP, progressive posterior cervical pain	fever	DSA
Nogueira et al., 2010, USA [156]	CR / not reported	male, age not reported	lateral sacral artery	acute LBP, lower extremity paraesthesia, weakness, numbness of the genitalia	urinary hesitance	angiography
Ferrero et al., 2001, Italy [160]	CR / hospital	male, 43 y	splenic artery	back pain (no duration or localisation reported)	severe state of shock, abdominal pain	CT
Takebayashi et al., 2020, Japan [157]	CR / hospital	female, 67 y	ruptured posterior spinal artery aneurysm	acute LBP, worsened with movement, right thigh pain	sudden nausea	MRI + CE-CT
Bushby et al., 2010, Australia [159]	CR / ED	male, 67 y	iliac artery aneurysm	acute LBP, left sided, radiation into the left leg, numbness, weakness	weight loss (10 kg in 3 months), no bowel movements since symptom onset	CT
Bell et al., 2014, USA [158]	CR / ED	female, 68 y	ruptured posterior spinal artery pseudoaneurysm	acute LBP (severe, sharp, developed suddenly during physical exertion)	-	MRI/MRA
Caglar et al., 2005, Turkey [161]	CR / ED	female, 74 y	ruptured posterior spinal artery of the conus medullaris	LBP (severe, no duration reported), radiation into the right leg	-	DSA, histopathological examination
Gonzalez et al., 2005, USA [162]	CR / hospital	4 (all male), mean age: 56 y	spinal aneurysms	acute onset of back pain (no localisation reported), paraesthesia of lower extremities (sometimes bilateral), radicular pain	presented with ruptured aneurysms, spinal SAH concomitant headache (bilateral)	-
Knol et al., 2002, Belgium [163]	CHS / hospital	51 (2% female), mean age: 69.1 y	aortic or iliac	39% acute LBP (or abdominal pain)	12% haemorrhagic shock, 49% haemorrhagic shock and abdominal pain or LBP	-

*CE-CT* contrast enhanced computed tomography*, CHS* cohort study, *CR* case report, *CT* computed tomography*, DSA* digital subtraction angiography*, ED* emergency department*, LBP* low back pain*, MRA* magnetic resonance angiography*, MRI* magnetic resonance imaging*, SAH* subarachnoid haemorrhage,* y* years

#### Acute aortic syndrome

The acute aortic syndrome includes pathologies, such as aortic dissection (AD), intramural haematoma (IMH), and penetrating aortic ulcer (PAU). An aortic dissection is a tear in the inner wall of the aorta, which is potentially life-threatening and often occurs in patients with underlying diseases that weaken the aortic wall, e.g., hypertension, atherosclerosis, and AAA. A distinction is made between type A und type B dissection. Type A is a proximal aortic dissection involving the ascending aorta, while type B affects the descending aorta. The IMH is an atypical aortic dissection and characterised by bleeding into the aortic wall without an intimal tear. A PAU is an ulcerative defect of the intima of the aorta, which breaks through the internal membrane into the tunica media. Acute aortic syndromes usually present with sudden onset of symptoms, like devastating chest pain, which can radiate into the back, including the lower back, and mainly affect middle-aged men. Eleven case reports, two case series, ten cohort studies, nine register studies, a chart review study, an interventional study, thirteen narrative and one systematic review have documented acute aortic syndrome concomitant with LBP (Table 10). Wu et al. published a systematic review and meta-analysis which examines various studies on acute aortic syndrome (partly included in Table 10), where the incidence of back pain varies greatly between 10 % and 75% [164]. Most patients present with a sudden onset of acute severe LBP (pain scale: 7/10, [165]). Possible accompanying symptoms are chest discomfort, abdominal pain, nausea/vomiting, and dyspnoea [9, 166–204]. To confirm the diagnosis, imaging studies, such as computed tomography angiography (CTA), are used [167, 170, 189, 194, 205–208].

**Table 10:** Acute aortic syndrome presenting with back pain

**Author / year / country**	**Design / setting**	**Patient(s)**	**Spinal symptoms**	**Extravertebral symptoms**	**Diagnostic confirmation**	**Other information / comment**
Fojtik et al., 2003, USA [166]	CR / ED	male, 48 y	acute LBP (sharp, sudden onset, radiating into the groin)	diaphoresis, dyspnoea, nausea	CT	h/o smoking cigarettes
Hughes et al., 2016, USA [205]	CR / ED	female, 56 y	acute LBP, bilateral lower extremity paraesthesia and paralysis	-	CTA	h/o smoking cigarettes
Itoga et al., 2017, USA [167]	CR / not reported	male, 59 y	acute LBP, left sided pain and paraesthesia, bilateral buttock stabbing discomfort, resting pain in thigh and calf	abdominal discomfort (radiating to the back)	CTA	-
Johnson et al., 2008, USA [165]	CR / ED	male, 49 y	acute LBP (7/10 NRS, intensifying with prolonged periods of sitting still, moderate breathing difficulties)	-	CT	hypertension
Hsu et al., 2008, China [209]	CR / ED	male, 32 y	acute LBP (acute onset developed during the night)	-	CT	-
Sixsmith et al., 2005, USA [169]	CR / ED	male, 27 y	acute LBP (radiating from upper abdominal area)	diffuse abdominal cramping, bloody diarrhoea, frank blood per rectum	autopsy	-
		male, 60 y	acute LBP, severe left sided flank pain	-	autopsy	hypertension
Stäubli et al., 2004, Switzerland [210]	CR / ED	male, 49 y	acute LBP (sudden, severe, later radiating to the abdomen)	pale, sweating	spiral CT, MRI	h/o smoking cigarettes
Takahashi et al., 2017, Japan [170]	CR / hospital	male, 73 y	acute LBP (severe)	chest pain	CTA	hypertension
Morris-Stiff et al., 2008, UK [211]	CR / ambulatory care	male, 47 y	acute LBP (acute onset, commenced during coitus, radiating down the right leg, increased, claudication-type pain)	-	CT, TTE	hypertension and hyperlipidaemia
Furui et al., 2012, Japan [199]	CR / hospital	male, 83 y	acute back pain (no localisation reported)	chest pain	CT	-
Ahmed et al., 2012, UK [206]	CR / ED	male, 45 y	acute LBP, weakness in lower limbs	collapse	CTA	h/o smoking cigarettes, family history of ischaemic heart disease
Asouhidou et al., 2009, Greece [175]	CS / ED	49 (16.3% female), mean age: 54.8 y	back pain (no duration or localisation reported) with chest pain (8.1%), paralysis of lower extremities (4.1%), hemiparesis (2%)	only chest pain (36.7%), chest pain with neurological deficit (12.2%), chest pain with syncope (8.1%), chest pain with CHF (6.1%), CHF (10.2%), syncope (6.1%), intubated (4.1%), syncope with pulselessness of the lower extremities (2%)	-	-
Nathan et al., 2012, USA [202]	CS / hospital	315 (39.8% female), mean age: 73.2 y	back pain (or chest pain) (11%,) (no duration or localisation reported)	chest pain	-	-
Kalko et al., 2008, Turkey [171]	CHS / hospital	8 (12.5% female), mean age: 62.5 y	severe back pain (and abdominal pain) (57%) (no duration or localisation reported), acute ischaemia with paraplegia of the lower limb (14.3%)	abdominal pain, haemodynamic collapse, and shock (28.6%)	-	-
Hsu et al., 2005, Taiwan [172]	CHS / hospital	107 (23.4% female), mean age: 58.4 y	back pain (or chest pain) (91.6%) (no duration or localisation reported)	intramural haematoma (12.1%), leg ischaemia (8.4%), hypotension / shock (0.9%)	-	-
Li et al., 2012, China [173]	CHS / hospital	1812 (total: 22.5% female), mean age: 51.1 y	acute back pain (69.1%) (no localisation reported), other neurological deficits (1.5%)	pain (88.1%), abrupt onset (70.3%), anterior chest pain (69.4%), abdominal pain (12.3%), migrating pain (8.7%), leg pain (1.7%), pulse deficit (14.1%), aortic regurgitation murmur (9.2%), syncope (5.7%), shock (5.3%), stroke (5.0%), heart failure (4.1%), systolic blood pressure (143.2 +/- 24.4), diastolic blood pressure (81.8 +/- 13.8)	-	-
Xu et al., 2006, China [212]	CHS / hospital	63 (6.3% female), mean age: 50.4 y	back pain (100%) (no duration or localisation reported)	shock (3.2%), hoarseness (1.6%)	-	-
Falconi et al., 2005, Argentina [174]	CHS / hospital	AD: 76 (34% female), mean age: 69 y, IMH: 27 (30% female), mean age: 71 y	acute back pain (72%) (no localisation reported)	abrupt onset of pain (85%), chest or back pain (25%)	-	-
Sen et al., 2020, USA, Switzerland [200]	CHS / hospital	14 (29% female), median age: 73 y (range: 44-90 y)	acute back pain (7%) (no localisation reported)	abdominal pain (14%), hypotension (7%)	-	-
Ho et al., 2011, China (Classic AD) [201]	CHS / hospital	classic AD: 56 (32.1% female), mean age: 60.5 y	back pain (64.3%) (no duration or localisation reported)	chest pain (75%), abdominal pain (23.2%), stroke (21.4%)	-	-
		IMH: 34 (47.1% female), mean age: 69.7 y	back pain (64.7%) (no duration or localisation reported)	chest pain (70.6%), abdominal pain (26.5%), stroke (0%)	-	-
Jansen Klomp et al., 2016, Netherlands [184]	CHS / hospital	200 (39% female), mean age: 64 y	comparison between part of first DDx (fDDx) and not part of first DDx (nfDDx): back pain: (fDDx: 56.1%, nfDDx: 31%) (no duration or localisation reported), focal neurological deficit (fDDx: 15.8%, nfDDx: 9.9%)	chest pain (64.7% vs 61.3%), abdominal pain (23.7% vs 24.0%), TLOC (19.5% vs 10.7%), coma (12.1% vs 10.7%); signs: any pulse deficit (27.7% vs 9.9%), median heart rate (73 vs 78), median blood pressure (120/104 vs 120/102), median haemoglobin (7.8 vs 8.0), median creatinine (104 vs 102)	-	-
Collins et al., 2004, USA [185]	CHS / hospital	617 (100 with previous surgery (w), 517 without previous surgery (wo)), female (overall: 33.1%), mean age overall: 61.1 y	back (or chest pain) (overall: 84.6%, w: 67.4%, wo: 87.9%) (no duration or localisation reported), abrupt onset of back or chest pain (overall: 91%, w: 83.9%, wo: 92%)	migrating pain (overall: 14.8%, w: 8.8%, wo: 15.9%)	-	-
Imamura et al., 2011, Japan [186]	CHS / hospital	98, painless (pl): 16 (44% female, mean age: 71 y), painful (pf): 82 (46% female, mean age: 65 y)	pf: back pain (60%) (no duration or localisation reported), focal neurological deficit (pl: 19%, pf: 2%), weakness of lower extremities (pl: 6%, pf: 12%)	chest pain (71%), abdominal pain (27%), disturbance of consciousness (transient: pl: 25%, pf: 1%; persistent: pl: 44%, pf: 6%), dyspnoea (pl: 6%, pf: 2%), nausea and vomiting (pl: 6%, pf: 7%), abdominal fullness (pl: 6%, pf: 0%), bleeding tendency (pl: 6%, pf: 0%), pyrexia (pl: 0%, pf: 2%), haematemesis (pl: 0%, pf: 1%)	-	-
Januzzi et al., 2004, USA [176]	register study / hospital	1049 (Marfan (marf): 5.1%, non-Marfan (nmarf): 94.9%); marf: 26% female, mean age: 35 y; nmarf: 32% female, mean age: 64 y	LBP (marf: 60%; nmarf: 55%) (no duration reported)	chest pain (marf: 75%, nmarf: 76%), migrating pain (marf: 16%, nmarf: 18%), syncope (marf: 10%, nmarf: 13%), congestive heart failure (marf: 10%, nmarf: 5%), coma / altered consciousness (marf: 6%, nmarf: 10%), systolic blood pressure > 140 mmHg (marf: 27%, nmarf: 44%), diastolic blood pressure > 90 mmHg (marf: 19%, nmarf: 23%), murmur of aortic regurgitation (marf: 46%, nmarf: 32%)	-	-
Nallamothu et al., 2002, USA [177]	register study / hospital	728 (31% female), mean age: 63 y	back pain (or chest pain) (83%) (no duration or localisation reported)	syncope (13%)	-	-
Nienaber et al., 2004, Germany [178]	register study / hospital	1078 (32.1% female), mean age: 62.4 y)	back pain (54.4 %) (no duration or localisation reported), any focal neurological deficits (14.3%), ischaemic peripheral neuropathy (2%)	abrupt onset of pain (87.1%), chest pain (75.5%), migrating pain (17.9%), hypotension / shock / tamponade (19.2%), syncope (13.1%), shock / tamponade (11.5%), coma / altered consciousness (10.3%), shock (10.0%), congestive heart failure (6.6%), cardiovascular accident (6.1%), tamponade (3.6%), mean systolic BP (142 +/- 43), mean diastolic BP (82 +/- 23), any pulse deficit (27.7%)	-	-
Suzuki et al., 2003, Germany [179]	register study / hospital	384 (28.6% female), mean age: 64.4 y	back pain (and / or chest pain) (86%) of abrupt nature (89%) (no duration or localisation reported)	migrating pain (uncommon, 25%), hypertension (69%), pulse deficits (21%), spinal cord ischaemia (3%) hypotension / shock (3%)	-	-
Bossone et al., 2013, Italy [180]	register study / hospital	1354 (36.4% female), mean age: 62.8 y	back pain (59.6%) (no duration or localisation reported)	any pain reported (93.8%), abrupt onset (83%), chest pain (73%), radiating pain (44.1%), abdominal pain (32.6%), migrating pain (7%), syncope (10.3%)	-	-
Evangelista et al., 2005, USA [181]	register study / hospital	classic AD: 952 (30.9% female, mean age: 61.7 y); IMH: 58 (39.7% female, mean age: 68.7 y)	acute back pain (no localisation reported): AD: 54.2%; IMH: 63.8%; abrupt onset described	abrupt onset (AD: 87.3% / IMH: 87.5%), chest pain (AD: 75.6% / IMH: 75.9%), pain rated as worst ever (AD: 20.0% / IMH: 39.6%), abdominal pain (AD: 28.2% / IMH: 31.0%), pain migration (AD: 18.2% / IMH: 15.5%), leg pain (AD: 10.8% / IMH: 1.7%)	-	-
Harris et al., 2012, USA [182]	register study / hospital	type A: AD: 1744 (32.3% female, mean age: 61.4 y), IMH: 64 (42.2% female, mean age: 69.6 y); type B: AD: 651 (33.5% female, mean age: 62.9 y), IMH (34.4% female, mean age 68.6 y)	back pain (no duration or localisation reported) (type A: AD (42.8%), IMH (41.0%); type B: AD (70.3%), IMH (78.7%))	pain severity - severe or worst ever pain (type A: AD (91.6%), IMH (98.1%); type B: AD (93.6%), IMH (94.7%)), abrupt onset of pain (type A: AD (82.6%), IMH (86.7%); type B: AD (87.4%), IMH (82.6%)), chest pain (type A: AD (81.5%), IMH (82.5%); type B: AD (67.4%), IMH (77.3%)), radiating pain (type A: AD (36.3%), IMH (45.9%); type B: AD (44.7%), IMH (35.3%)), abdominal pain (type A: AD (26.0%), IMH (13.1%); type B: AD (43.9%), IMH (36.8%))	-	-
Vagnarelli et al., 2015, Italy [183]	register study / hospital	398 (33.2% female), mean age: 66.7 y	back pain (48.7%) (no duration or localisation reported)	chest pain (65.6%), abdominal pain (27.6%), migratory pain (12.8%), autonomic symptoms (38.9%), dyspnoea (14.6%), shock within 12 hours of admission (14.3%), pain + shock (11.1%), pain + syncope (8.5%), pain + cerebrovascular accident (3.0%), pain + paraplegia (2.5%)	-	-
Wang et al., 2014, China [204]	register study / hospital	Sino-RAD: 1003 (22.2% female), mean age: 51.8 y; IRAD: 464 (34.7% female), mean age: 63.1 y	back pain (no duration or localisation reported) (Sino-RAD: 77%, IRAD: 53.2%)	any pain reported (Sino-RAD: 89.6%, IRAD: 95.5%), abrupt onset (Sino-RAD: 68.5%, IRAD: 84.8%), chest pain (Sino-RAD: 17.3%, IRAD: 72.7%), abdominal pain (Sino-RAD: 12%, IRAD: 29.6%), syncope (Sino-RAD: 2.1%, IRAD: 9.4%), heart failure (Sino-RAD: 0.2%, IRAD: 6.6%)	-	-
Tsai et al., 2009, Taiwan [203]	chart-review study / hospital	18 (16.7% female), mean age: 32 y	LBP (11.1%) (no duration reported)	chest pain and / or tightness (38.9%), chest-back pain (27.8%), epigastric pain (11.1%), abdominal pain (5.6%), lower limb weakness or numbness (11.1%), sudden cardiac arrest (5.6%)	-	-
Li et al., 2010, China [187]	interventional study / hospital	stent graft: 33 (27% female, mean age: 60 y); treated medically: 23 (35% female, mean age: 56 y)	back pain (or chest pain) (100% of all patients) (no localisation or duration reported)	-	-	-
Léon Ayala et al., 2011, Taiwan [188]	NR / -	male (68%) > female (32%)	acute back pain (no localisation reported), sudden onset, neurological deficits: paraplegia	most common symptom: sudden onset of severe chest pain, radiating to neck or shoulders; hypotension and / or shock (type A), hypertension (type B); other findings: fever, diaphoresis, absence of pulse, cerebrovascular manifestations, acute abdominal pain, aortic regurgitation related to cardiac failure, cardiac tamponade, syncope	diagnostic methods: laboratory testing, ECG, TEE / TTE, CT, MRI, aortography, chest x-ray	diagnosis was missed up in 38%; signs: pulse deficit (< 20%), diastolic murmur, jugular venous distention, distant heart sounds, pulsus paradoxus, ostium or coronary artery involved (7%); risk factors: cocaine, pregnancy, iatrogenic trauma
Golledge et al., 2008, USA [189]	NR / -	incidence: 3 / 100000 people per year, age: 61-65 y, male > female	back pain (or chest pain) (85%) (no duration or localisation reported), abrupt onset; any focal neurological deficit (12%)	severe or worst-ever pain (90%), abrupt onset of pain (90%), pain presenting within 6 hrs. of symptom onset (79%), abdominal pain (30%), migrating pain (19%), hypertension at presentation (49%), aortic regurgitation (32%), any pulse deficit (27%), hypotension / shock / tamponade (18%)	diagnostic methods: laboratory testing, ECG, chest radiograph, CT, echocardiography, CTA, MRI	risk factor: hypertension
Khan et al., 2002, USA [190]	NR / -	male > female, mean age: 50-55 y proximal and 60-70 y distal; patients with Marfan syndrome tend to be younger	back pain (53%) (no duration or localisation reported, depending on location of dissection); cerebral ischaemia / stroke (5-10%), spinal cord ischaemia / ischaemic peripheral neuropathies (up to 10%), various spinal cord syndromes	pain (95%) (typically catastrophic / abrupt onset (85%), sharp (64%), ripping or tearing (51%) or knife-like in nature (no percentage reported)), chest pain (73%), abdominal pain (30%), acute cardiac decompensation and shock, hypotension and shock, pericardial effusion, obstruction of the superior vena cava or from cardiac tamponade, neurological deficits (18-30%), syncope (12%), acute GI haemorrhage / acute abdomen / dysphagia; signs: aortic regurgitation (18-50%), diastolic murmur (25%), systolic BP < 100 mmHg (25%), left ventricular regional wall motion abnormalities (10-15%), low coronary perfusion, pulse differential (38%), bruits	diagnostic methods: TEE, CT, MRI, chest radiograph, TTE, aortography, serum smooth muscle myosin heavy chain	predisposing factors: hypertension, aortic disease, direct iatrogenic trauma, cocaine, pregnancy
Mussa et al., 2016, USA [191]	SR / -	patients with AAD: median age: 61 y, 19-50% female; incidence 15/100000 patient-years	back pain (or chest pain) (84.8%) (no duration or localisation reported); stroke (11.3%)	sharp pain (64.4%); weak carotid, brachial or femoral pulse (pulse deficit) (30%), hypotension (> 25%), syncope (13%), regurgitation murmur (31.6%), abdominal pain (type B: 42.7%, type A: 21.6%), IRAD (syncope (33.9%), congestive heart failure (19.7%), painless aortic dissection (6.4%))	diagnostic methods: ECG, chest x-ray, CT / MRI, TEE, serologic biomarkers (e.g., d-dimer)	most common comorbidity: hypertension (45-100%), h/o smoking (20-85%), chronic renal insufficiency (3-79%), COPD (5-36%), stroke / transient ischaemic attack (0-20%);
		patients with IMH	back pain (61.6%) (no duration or localisation reported)	abrupt chest pain (77.9%), hypertension (68-96%)	diagnostic methods: CT / MRI = gold standards	smokers: 18-67%
Nienaber et al., 2002, Germany [192]	NR / -	-	back pain (no duration or localisation reported)	chest pain	-	risk factors: hypertension (85%), coronary artery disease (61%), abdominal or thoracic aneurysm (53%, chronic renal insufficiency (31%), peripheral artery disease (17%), cerebrovascular accidents (12%)
Nienaber et al., 2003, Germany [193]	NR / -	IMH prevalence: 10 – 30 %	instantaneous onset of severe back pain (64%) (no localisation reported); stroke (21%), peripheral ischaemic neuropathy (encountered on occasion)	chest pain (63%), sudden abdominal pain (43%), ischaemic leg (encountered on occasion)	diagnostic methods: physical examination (pulse deficits: <20%, diastolic murmur: 40-50%), chest x-ray, ECG, TTE, TEE, CT, MRI, angiography	risk factors: smoking, dyslipidaemia, cocaine / crack, Marfan syndrome, Ehlers-Danlos-syndrome, bicuspid aortic valve, coarctation, giant cell arteritis, Takayasu arteritis, Behcet’s disease, syphilis, Ormond’s disease, trauma, iatrogenic factors, pregnancy related
Thrumurthy et al., 2011, UK [194]	NR / -	female: 32%	back pain (no localisation or duration reported); neurological deficit	sharp, tearing, stabbing chest pain radiating to neck (type A) or interscapular region (type B), abrupt onset of ripping, tearing, stabbing pain in chest or abdomen, shock	diagnostic methods: CTA, echocardiography, MRA	risk factors: hypertension (40-75%), race (79% white), connective tissue diseases (Marfan syndrome: 15-50% in patients under 40 years), congenital cardiovascular abnormalities, aortic vasculitis disease, cocaine misuse, pregnancy, iatrogenic (5%)
Tsai et al., 2005, USA [195]	NR / -	incidence: 2.6-3.5 cases per 100.000 person-years	back pain (64% type B vs. 47% type A) (no duration or localisation reported)	cataclysmic onset, chest pain (blunt, severe, sometimes radiating); chest pain (79% type A vs. 63% type B), abdominal pain (43% type B vs. 22% type A)	diagnostic methods: ECG, chest x-ray, TTE, TEE, CT, MRI, aortography, coronary angiography	risk factors: smoking, dyslipidaemia, cocaine / crack, Marfan syndrome, Ehlers-Danlos-syndrome, bicuspid aortic valve, coarctation, giant cell arteritis, Takayasu arteritis, Behcet’s disease, syphilis, Ormond’s disease, trauma, iatrogenic factors
Vilacosta et al., 2001, Spain [196]	NR / -	-	acute back pain (no localisation reported)	severely intense, acute, tearing or tearing, throbbing and migratory chest pain = AAS, anterior chest, neck, throat and even jaw pain = ascending aorta; back and abdominal pain = descending aorta	diagnostic methods: laboratory tests (CK, troponin), ECG, chest x-ray, CT, MRI, TEE	risk factors: severe hypertension, disorders of elastic tissue
Vilacosta et al., 2009, Spain [197]	NR / -	-	acute back pain (no localisation reported)	severely intense, acute, tearing or ripping, pulsating and migratory chest pain = AAS; chest pain irradiating to the neck, throat or jaw indicates = ascending aorta; back or abdominal pain: descending aorta	diagnostic methods: laboratory tests, electrocardiography, chest x-ray, CT, MRI, TEE	risk factors: moderate to severe hypertension, disorders of elastic tissue; physical examination: murmur of aortic regurgitation, pulse differentials
Siegal et al., 2006, USA [198]	NR / -	incidence: 5-30 cases per 1 million people per year, 34% female, age: 65 y	back pain (or abdominal or chest pain) (95%) (no localisation or duration reported), severe or worst ever, sharp (64%), tearing, ripping; neurologic deficits	pulse deficits, hypotension, hypertension, end-organ ischaemia; other clinical findings: acute myocardial ischaemia / infarction, pericardial friction rub, syncope, pleural effusion or frank haemothorax, acute renal failure, mesenteric ischaemia	-	patients history: systemic hypertension (72%), atherosclerosis, h/o prior cardiac surgery, aortic aneurysm, collagen diseases, bicuspid aortic valve, aortic coarctation, Turner syndrome, strenuous exercise, large vessel arteritis (giant cell, Takayasu's, syphilis), cocaine and methamphetamine ingestion, third-trimester pregnancy, blunt chest trauma or high-speed deceleration injury, iatrogenic injury; DDx: acute coronary syndrome, pulmonary embolus, pneumothorax, pneumonia, musculoskeletal pain, acute cholecystitis, oesophageal spasm or rupture, acute pancreatitis and acute pericarditis
Lech et al., 2017, USA [207]	NR / -	patients with AD: male predominance (40% female); patients with IMH typically older and more frequently in Asia than in America or Europe; patients with PAU: age >70 y	acute back pain (no localisation reported)	can be missed up to 40%; most common: sudden onset and severe pain located in the chest, abdomen, flank; painless, pain: tearing and ripping, radiating to the back; other specific symptoms: paresis, paraplegia, syncope; other signs: myocardial ischaemia, cardiac tamponade, cardiogenic shock, acute aortic regurgitation, mesenteric ischaemia; hypertension	diagnostic methods: physical examination (pulse deficit, aortic murmur; gastrointestinal bleeding, haematuria, anuria), CT / CTA, ultrasonography, MRI / MRA	causes / risk factors: hypertension (70%), h/o connective tissue disease, h/o atherosclerosis, cigarette use, illicit drug use, dyslipidaemia, h/o blunt trauma, recent aortic manipulation, family h/o aortic disease, inflammatory disorders (autoimmune and infectious), pregnancy
Corvera et al., 2016, USA [208]	NR / -	1/3 female, average age of male: 63 y and female: 67 y	acute back pain (no localisation reported), tearing, ripping severe	acute onset of severe chest pain, tearing or ripping; other symptoms: syncope, neurological deficit including stroke and paraplegia, acute congestive heart failure, myocardial ischaemia, lower extremity ischaemia, abdominal pain and shock	diagnostic methods: physical examination (aortic murmur (44%), pulse deficit (20-30% in type A), hypertension (1/3 in type A); diagnostic: CTA, TTE, TEE, MRI, catheter angiography, no biomarkers	risk factors: hypertension, atherosclerosis, prior cardiac surgery, known aneurysm and Marfan syndrome
Winters et al., 2006, USA [9]	NR / -	male:female = 5:1, advanced age (50-70 years)	descending aorta: more commonly back pain (no duration or localisation reported) with radiation to hip and legs, neurological symptoms (quadriplegia, paraplegia, unilateral paraesthesia)	instantaneous onset of chest pain (maximal at onset, knifelike, ripping or tearing), syncope, abdominal pain, gastrointestinal bleeding, dysphagia, and hoarseness	diagnostic methods: physical examination: hypertension / hypotension, bilateral blood pressure difference; imaging: chest radiography, CT, MRI, TEE	risk factors: chronic hypertension, smoking, hyperlipidaemia, connective tissue syndromes (Marfan syndrome, Ehlers Danlos syndrome), chromosomal disorders (Turner’s syndrome, Noonan’s syndrome), bicuspid aortic valve, coarctation of the aorta, decelerating trauma, inflammatory conditions of the aorta, aortic instrumentation, pregnancy, cocaine use

*AAD* acute aortic dissection*, AAS* acute aortic syndrome, *AD* aortic dissection*, BP* blood pressure*, CHF* congestive heart failure*, CHS* cohort study*, CK* creatine kinase*, CR* case report*, CS* case series*, CT* computed tomography*, CTA* computed tomography angiography*, COPD* chronic obstructive pulmonary disease*, ECG* electrocardiogram, *ED* emergency department*, GI* gastrointestinal*, h/o* history of*, IMH* intramural haematoma*, IRAD* International Registry of Aortic Dissection*, LBP* low back pain*, marf* marfan-group, *MRA* magnetic resonance angiography*, MRI* magnetic resonance imaging*, nmarf* non-marfan group, *NR* narrative review*, NRS* numeric rating scale, *PAU* penetrating aortic ulcer*, pf* painful, *pl* painless, *Sino-RAD* Registry of Aortic Dissection in China*, SR* systematic review*, TLOC* transient loss of consciousness*, TEE* transoesophageal echocardiogram*, TTE* transthoracic echocardiogram,* w* with previous surgery*, wo* without previous surgery*, y* years

#### Fistula

A fistula is an uncommon connection between two structures, such as organs or vessels. A total of twelve case reports, five case series, seven cohort studies, a chart review, and nine narrative reviews describing different fistulas (e.g., aorto-enteric, aorto-caval, aorto-venous) presenting with LBP (Table 11) were found. Middle-aged and elderly men were most commonly affected. The aetiology varies greatly depending on the localisation, for example, aorto-enteric fistulas are often (up to 80% [213]) caused by AAAs. LBP is described as a frequently accompanying symptom of fistulas, ranging from 1.7% [214] to 93% [215] of affected patients. Accompanying symptoms depend on the structures affected and can include abdominal pain or vomiting [118, 120, 213, 216–219]. Neurological symptoms like paraplegia and sensory disorders can also occur, especially when it is an aorto-venous fistula affecting the spine. The diagnosis is made incidentally during imaging studies especially when patients present with marked symptoms. CT is often used initially [118, 120, 216, 217, 219–228], followed by other possible imaging methods such as duplex sonography, MRI, and digital subtraction angiography (DSA). In some cases, surgery was necessary for diagnosis [215, 221, 229].

**Table 11:** Vascular fistulas presenting with back pain

**Author / year / country**	**Design / setting**	**Patient(s)**	**Diagnosis**	**Spinal symptoms**	**Extravertebral symptoms**	**Diagnostic confirmation**	**Other information / comment**
Mehmood et al., 2007, Ireland [216]	CR / ED	male, 59 y	aorto-enteric fistula	acute LBP	vomiting, central abdominal pain, chronic fatigue	CT	-
Ogura et al., 2020, Japan [230]	CR / hospital	male, 73 y	aorto-venous fistula	chronic LBP, numbness in lower extremities, gait disturbance	-	angiography	-
Ozcakir et al., 2008, USA [213]	CR / ED	male, 60 y	aorto-enteric fistula	acute LBP, severe	abdominal pain, vomiting of blood	laparotomy	-
Patelis et al., 2018, Greece [217]	CR / ED	male, 60 y	aorto-caval fistula	acute LBP, sudden onset	abdominal pain	CTA	-
Verma et al., 2015, USA [231]	CR / ED	male, 54 y	aorto-venous fistula	chronic LBP motion dependent, left lower extremity radiculopathy	-	spinal angiogram	-
Kopp et al., 2006, Germany [220]	CR / hospital	male, 68 y	aorto-caval fistula	acute right sided back pain (no localisation reported), acutely developed, persistent	-	CT	-
Nakazawa et al., 2014, Japan [229]	CR / ambulatory care	male, 82 y	aorto-caval fistula	back pain (no duration or localisation reported), worsening	-	surgery	-
Takazawa et al., 2001, Japan [221]	CR / hospital	male, 65 y	aorto-caval fistula	acute LBP	nausea, haematuria, oedema, cyanosis, coldness in both lower extremities	CT, surgery	-
Koch et al., 2004, Germany [232]	CR / not reported	female, 46 y	dural arterio-venous fistula of the lumbar spine	acute LBP, radiating to both legs, neck stiffness	frontal and occipital headache, nausea	angiography	-
Oldfield et al., 2002, USA [233]	CR / not reported	male, 64 y	spinal dural arterio-venous fistula	LBP (no duration reported), intermittent, gait disturbance (progressively worsening)	urinary hesitance and constipation	spinal arteriogram	-
Pevec et al., 2010, USA [222]	CR / hospital	male, 84 y	aorto-caval fistula	acute back pain (no localisation reported)	ashen, anxious	CT	-
Siepe et al., 2009, Germany [223]	CR / hospital	male, 66 y	aorto-caval fistula	back pain (no duration or localisation reported), severe, incomplete paraplegia	shock developing during neurological examination	CT	-
Kotsikoris et al., 2012, Greece [215]	CS / hospital	14 (sex and age not clearly reported)	aorto-venous fistula	LBP (92.8%) (no duration reported)	abdominal tenderness (78.6%), palpitation (57.1%), dyspnoea (42.8%), haemorrhagic shock (28.6%), congestive heart failure (21.4%)	-	-
Cinara et al., 2005, Serbia [234]	CS / hospital	26 (7.7% female), mean age: 65.3 y	aorto-caval fistula	LBP (92.3%) with palpable abdominal aortic aneurysm	haemorrhagic shock at admission (65.3%), congestive heart failure (11.5%), dyspnoea (11.5%), palpitation (23.1%), collapse (7.0%), leg oedema (50%) (secondary to deep vein thrombosis (3.8%)), anuria (7.0%), haematuria (7%), scrotal oedema or haematoma (23.0%), haematemesis (3.8%)	-	-
Kiyosue et al., 2017, Japan [214]	CS / hospital	168 (dural: 17.6% female, mean age: 64.4 y); (epidural: 27% female, mean age: 66.6 y)	spinal dural and extradural arterio-venous fistula	back pain (dural: 3.7%, epidural: 1.7%) (no duration or localisation reported); dural: myelopathy (most frequently: 97.2%), radiculopathy (4.6%), subarachnoid haemorrhage (0.9%), intramedullary haemorrhage (0.9%); epidural: myelopathy (most frequent, 91.5%), radiculopathy (11.9%)	-	-	-
Muralidharan et al., 2011, USA [235]	CS / hospital	153 (22.2% female), mean age: (female: 62 y, male: 64.3 y)	spinal dural arterio-venous fistula	back pain (no duration or localisation reported), slow progression or stepwise worsening deficits, bilateral lower extremity weakness (82%), unilateral lower extremity weakness (16%), bilateral upper extremity weakness (2%), bilateral lower extremity numbness (39%), unilateral lower extremity numbness (17%), bilateral lower extremity dysesthesias / paraesthesia (29%), unilateral lower extremity dysesthesias / paraesthesia (15%), back pain +/- radiation to lower extremities (77%), pain confined to one or both extremities (23%), sphincter disturbances (neurogenic bowel or bladder, 3.9%), dyspnoea (0.6%)	-	-	-
Davidovic et al., 2008, Serbia [236]	CS / hospital	primary aorto-duodenal fistula: 5 (20% female, mean age; 63 y); aortocaval fistula: 25 (8% female, mean age: 65.6 y)	aorto-duodenal or aorto-caval fistula	ADF: LBP (80%) (no duration reported), ACF: LBP (100%) (no duration reported)	ADF: lower gastrointestinal bleeding, haemorrhagic shock (60%), ACF: haemorrhagic shock (72%), dyspnoea (16%), CHF (12%), severe leg swelling (56%), anuria (12%), haematuria (8%), scrotal oedema or haematoma (24%)	-	-
Davidovic et al., 2011, Serbia [237]	CHS / hospital	50 (8% female) mean age: 67 y	aorto-caval fistula	LBP (or abdominal pain) (84%) (no duration reported)	pulsatile abdominal mass (100%), abdominal bruit (50%), shock (48%), lower limb oedema (44%), haematuria (32%), congestive heart failure (26%), oliguria (24%), scrotal oedema or haematoma (20%), unconsciousness (10%), anuria (8%), lower limb deep vein thrombosis (8%)	-	-
Davidovic et al., 2002, Yugoslavia [238]	CHS / hospital	16 (6.2% female), mean age: 61.3 y	aorto-caval fistula	LBP (81.25%) and pulsating abdominal mass were predominant symptoms	haemorrhagic shock (68.75%), congestive heart failure (18.7%), dyspnoea and palpitation (37.5%), extensive oedema of lower extremities (31.2%), haematuria (12.5%), scrotal oedema (25%)	-	-
Narvid et al., 2008, USA [239]	CHS / hospital	63 (21% female), mean age: 62 y	spinal dural arterio-venous fistula	back pain (institutional: 24%, review: 22%) (no duration or localisation reported), lower extremity weakness (institutional: 52%, review: 48%), lower extremity paraesthesia (institutional: 30%, review: 35%)	urinary symptoms (institutional: 6%, review: 7%)	-	-
Rangel-Castilla et al., 2011, USA [240]	CHS / not reported	7 (29% female), mean age: not reported	spinal extradural arterio-venous fistula	back pain (28.6%) (no duration or localisation reported), unilateral or bilateral lower-extremity radiculopathy (71.5%); bowel and bladder dysfunction (57.1%), foot drop (14.3%)	-	-	-
Gemmete et al., 2013, USA [241]	CHS / hospital	33 (21.2% female), mean age: 64.6 y	spinal dural arterio-venous fistula	nonspecific back pain (52%) (no duration or localisation reported), lower extremity weakness (88%), patchy dermatomal symptoms (76%)	urinary symptoms (27%)	-	-
Maeda et al., 2007, Japan [218]	CHS / hospital	5 (all male), mean age: 63 y	aorto-caval or aorto-iliacal fistula	acute LBP (or abdominal pain) (80%)	chest pain associated with angina pectoris (20%); classic triad of abdominal or back pain, pulsatile mass, and abdominal bruit (40%), leg oedema (20%), hypovolaemic shock (60%), congestive heart failure (80%), renal dysfunction (80%)	-	-
Van Dijk et al., 2002, Canada [242]	CHS / hospital	49 (20.4% female), mean age: 63 y)	dural arteriovenous fistulas	back pain (no duration or localisation reported) (39%); leg weakness or paraparesis (96%), sensory numbness or paraesthesia (90%)	urinary incontinence or retention (82%), bowel problems (65%)	-	-
Ruiz-Juretschke et al., 2011, Spain [243]	chart review study / hospital	19 (5.3% female), mean age: 62 y	spinal dural arterio-venous fistula	lumbar or radicular pain (47.3%) (no duration reported), spastic paresis (89.5%), urinary sphincter dysfunction (78.9%), hypoesthesia (68.4%), clinical course followed: progressive myelopathy and / or radiculopathy (89.5%)	complete urinary incontinence (26%), erectile dysfunction (32%)	-	-
Assar et al., 2009, USA [118]	NR / -	-	aorto-caval fistula	back pain (no duration or localisation reported)	abdominal pain, pulsatile abdominal mass, continuous bruit (50-90%), high output heart failure, dyspnoea, tachycardia, wide pulse pressure, cyanosis, lower limb oedema, angina, palpitations, hypotension, fever, oliguria, haematuria, pulsatile peripheral veins, diminished lower limb pulses	diagnostic methods: CT	3-4% with ruptured AAA, diagnosis was missed in 50%
Brightwell et al., 2012, UK [219]	NR / -	-	aorto-caval fistula	LBP (no duration reported)	dyspnoea, increased jugular venous pressure, pulmonary oedema, widened pulse pressure, abdominal bruit / thrill, palpable abdominal aneurysm, oliguria, leg oedema with / without cyanosis, pulsating varicose veins, haematuria and rectal bleeding, shock, abdominal pain, chest pain, scrotal oedema, tenesmus, priapism, and poor peripheral pulses	diagnostic methods: physical examination (pulsatile abdominal mass, abdominal bruit or thrill) CT, doppler ultrasound, arteriography / venography, angiography; diagnostic confirmation: CT, MRI, radioisotope studies	80% result of a spontaneous rupture
Koch et al., 2006, Germany [224]	NR / -	mean age: 60 y	spinal dural arterio-venous fistula	LBP (no duration reported); early complaints (paraesthesia, sensory and gait disturbance), paraparesis, tetraparesis, flaccid paresis	impairment of micturition (anuresis or urinary incontinence, any type of bowel or bladder dysfunction (60-90%), pain (25-50% and more)	diagnostic methods: LP, MRI, DSA, aortography, CTA	70% of all spinal arteriovenous malformation, 15% acute to subacute onset, delay 10 to 15 months
Marcus et al., 2013, USA [225]	NR / -	male predominance (80%), age: 50-60 y	spinal dural arterio-venous fistula	LBP (no duration reported), lower extremity weakness, gait disturbances (96%), sensory symptoms, bowel / bladder disturbances, radiculopathies, neurological deficits secondary to progressive myelopathy	sexual dysfunction, bowel and bladder incontinence and urinary retention (urinary dysfunction = 59%)	diagnostic methods: MRI, CTA / MRA, spinal angiography	70% of all spinal arteriovenous malformation, relatively underdiagnosed; predisposing factors: thrombosis of the extradural spinal veins and trauma, usually progressive with an insidious development of disability, delay: 12-44 months
Krings et al., 2004, Germany [227]	NR / -	spinal dural arterio-venous fistulae = 70% of all AV shunts of the spine, male > female, age: 40-60 y	dural arterio-venous fistula	back pain (no duration or localisation reported), may irradiate to the lower legs, congestive myelopathy, hypo- and paraesthesia, paraparesis, impotence, sphincter disturbances	-	diagnostic methods: MRI, MRA, spinal angiography	slowly progressive, DDx: polyneuropathy, glioma, degenerative disc disease syringohydromyelia, inflammatory lesion or spinal ischaemia
Da Costa et al., 2009, Canada[244]	NR / -	3rd decade of life, no sex predominance	spinal cord arterio-venous malformation	acute, severe back pain (no localisation reported), sudden onset of new or worsening pre-existing neurological deficits, motor and sensory symptoms, sexual, bladder and bowel dysfunction, weakness	haemorrhage (50%), bruit	diagnostic methods: MRI, angiography	-
Klopper et al., 2009, USA [228]	NR / -	20% female, 80% > 40 y	spinal dural arterio-venous fistula	nonspecific, back pain (50% with radicular pain); radicular pain (50% with back pain), lower extremity weakness (33%), impaired sensation or paraesthesia (33%)	disturbance of micturition, defecation, or sexual function (10%)	diagnostic methods: laparotomy, myelography, CT, MRI, spinal angiography, MRA / CTA	mostly: lower thoracic or thoracolumbar region
Krings et al., 2009, Canada [226]	NR / -	male > female, mean age 35-60 y	spinal dural arterio-venous fistula	LBP (no duration reported), difficulty in climbing stairs, gait disturbances, sensory symptoms	bowel and bladder incontinence, erectile dysfunction, urinary retention	diagnostic methods: MRI / MRA, DSA, CTA, selective angiography	70% of AVM, DDx: polyneuropathy, tumour, degenerative disc disease, prostatic hypertrophy (urinary retention), another type of spinal vascular malformation, glioma, inflammatory lesion, spinal ischaemia
Thron et al., 2001, Germany [245]	NR / -	5-10 cases/ million people/ year, mean age: 60 y, male > female = 5:1	spinal dural arterio-venous fistula	back pain (no duration or localisation reported); sensory disturbances with numbness and thermodysaesthesia	non-specific, buckling of the legs, less common pain in the muscles or legs, erectile dysfunction, sphincter disorders	diagnostic confirmation: MRI, myelography, LP, selective spinal angiography	DDx: polyneuropathy, spinal stenosis, disc protrusion, glioma, syringomyelia, spinal infarction

*AAA* abdominal aortic aneurysm, *ACF* aorto-caval fistula*, ADF* aorto-duodenal fistula*, AV* arterio-venous*, AVM* arterio-venous malformation*, CHF* congestive heart failure*, CHS* cohort study*, CR* case report*, CT* computed tomography*, CTA* computed tomography angiography*, CS* case series*, DSA* digital subtraction angiography, *ED* emergency department*, LBP* low back pain, *LP* lumbar puncture*, MRI* magnetic resonance imaging*, NR* narrative review*, MRA* magnetic resonance angiography*, y* years

#### Miscellaneous

Six case reports, one cohort study and two narrative reviews reporting miscellaneous vascular disorders presenting with LBP, e.g., aortic thrombosis or coronary artery dissection, were found (Table 12). These disorders commonly present with acute LBP [168, 246–250] with accompanying symptoms like chest discomfort and weakness of lower extremities [227, 247, 251, 252].

**Table 12:** Miscellaneous vascular disorders presenting with back pain

**Author / year / country**	**Design / setting**	**Patient(s)**	**Diagnosis**	**Spinal symptoms**	**Extravertebral symptoms**	**Diagnostic confirmation**	**Other information / comment**
Calderon et al., 2011, Spain [246]	CR / ED	male, 45 y	floating thrombus in aorta	acute LBP, sudden onset	syncope	TEE	-
Triantafyllo-poulos et al., 2011, Greece [247]	CR / ED	male, 56 y	infrarenal aortic thrombus	acute LBP, sudden onset, weakness of lower extremities	-	CTA	-
Cataldo et al., 2019, Italy [248]	CR / ED	male, 48 y	renal artery dissection in Ehlers Danlos syndrome	acute LBP (sudden, excruciating after sexual intercourse)	-	genetic assessment and CTA	-
Marshman et al., 2007, UK [251]	CR / not reported	male, 62 y	lumbar extradural arteriovenous malformation	chronic LBP (exercise-related, progressive), bilateral sciatica, bilateral leg weakness, altered sensation in L4-S1 dermatomes; walking distance limited to 10-20 yards, both feet felt cold dusky and swollen; no sphincteric disturbances	-	CT	-
Suntharalingam et al., 2014, Germany [249]	CR / hospital	female, 57 y	arterio-venous malformation	temporary back pain (no localisation reported), hypaesthesia in digiti two to four of her left foot	tumour in her lower ankle	MRI	-
Korkut et al., 2020, Turkey [168]	CR / not reported	male, 25 y	renal artery dissection	acute LBP, left-sided flank pain	colic, nausea, and vomiting	CTA	h/o cigarette smoking
Luong et al., 2017, Canada [252]	CHS / not reported	196 (90.8% female), mean age: 52.2 y	coronary artery dissection	LBP (13.8%) (no duration reported)	chest discomfort (96%), radiation to arm (52.0%), nausea or vomiting (23.5%), radiation to neck (22.1%), diaphoresis (21.4%), dyspnoea (19.5%), dizziness (8.7%), ventricular tachycardia (7.1%), fatigue (5.1%), headache (1.5%), cardiac arrest (1.0%), syncope (0.5%)	-	-
Heldner et al., 2012, Switzerland [250]	NR / -	-	acute spinal cord ischaemia syndrome	back pain (no duration or localisation reported); complete or incomplete myelopathy, para- or quadriparesis, loss of sensation (especially pain and temperature), loss of bladder function	-	-	rare condition
		male > female	spinal cord haemorrhage	acute back pain (no localisation reported); neck pain, (intense, knife-like, often show a radicular component), meningeal irritation with headache, neck stiffness, disturbance of consciousness and epileptic seizures	-	-	rare, causes: trauma, anticoagulation, hereditary or acquired bleeding disorders, bleeding from spinal vascular malformation, spinal artery aneurysms, primary spinal cord tumours; could be intramedullary, subarachnoid, subdural or epidural
Krings et al., 2004, Germany [227]	NR / -	fistulous AVM become symptomatic in young patients	spinal cord arterio-venous malformation	back pain (no duration or localisation reported), hypo- or paraesthesia, weakness and diffuse back and muscle pain; sensorimotor symptoms (slowly or acutely)	acute haemorrhage	diagnostic methods: MRI, spinal angiography, spinal MRA	20-30% of all spinal vascular shunts, glomerular AVMs can become symptomatic by venous congestion alone, DDx: spinal haemangioblastoma, perimedullary fistulous AVMs type I, cavernoma

*AVM* arteriovenous malformation, *CHS* cohort study*, CR* case report, *CT* computed tomography, *CTA* computed tomography angiography*, ED* emergency department, *h/o* history of, *LBP* low back pain*, MRA* magnetic resonance angiography*, MRI* magnetic resonance imaging, *NR* narrative review, *TEE* transoesophageal echography*, y years*

### Vascular diseases – venous

Pathologies within the venous system, such as deep venous thrombosis (DVT) involving the lower extremities or the inferior vena cava (IVC) [253], may present with LBP. Numerous case reports were found, where acute LBP formed a component of the clinical manifestation, frequently co-occurring with symptoms suggestive of DVT like leg swelling and oedema (Table 13). Some patients exhibited a history of DVT or factors that predispose them to venous thrombosis. Within many of the documented case reports, there was an additional observation of stenosis, aplasia, and hypoplasia affecting the IVC. Malformation of the IVC can contribute to LBP through two distinct mechanisms. Firstly, they directly elevate the risk of DVT. Secondly, they might induce engorgement (varicosis) of the epi- and intradural veins surrounding the spinal cord, thereby causing neural compression that results in radicular symptoms even in the absence of a DVT. Despite the identification of a compressing mass prior to surgery in patients with radicular symptoms, the definitive diagnosis of thrombosed or non-thrombosed spinal varices exerting pressure on nerve roots was established intraoperatively. It is estimated that between 1-4% of radicular symptoms are due to vascular compression [254, 255]. Most patients experienced clinical improvement after surgical decompression confirming the cause-and-effect relationship with LBP.

**Table 13:** Venous diseases associated with low back pain

**Author / year / country**	**Design / setting**	**Diagnosis**	**Patient(s)**	**Spinal symptoms**	**Extraspinal symptoms, comorbidity**	**Differential diagnosis**	**Diagnostic confirmation**
***Deep venous thrombosis with and without venous malformation***							
Bozkurt et al., 2006, Turkey [256]	CR / ED	IVC stenosis associated with Budd-Chiari Syndrome	female, 27 y	chronic LBP	leg pain	-	abdominal sonography
Nowak et al., 2008, Germany [257]	CR / neurology department	bilateral iliac vein thrombosis and congenital IVC aplasia	male, 18 y	chronic LBP	leg pain, superficial venous engorgement	disc herniation	MRI (absence of vena cava)
Dudeck et al., 2007, Germany [258]	CR / ED	DVT, IVC agenesis	male, 26 y	subacute LBP	leg pain	retroperitoneal mass, lymphoma	CT
Vasco et al., 2009, Spain [259]	CR / ED	DVT, IVC agenesis	female, 36 y	acute LBP	leg oedema	-	vascular sonography
Kogias et al., 2011, Germany [31]	CR / ED	DVT, IVC hypoplasia	male, 21 y	acute LBP	leg pain	lumbar disc herniation	CT, MRI
Aday et al., 2016, USA [260]	CR / ED	iliofemoral DVT	female, 29 y	acute LBP	difficulties to walk, leg oedema	-	vascular sonography
Di Nicolo et al., 2016, Italy [24]	CR / not reported	IVC malformation / duplication	male, 33 y	acute, mechanical LBP	haematuria / h/o urolithiasis	urolithiasis	abdominal sonography
Williams et al., 2017, USA [261]	CR / not reported	IVC atresia complicated by extensive DVT	male, 27 y	acute exacerbation of chronic LBP	right groin numbness, oedema of both legs with tenderness to palpation over the medial aspect of the left thigh	-	MRI
Langer et al., 2017, Portugal [262]	CR / ED	DVT, IVC hypoplasia	male, 30 y	acute LBP	leg pain, difficulties to walk, leg oedema	-	vascular sonography, CT
Adachi et al., 2018, Japan [263]	CR / referred to specialist care	DVT, IVC hypoplasia	male, 32 y	acute LBP	pain radiating to both thighs, leg oedema	-	vascular sonography
Ikeda et al., 2019, Japan [264]	CR / ED	pulmonary embolism	male, 45 y	acute LBP	chest pain	-	CT
Umar et al., 2019, USA [265]	CR / ED	IVC stenosis associated with Budd-Chiari Syndrome	male, 47 y	acute LBP and unresponsive to NSAIDs	developed cyanosis of both legs, loss of sensation, and the absence of distal pulses shortly after admission	-	vascular sonography
***Epi- or intradural varicosis leading to radicular symptoms***							
Genevay et al., 2002, France [266]	CR / not reported	epidural varicosis	female, 67 y	chronic LBP unresponsive to treatment	leg pain, no other neurologic symptoms	disc herniation	intraoperatively
			female, 70 y	acute LBP	leg pain, bilateral paresis		
Hammer et al. 2003, UK [491]	CS / hospital	epidural varicosis	6 patients, 4 female (age: 38, 29, 50, 30 y), 2 male (20, 35 y)	acute or chronic LBP	leg pain	disc herniation	MRI, intraoperatively
Moonis et al., 2003, USA [267]	CR / not reported	intradural varicosis	male, 82 y	chronic LBP	leg pain, no other neurological symptoms	intradural nerve sheath tumour	MRI, intraoperatively
Paksoy et al., 2004, Turkey [268]	CS / not reported	epidural venous plexus enlargement with IVC obstruction or occlusion	13 patients, 69.2% female, age: 23-40 y	LBP (no duration reported)	leg pain, h/o Behcet’s disease	-	MRI
Pennekamp et al., 2007, Germany [269]	CR / ED	epidural venous varicosis	female, 40 y	acute LBP	leg pain, paresis	disc herniation	MRI
Aoyama et al., 2008, Japan [255]	CR / neuro-surgery de-partment	epidural venous varicosis	male, 33 y	subacute LBP	leg pain, loss of patellar tendon reflex	cystic nerve sheath tumour	MRI (lumbar mass)
Paldor et al., 2010, Israel [270]	CR / neuro-surgery de-partment	intradural lumbar varicosis	male, 55 y	LBP (no duration reported)	pain radiating to the buttocks and right thigh, neurological examination was normal	intradural tumour, suspected to represent a schwannoma or ependymoma	MRI with breath hold and Valsalva
Lee et al., 2011, Korea [271]	CR / not reported	calcified epidural varicosis	female, 72 y	chronic LBP	pain radiating into the left buttock, weakness in her left hip flexor with numbness in the left L1 and L2 dermatomes	disc herniation	CT (calcified lesion in the left epidural space)
Pacult et al., 2018, USA [272]	CR / ED	gluteal varicosis	female, 76 y	chronic LBP	leg numbness and weakness	disc herniation	MRI
Im et al., 2018, Korea [273]	CR / neuro-surgery de-partment	epidural varicosis	female, 36 y	acute LBP	leg pain, paresis	facet joint synovial cyst	MRI, CT-angiography
Hallan et al., 2020, USA [274]	CR / referred to ED	dilated epidural venous plexus	female, 50 y	acute LBP	bilateral lower extremity weakness, systemic Lupus	-	MRI (vascular mass)
			female, 60 y	chronic progressive LBP	leg pain, h/o DVT	-	-

*CR* case report, *CT* computed tomography, *CS* case series, *DVT* deep venous thrombosis, *ED* emergency department, *h/o* history of, *IVC* inferior vena cava*, LBP* low back pain*, MRI* magnetic resonance imaging*, NSAIDs* non-steroid anti-inflammatory drugs,* y* years

Ikeda et al. [264], documented a case of pulmonary embolism originating from an IVC calcification, which manifested with symptoms of back and chest pain. However, the specific anatomical site of the back pain was not provided in their report.

Ovarian vein syndrome (OVS) is a rare condition caused by varicose, dilated ovarian veins inducing chronic ureteral obstruction. In one case series, the majority (12/13) of women reported back pain; however, the clinical presentation was dominated by urological symptoms [275].

A completely different venous pathology was described by Kalender et al. [276], who reported the rupture of an iliac vein leading to a retroperitoneal haematoma. The patient presented with LBP associated with abdominal pain [276].

### Paraspinal haematoma

Spinal subdural, epidural, or subarachnoid haematomas are infrequent occurrences that can lead to acute spinal cord compression, giving rise to symptoms like radicular syndromes, paraparesis, or cauda equina syndrome (characterised by urinary and faecal incontinence or constipation). A total of 17 case reports or case series depicting paraspinal haematoma-induced LBP were identified (Table 14). They are mostly caused by trauma, lumbar puncture, and spinal surgery, which are beyond the scope of this review. However, they can also manifest spontaneously in individuals with coagulating disorders, oral anticoagulation, underlying vascular malformations (e.g., aneurysms or arteriovenous fistulas), neoplasms, and other vulnerabilities of the vessel walls. It can be assumed that a diagnosis is seldom missed due to the severity of symptoms, which usually prompt the utilisation of advanced imaging techniques that ultimately facilitate accurate diagnosis.

**Table 14:** Case reports or case series of paraspinal haematoma presenting as back pain

**Author / country / year**	**Setting**	**Diagnosis**	**Patient(s)**	**Spinal symptoms**	**Extraspinal symptoms / comorbidity**	**Differential diagnosis**	**Diagnostic confirmation**
***Subdural haematoma***							
Vermeulen et al., 2015, Belgium [277]	hospital	spinal subdural and epidural haematomas and ruptured aneurysm with retroperitoneal haematoma	male, 61 y	acute LBP, acute paraparesis, paraesthesia	oral anticoagulation	-	MRI
Castillo et al., 2015, USA [278]	hospital	spontaneous spinal subdural haematoma	male, 69 y	sudden severe LBP and paraparesis	oral anticoagulation for atrial fibrillation	initially misdiagnosed as transverse myelitis	MRI
McHaourab et al., 2019, UK [279]	ED and primary care	spontaneous spinal subdural haematoma	male, 68 y	acute LBP with progression of paraparesis over several days	urinary and faecal retention	initially misdiagnosed as syringomyelia	MRI
Joubert et al., 2019, France [280]	not reported	spontaneous spinal subdural haematoma	female, 82 y	acute LBP, paraparesis	urinary and faecal retention	-	MRI
Yokota et al., 2020, Japan [281]	hospital	acute spinal subdural haematoma	male, 59 y and review of 37 case reports	severe acute LBP	urinary and faecal incontinence	-	MRI
***Epidural haematoma***							
Braun et al., 2007, Spain [282]	not reported	spinal subdural and epidural haematomas	7 patients (4 with non-lumbar pain), 3 patients: female, 87 y, female, 22 y, male 43)	back pain and paraparesis or paraplegia	1x on oral anticoagulation, 1x after epidural catheter	-	MRI (location of lesion thoracic possible)
Baek et al., 2008, Korea [283]	not reported	spontaneous spinal epidural haematoma	2 patients: male, 64 y female, 69 y	acute LBP and paraparesis	one on aspirin	-	MRI
DeSouza et al., 2014, UK [284]	ED	spontaneous spinal epidural haematoma secondary to pseudogout	male, 75 y	sudden onset back pain (no localisation reported) and paraparesis	-	-	MRI
Matsui et al., 2014, Japan [285]	hospital	chronic spontaneous spinal epidural haematoma	male, 74 y	chronic LBP, leg pain	hypertension	-	MRI
Goyal et al., 2016, India [286]	hospital	spontaneous spinal epidural haematomas	male, 54 y	sudden onset of LBP, paraparesis, paraesthesia	oral anticoagulation	-	MRI
Ismail et al., 2017, Libanon [287]	ED	spontaneous spinal epidural haematomas	male, 72 y	acute mechanical LBP	oral anticoagulation	-	MRI
***Subarachnoid haematoma***							
Massand et al., 2005, US [288]	not reported	spinal artery aneurysm rupture and subarachnoid haemorrhage	4 patients	all acute LBP	not reported	-	CT, MRI
			male, 30 y	paraparesis	not reported		
			male, 69 y	radicular symptoms	stroke		
			male, 54 y	radicular symptoms	none		
			male, 73 y	paraparesis	not reported		
Toi et al., 2011, Japan [289]	hospital	subarachnoid haemorrhage associated with paraspinal arteriovenous fistula	male, 60 y	sudden onset LBP and paraparesis	constipation and faecal retention	-	MRI
Steele et al., 2019, UK [290]	ED	vertebral artery dissection and subarachnoid haemorrhage	male, 55 y	LBP (no duration reported), paraesthesia	constipation and faecal retention	-	MRI
***Other locations***							
Yamasaki et al., 2005, Japan [291]	hospital	haematoma in the psoas muscle	male, 53 y	sudden LBP	abnormal chest shadow	-	CT, MRI
Fukuda et al., 2007, Japan [292]	not reported	juxta-facet haematoma	male, 67 y	chronic LBP, radicular pain, paraesthesia	-	-	MRI
Taghipour et al., 2008, Iran [293]	not reported	intradural nerve root haematoma	male, 32 y	severe acute LBP radiating into both legs	-	-	MRI

*CT* computed tomography, *ED* emergency department, *LBP* low back pain*, MRI* magnetic resonance imaging*, y* years

### Chronic periaortitis

#### Retroperitoneal fibrosis

Chronic periaortitis is a term used to describe a group of rare inflammatory diseases, such as retroperitoneal fibrosis (RPF) and inflammatory abdominal aortic aneurysm (IAAA). RPF is characterised by benign proliferation of fibrotic tissue in the retroperitoneal space, which can result in compression of the aorta, sometimes called periaortitis, and the ureters. It is nowadays classified as immunoglobulin G4 (IgG4)-related autoimmune disorder [294]. Multiple case reports or case series and narrative reviews, six cohort studies, one case-control study, one register study, and one randomised controlled trial related to LBP in the context of retroperitoneal fibrosis were identified (Table 15). Due to the location of the fibrotic tissue, certain characteristic symptoms and complications manifest. The predominant initial presenting symptoms typically include LBP accompanied with abdominal pain, with flank pain occasionally reported [294–308]. RPF predominantly presents with subacute or chronic back pain, with acute LBP being a rare occurrence. The nature of the pain is typically described as dull [303, 309, 310], but can also be colicky, if complications such as unilateral or bilateral ureteral stenosis develop [303]. The occurrence of LBP in the presence of RPF varies greatly between 10% [311] and 100% [312]. Other accompanying symptoms are malaise, fever, anorexia, weight loss, unilateral or bilateral lower limb oedema, and scrotal swelling [27, 294–303, 305–311, 313–352]. The patients in the case reports and case series were mostly men aged 40 to 60 years. Confirmation of the diagnosis primarily relied upon biopsies or imaging studies with a reported diagnostic delay between 7 weeks and 16 months [300, 353].

**Table 15:** Retroperitoneal fibrosis presenting with back pain

**Author / year / country**	**Design / setting**	**Patient(s)**	**Spinal symptoms**	**Extraspinal symptoms**	**Diagnostic confirmation**	**Other information / comment**
Blanc et al., 2007, France [295]	CR / not reported	male, 55 y	subacute LBP	abdominal pain radiating into the testis, fatigue, weight loss	CT (thorax, abdomen, pelvis)	-
Cavalleri et al., 2008, Monaco [354]	CR / not reported	male, 50 y	LBP (no duration reported)	no other symptoms	biopsy	-
		male, 47 y	chronic LBP	no other symptoms	biopsy	-
Doshi et al., 2013, UK [309]	CR / hospital	male, 45 y	subacute / chronic LBP (dull)	lethargy, anorexia, acute deterioration in renal function, left testicular swelling	biopsy	-
Drieskens et al., 2002, Belgium [296]	CR / not reported	female, 41 y	LBP (no duration reported)	radiating to abdomen, sleep disturbance, fatigue, anorexia	biopsy + FDG-PET	-
Nemec et al., 2008, Czech Republic [27]	CR / not reported	male, 59 y	chronic LBP (continuous, blunt in character), radiating to loins, buttock and thighs bilaterally, worsening during walking	weight loss, sore throat with fever, night sweat	CT-guided needle biopsy	-
Paetzold et al., 2013, Austria [297]	CR / ambulatory care	female, 46 y	acute LBP	fatigue, diffuse abdominal pain, skin mycosis	MRI, 18 FDG-PET	-
Reilly et al., 2005, USA [298]	CR / ED	male, 60 y	acute LBP	radiating to lower abdomen and suprapubic region, fatigue, nausea	biopsy	-
Tritschler et al., 2014, Switzerland [313]	CR / hospital	male, 47 y	subacute / chronic LBP	fatigue, loss of appetite, weight loss, subfebrile temperatures	biopsy	-
Yang et al., 2018, China [355]	CR / ED	male, 62 y	acute LBP (persistent, not relieved after taking NSAIDs)	not reported	CT abdomen	-
Zen et al., 2006, Japan [310]	CR / hospital	male, 52 y	LBP (no duration reported) (dull, paraspinal, without tenderness)	low-grade fever (37°C)	CT-guided needle biopsy	-
Brodman et al., 2003, Austria [314]	CR / not reported	male, 62 y	LBP (no duration reported)	lower abdominal pain, weight loss, fatigue	CT, FDG-PET	-
Famularo et al., 2009, Italy [356]	CR / not reported	male, 53 y	back pain (no duration and localisation reported)	arthralgias	CT	-
Young et al., 2008, USA [357]	CR / outpatient clinic -> hospital	male, 67 y	chronic LBP (gradually worsening)	not reported	CT	-
Jois et al., 2004, UK [315]	CR / not reported	male, 67 y	subacute LBP (intermittent, worse at night and disturbing sleep)	weight loss	CT	-
Maritati et al., 2012, Italy [316]	CR / hospital	female, 49 y	back pain (no localisation and duration reported)	malaise	laparoscopic biopsy	-
Vaglio et al., 2008, Italy [317]	CR / hospital	male, 76 y	LBP (no duration reported)	fatigue, comorbidities: surgery for IAAA 1 year ago	CT	-
		female, 54 y	LBP (no duration reported)	hypogastric pain	CT	-
		female, 63 y	LBP (no duration reported)	abdominal pain	MRI	-
Kawamoto et al., 2003, Japan [318]	CR / not reported	female, 40 y	back pain (no duration or localisation reported)	low-grade fever, general fatigue	CT / MRI	-
Oshiro et al., 2005, Japan [319]	CR / hospital	male, 58 y	left sided back pain (no duration or localisation reported), claudication	left lower extremity oedema, left inguinal mass, anaemia, left hydronephrosis	biopsy	-
Wu et al., 2002, USA [358]	CR / not reported	male, 39 y	chronic back pain (no localisation reported)	bilateral hydronephrosis	US	-
Hamano et al., 2002, Japan [359]	CR / hospital	male, 60 y	first and second presentation: back pain (no duration or localisation reported)	first presentation: hydronephrosis left sided; second presentation: epigastric pain, jaundice	first presentation: histological assessment; second presentation: exploratory biopsy	-
Pizzini et al., 2007, Italy [360]	CR / hospital	female, 68 y	LBP (no duration reported)	dyspnoea, palpitations	MRI + laparoscopy / biopsy	-
Zeina et al., 2007, Israel [352]	CR / not reported	male, 53 y	chronic LBP (moderate intensity, unaffected by motion, not relieved by bed rest)	weight loss (4 kg)	CT	-
Al-Hammouri et al., 2019, Jordan [361]	CS / hospital	116 (27% female), mean age: 50.5 y	LBP (79%) (no duration reported)	bilateral ureteral obstruction (58.6%), acute renal failure and uraemic symptoms (27%), unilateral ureteral obstruction (20.6%), no obstruction (17.2%), asymptomatic (13.8%), new onset hypertension (10.3%), anejaculation (3.4%)	-	-
Chiba et al., 2013, Japan [311]	CS / hospital	10 (40% female), mean age: 70.1 y	LBP (10%) (no duration reported)	joint pain (10%), swelling of lacrimal / salivary glands (30%), visual disturbance (20%), fever (10%), dry mouth (10%), oedema of the lower extremities (10%), dyspnoea (10%)	-	associated diseases: AIP, sialadenitis, dacryoadenitis, lymphadenopathy, pulmonary pseudotumor, pituitary pseudotumor
Hadzi-Djokic et al., 2015, Serbia [353]	CS / hospital	15 (27% female), mean age: 56.4 y	LBP (75%) (no duration reported)	unilateral ureteral obstruction (47%), uraemia (26.7%), urinary tract infection (13.3%)	-	diagnostic delay: 15.8 months
Li et al., 2011, China [294]	CS / hospital	61 (21% female), mean age: 55.7 y	LBP (38%) (no duration reported)	abdominal pain (32.8%), loin tenderness (19.7%), abdominal tenderness (11.5%), abdominal mass (8.2%), fever (4.9%), weight loss (4.9%), hypertension (3.3%), abdominal swelling (1.6%), decreased appetite (1.6%), haematuria (1.6%), lower limb oedema (1.6%)	-	mean duration of symptoms: 8 months
van Bommel et al., 2009, Netherlands [299]	CS / hospital	53 (23% female), mean age: 64 y	LBP (60%) (no duration reported), upper leg claudication (11%)	abdominal pain (57%), testicular pain (46%), discomfort (92%), urinary frequency (47%), flank pain (42%), weight loss (40%), constipation (30%), hydrocele (29%), nausea / vomiting (25%), fever / rigors (17%), lower extremity oedema (8%), anejaculation (7%)	-	median duration of symptoms: 6 months
Yachoui et al., 2016, USA [300]	CS / hospital	26 (23% male), median age: 58 y	LBP (29%) (no duration reported)	abdominal pain (58%), flank pain (42%), constitutional (33%), vomiting (17%), constipation (12%), asymptomatic (8%), diarrhoea (4%), arthralgia (4%), lower extremity oedema (4%)	-	diagnostic delay: 7 weeks
Corradi et al., 2007, Italy [322]	CS / hospital	24 (33% female), median age: 56 y	back pain (88%) (no duration or localisation reported)	ureteral involvement (83%), constitutional symptoms (malaise, anorexia, weight loss and fever) (79%), hydrocele and / or varicocele (69%), acute renal failure (54%), constipation (13%), deep vein thrombosis of lower limbs (8%)	-	associated autoimmune disease (33%)
Ilie et al., 2006, UK [323]	CS / not reported	28 (29% female), mean age: 64.1 y	back pain (29%) (no duration or localisation reported), left anterior thigh claudication (4%)	loin pain (40%), groin and testicular pain (18%), abdominal pain (18%), associated high blood pressure (36%), oedema (11%), hydrocele (7%)	-	coexisting: AAA (36%), diabetes (7%), myocardial infarction (11%), venous thrombosis (7%), primary sclerosing cholangitis & small vessel vasculitis & ureteric stone & TCC (7%)
Jansen et al., 2010, The Netherlands [324]	CS / hospital	26 (35% female), mean age: 67 y	back pain (92%) (no duration or localisation reported), claudication (4%)	discomfort (96%), hydrocele / testicular pain (53%), weight loss (50%), pollakiuria (38%), nausea / vomiting (23%), constipation (19%), fever / rigors (4%), lower extremity oedema (4%)	-	decrease in visual PET score correlated with decrease in ESR over time, but not with CRP level or CT-documented mass regression over time
Kermani et al., 2011, USA [326]	CS / hospital	185 (39% female), mean age at diagnosis: 57.6 y	back pain (38%) (no duration or localisation reported), lower extremity claudication (2%)	abdominal pain (40%), flank pain (21%), testicular pain (13%), weight loss (27%), nausea (20%), vomiting (13%), fatigue (13%), new lower extremity oedema (13%), constipation (12%), subjective fever (9%), anorexia (9%), arthralgias (5%), night sweats (4%)	-	-
Nakajo et al., 2007, Japan [327]	CS / PET centre	6 (all male), mean age: 64 y	back pain (33%) (no duration or localisation reported)	abdominal pain (17%), ureteral involvement (50%), general fatigue (17%), anorexia (17%), asymptomatic (17%)	-	-
Van Bommel et al., 2007, The Netherlands [328]	CS / hospital	34 (21% female), mean age: 63 y; 35% idiopathic, 65% secondary	back pain (88%) (no duration or localisation reported), claudication (15%)	discomfort (94%), pollakiuria (42%), weight loss (36%), constipation (24%), hydrocele / testicular pain (23%), fever / rigors (15%), anejaculation (15%)	-	mean duration of symptoms: 6 months
Vega et al., 2009, Chile [329]	CS / not reported	7 (14% female), median age: 64 y	back pain (57%) (no duration or localisation reported), myalgias (14%), leg claudication (14%)	abdominal pain (57%), testicular pain (29%), ureteral colic pain (14%), constitutional symptoms (43%), constipation (29%), deep vein thrombosis of 1 leg (29%), renal insufficiency (29%), hydrocele (14%), renal colic (14%), oedema of lower limbs (14%), varicocele (14%), periumbilical mass (14%), acute renal failure (14%), prolonged fever (14%)	-	-
Zen et al., 2009, Japan [330]	CS / hospital	17 (35% female), mean age: 62 y	back pain (35%), LBP (6%) (no duration reported)	lower abdominal pain (12%), asymptomatic (29%), oedema in lower extremities (29%), fever (6%)	-	42.9% with back ache: IgG4 related
Salvarani et al., 2005, Italy [331]	CS / hospital	7 (43% female), median age: 60 y	back pain (57%) (no duration or localisation reported)	abdominal pain (71%), testicular pain (14%), constitutional symptoms (fatigue, anorexia, weight loss, fever, diffuse myalgias, arthralgias) (57%), ureteral obstructive disease (57%), varicocele (29%), thrombosis (29%), hydrocele (14%), inferior vena cava syndrome (14%)	-	-
Vaglio et al., 2003, Italy [332]	CS / hospital	16 (37.5 % female), median age: 61 y	back pain (25%) (no duration or localisation reported), claudication (12.5%)	abdominal pain (62.5%), ureteral colic pain (19%), testicular pain (19%), ureteral obstructive disease (75%), constitutional symptoms (75%), oliguria (12.5%), inferior vena cava syndrome (6%), rheumatoid arthritis (6%), dyspnoea (6%)	-	-
Warnatz et al., 2005, Germany [333]	CS / hospital	20 (30% female), median age RPF (49 y) and IAAA (62 y)	back pain (69%) (no duration or localisation reported)	pain (94%), abdominal pain (44%), flank pain (31%), weight loss (56%), renal failure (5%)	-	leriche syndrome (10%)
Paravastu et al., 2009, UK [304]	CS / hospital	38 (8% female), mean age: 69 y	back pain (or abdominal pain) (42%) (no duration or localisation reported)	hypertension (58%), ischaemic heart disease (34%)	-	58% incidental finding, 78% smokers, male:female = 12:1
Zhou et al., 2015, China [334]	CS / hospital	30 (23% female), mean age: 56.7 y	back pain (13%) (no duration or localisation reported)	flank pain (26.7%), abdominal pain (13.3%), weight loss (30.0%), fatigue (20.0%), anorexia (16.7%), nausea and vomiting (16.7%), lower extremity oedema (13.3%), oliguria (13.3%), asymptomatic (13.3%), frequency and urgency (10.0%), anuria (6.7%), haematuria (6.7%), scrotal oedema (3.3%), fever (3.3%)	-	smoking (60%), hypertension (53.3%)
Ha et al., 2011, Korea [335]	CS / hospital	27 (19% female), mean age: 55.7 y	back pain (3.7%) (no duration or localisation reported)	flank pain (37%), abdominal pain (26%), fever (26%), weight loss (26%), generalised weakness (26%), oliguria (26%), nausea / vomiting (22%), lower extremity oedema (22%), anorexia (19%), haematuria (15%), constipation (11%), urinary frequency (7%), thromboembolism (4%), anejaculation (4%)		hypertension (37%), dyslipidaemia (7%), smoking (52%)
Adler et al., 2008, Switzerland [320]	CHS / outpatient department	9 (22% female), mean age: 58.5 y	back pain (33%) (no duration or localisation reported)	abdominal pain (11%), hydronephrosis (100%), malaise (56%)	diagnostic methods: chest-x-ray to rule out malignancy	double J stenting in 78%; 67% treated for hypertension; all patients experienced regression of IRF in CT / MRI
Fry et al., 2008, UK [362]	CHS / hospital	24 (8% female), mean age: 63 y	back pain (58%) (no duration or localisation reported)	not reported	-	-
Kardar et al., 2002, Pakistan [325]	CHS / tertiary care referral centre	12 (25% female), mean age: 48.5 y	back pain (25%), LBP (8%) (no duration reported), migraine (8%)	loin pain (50%), abdominal pain (33%), flank pain (8%), groin pain (8%), bilateral ureteral obstruction (58%), dysuria (33%), weight loss (25%), fever (17%), leg swelling (17%), nausea / vomiting (8%), oliguria (8%), hydrocele (8%)	-	pain relief in 4 to 10 days after start of prednisolone therapy, obstruction relief in 6 to 8 weeks in (80%)
Simone et al., 2008, Italy [312]	CHS / not reported	6 (all male), mean age: 47 y	back pain (100%) (no duration or localisation reported)	hydronephrosis (17%)	-	-
Zhang et al., 2017, China [336]	CHS / hospital	RPF: 19 (32% female, mean age: 56.7 y); Lymphoma: 23 (39% female, mean age: 57.4 y)	back pain (79%) (no duration or localisation reported)	fatigue (37%), fever (32%), proteinuria (11%)	diagnostic methods: laboratory (high ESR in 16%)	-
Moroni et al., 2006, Italy [305]	CHS / hospital	17 (41% female), mean age: 56 y	back pain (or abdominal pain) (65%) (no duration or localisation reported)	ureteral obstruction (100%) plus unilateral hydronephrosis (29%) or bilateral hydronephrosis (71%), weakness / weight loss (35%), polyuria (35%), oligoanuria (18%), arterial hypertension, leg oedemas (18%), mild fever (6%)	-	-
Ceresini et al., 2015, Italy [306]	CCS / hospital	idiopathic RPF: 73 (37% female, mean age: 55 y); Controls: 71 (sex and age matched with idiopathic RPF)	lumbar back pain (or abdominal pain) (83%) (no duration reported)	constitutional symptoms (74%), testicular symptoms (59%), deep vein thrombosis (15%), constipation (29%), renal involvement (ureteral obstruction (71%), acute renal failure (41%))	-	-
Brandt et al., 2011, Germany [321]	register study / hospital	204 (31.9% female), mean age: 55.6 y	back pain (66%) (no duration or localisation reported)	flank pain (66.3%), lower abdominal pain (27.3%), upper abdominal pain (25.0%), leg pain (20.4%), fatigue (52.9%), weight loss (36.6%), malaise / vomiting (32.0%), night sweats (27.9%), fever (19.8%) later: hydronephrosis (95.6%), vascular complication (27.5%), renal atrophy (22.5%), large bowel compression (2.0%)	-	pre-existing immune disease (9.8%), coexisting fibrotic changes in other organ systems (3.4%), history of smoking (75.6%; active: 73.9%)
Pipitone et al., 2010, Germany [337]	NR / -	mean age: 40-60; male:female = 2-3:1	back pain is leading symptom together with abdominal pain or flank pain (no duration or localisation reported), claudication abdominally or of the lower extremities	sometimes colic pain, oedema of the lower extremities, occasionally thrombophlebitis, scrotal swelling, varicocele, hydrocele, fever, fatigue, weight loss	diagnostic methods: physical examination (tender abdomen), laboratory: ESR / CRP elevated, anaemia, leucocytosis, imaging: US, duplex-US, CT, MRI, CTA / MRA, PET-CT	-
Li et al., 2011, China [294]	NR / -	-	LBP (most common symptom) (no duration reported)	insidious onset, abdominal pain, malaise, fever, anorexia, weight loss, unilateral or bilateral lower limb oedema, scrotal swelling	-	association with other autoimmune diseases, may relate to inflammatory abdominal aortic aneurysm
Liu et al., 2014, China [301]	NR / -	male:female = 2-3:1, mean age: 55-60 y	LBP (most common symptom) (no duration reported)	insidious onset, abdominal pain, malaise, fever, anorexia, weight loss, unilateral or bilateral lower limb oedema, scrotal swelling	-	association with autoimmune diseases, most common complication: acute and chronic renal failure secondary to ureteral obstruction
Pipitone et al., 2012, Italy [303]	NR / -	mean age: 55-60 y, male:female = 2-3:1	LBP (most common symptom) (no duration reported), colicky or dull	abdominal pain and flank pain (most common presenting symptoms), varicocele, hydrocele, scrotal swelling, constipation, nausea, vomiting, fever, weight loss, fatigue, night sweats	-	-
Hara et al., 2014, Japan [338]	NR / -	-	back pain (no duration or localisation reported)	50% symptom free, abdominal pain, lower limb oedema, ureteral obstruction (45-65%): hydronephrosis or renal failure	diagnostic methods: CT, MRI	-
Burkhardt Soares et al., 2008, Germany [339]	NR / -	male:female = 2-3:1; age: 50-60	back pain (no duration or localisation reported), claudication	flank pain, testicular pain, upper and lower abdominal pain, pain in iliac fossa, fatigue, appetite & weight loss, fever, headache, nausea, vomiting, deep vein thrombosis, leg oedema, hydrocele, varicocele, polyuria, pollakiuria, dysuria, oliguria, UTI, erectile dysfunction, obstipation, hypertension, bladder dysfunction	diagnostic confirmation = CT-guided biopsy	DDx: malignancy, other inflammatory reactions
Caiafa et al., 2013, Spain [340]	NR / -	age: 40-65 y, male:female = 2-3:1	LBP (no duration reported), claudication	malaise, anorexia, weight loss, low-grade fever, pain (flank, abdomen), extremity oedema, deep vein thrombosis, scrotal swelling, varicocele, hydrocele, constipation, intestinal ischaemia	-	rare condition, 56-100% obstructive uropathy (oliguria, anuria nausea, vomiting and altered consciousness)
Cronin et al., 2008, USA [341]	NR / -	age: 40-60 y, male:female = 2-3:1	LBP (no duration reported) (severe, dull, increasing)	early symptoms: abdominal pain, flank pain, lower extremity swelling and discomfort late symptoms: deep venous thrombosis, anuria, nausea, vomiting, altered consciousness relating to uraemia, hypertension, mesenteric ischaemia, bowel obstruction renal or ureteral involvement	-	diagnosis often delayed; DDx: malignancy, drugs, therapy or chemotherapy, amyloidosis, infection, renal trauma, haemorrhage, inflammatory conditions, after irradiation
Fairweather et al., 2013; UK [363]	NR / -	age: 40-60, male:female = 2-3:1	LBP (90%) (no duration reported) (dull, non-colicky pain, not affected by movement)	abdominal pain (90%), loss of appetite, weight loss, low-grade fever, loin pain later: progressive ureteric obstruction (usually bilateral) with loin pain (colicky)	-	70% cause unknown, common associated finding: hydronephrosis
Swartz, 2009, USA [342]	NR / -	-	back pain (no duration or localisation reported)	abdominal pain, constitutional symptoms, ureteral obstruction, intestinal or biliary-pancreatic involvement, lower-extremity venous obstruction, aortic or branch arterial compression, involvement of other pelvic organs, peripheral nerve involvement	-	secondary RPF: vasculitis, granulomatosis, inflammation in patients with Crohn’s disease, primary biliary cirrhosis, granulomatosis with polyangiitis, Sjogren’s syndrome, Chester-Erdheim disease or other autoimmune syndromes; DDx: malignant process, drugs, asbestos
Palmisano et al., 2009, Italy [343]	NR / -	age: 50-60 y, male:female = 2-3:1	LBP (80%) (no duration reported) (insidious, persistent, dull, unmodified by movement or rest, colicky if ureteral involvement), claudication	testicular pain, fatigue, anorexia, weight loss, low-grade fever, scrotal swelling, varicocele and hydrocele, oedema, deep vein thrombosis of the lower limbs, constipation, nausea, and vomiting	-	most frequent complication: ureteral obstruction (50-80%); DDx: inflammatory myofibroblastic tumour, sclerosing mesenteritis, Erdheim-Chester-disease,
Vaglio et al., 2005, 2006, 2006-2, 2007, 2011-2, 2016, Italy [344–347, 349, 350]	NR / -	age: 40-60 y, male:female = 2-3:1	back pain / LBP (no duration reported) (radiating to abdomen, persistent, insidious, dull, constant, colicky if ureteral involvement, not exacerbated by movement or palpation, transiently responds to NSAIDs), claudication	pain (80%, usually dull, poorly localised: side pain, abdominal pain), constitutional symptoms (fatigue, anorexia, weight loss, nausea, vomiting, myalgia, low-grade fever (40-80%), sleep disturbance), anaemia, constipation, lower limb oedema and / or deep venous thrombosis, varicocele, hydrocele, testicular pain, erectile dysfunction, small bowel obstruction, intestinal ischaemia, obstructive uropathy (50% or more), haematuria, polyuria, urinary infections, urinary frequency, dysuria, oligoanuria, symptoms related to uraemia, hypertension, fluid and electrolyte disturbance	diagnostic methods: physical examination: (abdominal or lumbar tenderness, palpable, pulsatile, and tender abdominal mass, periumbilical bruit) laboratory: ESR, CRP elevated (> 80%), anaemia, autoantibodies positive, serum IL-6 elevated; diagnostic confirmation: CT, MRI, biopsy (percentage of biopsy-proven cases: 24-77%)	associated diseases: autoimmune diseases; risk factors: asbestos exposure, smoking, ergot derivates; delay between onset of symptoms and diagnosis
Geoghegan et al., 2007, Ireland [351]	NR / -	incidence: 1/200.000, mean age: 40-60, male:female = 2-3:1	back pain (no duration or localisation reported), neurological manifestations	generalised malaise, loss of appetite, lethargy, nausea or vomiting; long-term complications: hydrocele formation, scrotal oedema, duodenal or small bowel obstruction, common bile duct compression, obstruction of the large bowel	diagnostic methods: physical examination (hypertension, pyrexia), imaging: IVU (past), US, CT, MRI	delay in diagnosis, associated with SLE, granulomatosis with polyangiitis, polyarteritis nodosa, ankylosing spondylitis, Hashimoto, sclerosing cholangitis, hypoplasia of the right hepatic lobe, amyloidosis, diffuse idiopathic skeletal hyperostosis, actinomycosis, asbestosis exposure
Gornik et al., 2008, USA [307]	NR / -	incidence: 3-10%	back pain (or abdominal pain) with fever (no duration or localisation reported)	idiopathic isolated aortitis: constitutional symptoms, abdominal pain, elevated inflammatory markers, acute renal failure	diagnostic methods: laboratory (ESR, CRP, complete blood count, kidney / liver function, blood cultures, rheumatological screening); imaging: CTA / MRA, US, PET-CT, biopsy	risk factors: tobacco use, younger age, family history of AAA; DDx: lymphoma
Mahajan et al., 2014, USA [308]	NR / -	-	poorly localised pain in back (no duration reported)	flank pain, abdominal pain, constitutional symptoms, acute renal failure	diagnostic methods: laboratory (elevated inflammatory markers)	diagnostic delay
Vaglio et al., 2011, Italy [348]	RCT / hospital	prednisone group (P) (18, 33% female, mean age: 56 y); tamoxifen group (T) (18, 39% female, mean age: 61 y)	back pain (or abdominal pain) (P: 89%, T: 94%)	fatigue, anorexia, weight loss, low-grade fever, diffuse myalgias, arthralgias (P: 83%, T: 72%); testicular symptoms (testicular pain, varicocele, hydrocele) (P: 58%, T: 55%), constipation (P: 33%, T: 22%), ureteral obstruction (P: 78%, T: 72%)	-	6% of prednisone group and 39% of tamoxifen group relapsed while on treatment

*AAA* abdominal aortic aneurysm, *AIP* autoimmune pancreatitis*, CCS* case-control study*, CHS* cohort study*, CR* case report, *CRP* c-reactive protein*, CS* case series*, CT* computed tomography, *CTA* computed tomography angiography*, ED* emergency department, *ESR* erythrocyte sedimentation rate, *FDG-PET* fluorodeoxyglucose-positron emission tomography*, IAAA* inflammatory abdominal aortic aneurysm*, IgG4* immunoglobulin G4*, IL-6* interleukin 6*, IRF* idiopathic retroperitoneal fibrosis, *IVU* intravenous urogram*, LBP* low back pain, *MRA* magnetic resonance angiography*, MRI* magnetic resonance imaging*, NR* narrative review, *NSAIDs* non-steroidal anti-inflammatory drugs, *PET* positron emission tomography*, RCT* randomised controlled trial*, RPF* retroperitoneal fibrosis*, SLE* systemic lupus erythematosus*, TCC* transitional cell carcinoma*, US* ultrasound, *UTI* urinary tract infection*, y* years

#### Inflammatory abdominal aortic aneurysm

Inflammatory abdominal aortic aneurysms represent a subtype of aortic aneurysm characterised by the thickening of the aortic wall and periaortic tissue, occasionally involving fibrotic remodelling. A total of two case reports, one case-control study, one cohort study, and two narrative reviews pertaining to IAAA linked with LBP were identified (Table 16). The incidence of LBP varies between 58% [364] and 80% [365]. Predominantly, it presents as chronic LBP, often accompanied by typical symptoms such as anorexia, fatigue, night sweats, nausea and vomiting [97, 111, 121, 365, 366] or lower abdominal pain [111, 121, 365, 366]. While the two case reports centred around female individuals, findings from the case control and case cohort studies indicated a higher prevalence among males. The typical age of onset ranged from 50 to 70 years. Notably, the narrative review highlighted that IAAAs constitute 2-10% of all AAAs [366]. This review further underscored the tendency for IAAA patients to exhibit a younger age profile compared to those with an AAA [121].

**Table 16:** Inflammatory abdominal aortic aneurysms presenting with back pain

**Author / year/ country**	**Design / setting**	**Patient(s)**	**Spinal symptoms**	**Extraspinal symptoms**	**Diagnostic confirmation**	**Other information / comment**
Ahlawat et al., 2002, UK [97]	CR / ambulatory care	female, 71 y	chronic LBP (insidious onset, non-radiating, moderate intensity, worse at night, partially relieved with acetaminophen or aspirin; constant, dull, aching sensation)	anorexia (weight loss of 6.8 kg), decreased energy, occasional night sweats	abdominal CT	-
Sharif et al., 2008, UK [111]	CR / ED	female, 32 y	chronic LBP	lower abdominal pain; end of first week in hospital: increasing pain, nausea, and vomiting	CT, biopsy	-
Tambyraja et al., 2004, Scotland [364]	CHS / hospital	24 (8% female), median age 69 y	back pain (58%) (no duration or localisation reported)	abdominal pain (58%), collapse (33%), haematemesis (4%), haematuria / oliguria / renal failure (4%)	-	contained retroperitoneal haematoma (67%), free intraperitoneal blood (12,5%); in-hospital mortality (42%)
Goldoni et al., 2014, Italy [365]	CCS / hospital	90 (31% female, mean age: 58 y)	LBP (80%) (no duration reported)	abdominal pain (80%), ureteral obstruction (76%), systemic symptoms (fatigue, anorexia, weight loss, fever, diffuse myalgia, and arthralgia) (71%), testicular symptoms (testicular pain, varicocele, hydrocele) (63%), acute renal failure (47%), constipation (24%), deep venous thrombosis (13%)	-	-
Kasashima et al., 2011, Japan [366]	NR / -	patients with IAAA tend to be younger than patients with AAA	nonspecific dull back pain (no duration or localisation reported)	nonspecific and dull abdominal pain, low-grade fever, weight loss, general fatigue	diagnostic methods: laboratory (elevated: ESR, CRP, white blood counts; frequent: elevated serum IgG levels and autoantibodies (ANA))	2-10% of all AAAs
Tang et al., 2005, UK [121]	NR / -	patients are 5-10 years younger than in AAA	back pain (no duration or localisation reported) (80%)	flank or abdominal pain (80%), weight loss and anorexia (40%), very few asymptomatic	diagnostic methods: physical examination (tender abdominal pulsatile mass (30%)), laboratory (ESR elevated (40-88%), raised temperature / white cell count, elevated CRP); imaging: US, CT, MRI, IVU (past), nuclear medicine	risk factors: coincidence with smoking (77-100%), positive family history (17%), HLA-DR B1 locus in IAAA

*AAA* abdominal aortic aneurysm, *ANA* antinuclear antibody,* CHS* cohort study*, CCS* case control study*, CR* case report*, CRP* c-reactive protein*, CT* computed tomography*, ED* emergency department*, ESR* erythrocyte sedimentation rate*, HLA-DR* human leukocyte antigen – DR isotype*, IAAA* inflammatory abdominal aortic aneurysm, *IVU* intravenous urogram, *LBP* low back pain, *MRI* magnetic resonance imaging*, NR* narrative review, *US* ultrasound,* y* years

### Myocardial infarction

One case report, two case series, one chart review study, and two observational studies documenting instances of myocardial infarction presenting with back pain were identified (Table 17). The exact location of back pain was often not reported. In one case report, one of the patients presented with increasing back pain over a two-day period. Despite being in shock at admission, the initial presenting symptom was back pain [367]. An observational study indicated a higher prevalence of back pain as a symptom of myocardial infarction in women compared to men, whereas men typically presented more frequently with chest pain. Other accompanying symptoms were, for example, chest pain or discomfort, shoulder pain, cold sweat, or nausea [368–372].

**Table 17:** Myocardial infarction presenting with back pain

**Author /year / country**	**Design / setting**	**Patient**	**Spinal symptoms**	**Extraspinal symptoms**	**Diagnostic confirmation**
Ichinose et al., 2009, Japan [367]	CR / hospital	female, 68 y	acute back pain (no localisation reported), severe, migratory from chest	-	coronary angiogram, CE-CT
		male, 66 y	acute back pain (no localisation reported), exacerbated over 2 days	state of pre-shock on admission	CE-CT, ultrasonic echocardiography
Kosuge et al., 2006, Japan [371]	CS / hospital	457 (23.2% female), mean age: f: 72 y, m: 62 y	acute back pain (f: 24%, m: 12%) (no localisation reported)	anterior chest pain (f: 89%, m: 88%), throat and neck pain (f: 13%, m: 5%), left shoulder pain (f: 12%, m: 5%), left arm / forearm / hand pain (f: 11%, m: 5%), jaw pain (f: 9%, m: 3%), right shoulder pain (f: 9%, m: 4%), right arm / forearm / hand pain (f: 8%, m: 3%), epigastric region (f: 8%, m: 9%), cold sweating (f: 63%, m: 78%), shortness of breath (f: 62%, m: 52%), nausea (f: 49%, m: 36%), vomiting (f: 25%, m: 15%)	-
Lovlien et al., 2006, Norway [372]	CS / hospital	533 (28% female), mean age: f: 61.2 y, m: 58.5 y	back pain (f: 26%, m: 16%) (no duration or localisation reported)	chest symptoms (pain, discomfort, pressure, tightness) (f: 86%, m: 92%), left arm pain (f: 52%, m: 49%), right arm pain (f: 34%, m: 27%), between scapulae pain (f: 34% , m: 24%), shoulder pain (f: 32%, m: 28%), jaw / throat pain (f: 32%, m: 24%), headache (f: 19%, m: 16%), abdominal pain (f: 18%, m: 15%), sweating (f: 58%, m: 56%), dyspnoea (f: 47%, m: 38%), nausea (f: 46%, m: 29%), fatigue (f: 46%, m: 38%), dizziness (f: 39%, m: 33%), palpitations (f: 38%, m: 18%), hot flashes (f: 29%, m: 22%), fainting (f: 13%, m: 7%)	-
Berg et al., 2009, Sweden [369]	chart review study / hospital	225 (23.1% female), mean age: f 61.2 y, m: 59.3 y	back pain (f: 42.3%, m: 14.5%) (no duration or localisation reported)	chest pain (f: 88.5%, m: 94.8%), arm and shoulder pain (f: 61.5%, m: 59.5%), neck pain (f: 25%, m: 22%), abdominal pain (f: 15.4%, m: 7.5%), jaw pain (f: 7.7%, m: 9.2%), nausea (f: 53.8%, m: 29.5%), diaphoresis (f: 34.6%, m: 42.8%), fatigue (f: 30.8%, m: 19.1%), dizziness (f: 17.3%, m: 7.5%), vomiting (f: 13.5%, m: 6.9%), palpitations (f: 11.5%, m: 2.9%), syncope / light-headedness (f: 3.8%, m: 9.8%)	-
Lawesson et al., 2018, Sweden [368]	observational study / hospital	532 STEMI patients (24% female), (mean age: f: 69.7 y vs m: 64.3 y)	back pain (m: 12%, f: 29%) (no duration or localisation reported)	chest pain / discomfort (m: 93%, f: 74%), throat / neck pain (m: 18%, f: 34%), shoulder pain (m: 15%, f: 33%), nausea (m: 29%, f: 49%), fear (m: 17%, f: 31%), cold sweat (no gender difference), fatigue and / or weakness (no gender differences)	-
Culic et al., 2002, Croatia [370]	observational study / hospital	1996 (30.1 % female), mean age: f: 63 y, m: 57 y	back pain (f: 10.6%, m: 5.2%) (no duration or localisation reported)	any pain (m: 93.2%, f: 86.2%), chest pain (m: 87.6%, f: 79.7%), left arm pain (m: 65.8%, f: 71%), right arm pain (m: 40.1%, f: 47.4%), left shoulder pain (m: 45.2%, f: 43.8%), right shoulder pain (m: 35.3%, f: 30.8%), epigastric pain (m: 16.1%, f: 13.3%), neck pain (m: 10.7%, f: 17.3%), jaw pain (m: 5.5%, f: 9.2%), headache (m: 4.4%, f: 10.8%), only non-chest pain (m: 4.9%, f: 8.2%); any non-pain symptom (m: 71.2%, f: 84.2%), sweating (m: 59.7%, f: 48.1%), weakness (m: 48.1%, f: 45.8%), nausea (m: 40.9%, f: 57.4%), dyspnoea (m:34.3%, f: 48.4%), vomiting (m: 17.6%, f: 21%), belching (m: 16.9%, f: 12.8%), cough (m: 8.1%, f: 15.5%), vertigo (m: 5.7%, f: 7.8%), faintness (m: 5.1%, f: 3.5%), hiccups (m: 3.4%, f: 1.5%), tinnitus (m: 1.9%, f: 2.8%)	-

*CE-CT* contrast-enhance computed tomography*, CR* case report, *CS* case series*, f* female*, m* male, *STEMI* ST-elevation myocardial infarction*, y* years

### Gastrointestinal diseases

#### Gallstone disease / cholecystitis

Gallstone disease and cholecystitis usually present with colicky upper abdominal pain. Two case reports and one randomised controlled trial (RCT) investigating LBP as an initial complaint associated with gallstone disease or cholecystitis were identified (Table 18). The RCT compared two treatment strategies for managing gallstone disease and reported baseline symptoms encompassing pain radiating to the back. Among the case reports, one focussed on cholecystitis [373], while the other delved into symptomatic cholecystolithiasis [374], both involving female patients. LBP can present both acutely (particularly with inflammation) [373] as well as chronically (with symptomatic cholecystolithiasis without inflammation) [374]. Accompanying symptoms often comprise abdominal pain [373, 374], predominantly localised in the right upper quadrant or epigastric region [375]. Other symptoms include gastrointestinal symptoms such as fat intolerance, nausea and vomiting, diarrhoea, and difficulty in defecation [375].

**Table 18:** Gallstone disease / cholecystitis presenting with back pain

**Author / year / country**	**Design / setting**	**Patient(s)**	**Spinal symptoms**	**Extraspinal symptoms**	**Diagnostic confirmation**
Kinoshita et al., 2002, Japan [373]	CR / not reported	female, 75 y	acute LBP	upper abdominal pain at night	MRCP, PTGBA
Petersen et al., 2018, USA [374]	CR / ambulatory primary care -> ambulatory specialist care -> ED	female, 30 y	chronic LBP (intermittent, persistent and sharper), upper back pain radiating to arms and legs	abdominal pain, left posterior lateral thoracic pain, left sided torso pain, morning stiffness in all joints, increased sweating 2 to 3 times a week	pelvic ultrasound
van Dijk et al., 2019, The Netherlands [375]	RT / hospital	1067 (74% female), mean age: 48.5 y	pain radiating to the back (68%) (no duration or localisation reported)	pain located in right upper quadrant or epigastric region (91%), pain radiating to the chest (25%), nausea and vomiting (60%), bloating (51%), fat intolerance (46%), burping (40%), flatulence (36%), acid reflux (31%), difficult defecation (19%), diarrhoea (18%)	-

*CR* case report, *ED* emergency department, *LBP* low back pain, *MRCP* magnetic resonance cholangiopancreatography*, PTGBA* percutaneous transhepatic gallbladder aspiration*, RT* randomised trial*, y* years

#### Pancreatitis

Pancreatitis, an inflammatory condition of the pancreas, can either manifest acutely or chronically. Two case reports, one narrative review and one guideline were found detailing LBP as presenting symptom attributed to pancreatitis (Table 19). Accompanying symptoms were abdominal pain, loss of appetite and weight, or jaundice [376–379]. Furthermore, one narrative review [378] was identified, outlining back pain as radiating pain originating from the epigastric region.

**Table 19:** Pancreatitis presenting with back pain

**Author / year / country**	**Design / setting**	**Patient**	**Spinal symptoms**	**Extraspinal symptoms**	**Diagnostic confirmation**	**Other information / comment**
Matsubara et al., 2005, Japan [376]	CR / hospital	male, 63 y	LBP (no duration reported)	appetite loss, jaundice	PTGBD, wedge biopsy, intraoperative cholangiography	-
Nishimura et al., 2004, Japan [377]	CR / hospital	female, 47 y	sudden onset acute back pain (no localisation reported)	upper abdominal pain	ERC / biopsy	-
Rompianesi et al., 2017, Italy [378]	NR / -	-	pain radiating to the back (no duration or localisation reported)	epigastric pain (persistent, severe) or diffuse abdominal pain starting in the epigastric region	diagnostic methods: radiological tests	serum lipase and amylase do not reliably diagnose acute pancreatitis, especially with prolonged interval between symptom onset and testing
Okazaki et al., 2009, Japan [379]	guideline / -	-	back pain (15%) (no duration or localisation reported)	1/3 to 1/2 with obstructive jaundice or mild abdominal pain, weight loss, in some cases: polydipsia / polyuria or malaise, xerostomia / xerophthalmia or hydronephrosis	diagnostic methods: laboratory (elevated biliary enzymes, pancreatic enzymes and total bilirubin) US / CT / MRI, Ga-67 and FDG accumulation, ERCP; IgG4 highest diagnostic value but not specific	association with: sclerosing cholangitis, diabetes mellitus, sclerosing sialadenitis / dacryoadenitis or retroperitoneal fibrosis; frequently: pancreatic exocrine and endocrine dysfunction

*CR* case report, *CT* computed tomography, *ERC* endoscopic retrograde cholangiography*, ERCP* endoscopic retrograde cholangiopancreatography*, FDG* fluorodeoxyglucose*, Ga-67* gallium-67*, IgG4* immunoglobulin G4*, LBP* low back pain*, MRI* magnetic resonance imaging *NR* narrative review*, PTGBD* percutaneous transhepatic gallbladder drainage,*. US* ultrasound,* y* years

#### Miscellaneous

Five case reports featuring different gastrointestinal diseases as the primary presentation of LBP were identified (Table 20). These include intussusception [23], coeliac disease [380], pyeloduodenal fistula [381], liver abscess [34], and acute appendicitis [382]. While LBP predominantly presents acutely [34, 381, 382], it could also manifest chronically, as observed in coeliac disease [380]. Depending on the disease, accompanying symptoms such as occasional fever with nausea and diarrhoea (intussusception) [23] or weight loss (coeliac disease) [380, 382] indicated an extravertebral origin of LBP. However, the patient with the liver abscess presented solely with acute LBP [34]. Diagnosis was confirmed either by imaging [23, 34, 381, 382] or biopsy [380, 382].

**Table 20:** Miscellaneous gastrointestinal diseases presenting with back pain

**Author / year / country**	**Design / setting**	**Patient / diagnosis**	**Spinal symptoms**	**Extraspinal symptoms**	**Diagnostic confirmation**
Barbee et al., 2008, USA [23]	CR / ED	female, 20 y, intussusception	chronic LBP, radiating to the left anterior / superior hip; intermittent, aching, spasmodic, 10/10 at its worst; hip pain on most days for the past 4 months	occasional fever, occasional nausea, and diarrhoea	CT
Hoffman et al., 2004, USA [380]	CR / hospital	male, 42 y, coeliac disease	chronic LBP, no significant improvement with NSAR, difficulty walking	hip, ankle, and feet pain, weight loss	biopsy
Kobayashi et al., 2018, USA [381]	CR / ED	female, 89 y, pyeloduodenal fistula	acute LBP, radiating to the legs, progressively worsening, paroxysms of pain involving lumbar region, spasmodic cramping of bilateral buttocks and thighs	-	Tc-99m MAG3-scintigraphy
Tseng et al., 2015, Taiwan [34]	CR / ED	male, 46 y, liver abscess	acute right LBP	-	CT
Kuday Kaykisiz et al., 2018, Turkey [382]	CR / ED	male, 36 y, acute appendicitis	acute back pain (no localisation reported)	urinary difficulty, anorexia	CT + surgery / histological examination

*CR* case report,* CT* computed tomography*, ED* emergency department, *LBP* low back pain*, NSAR* non-steroidal anti-rheumatic drug*, Tc-99m MAG3-scintigraphy* Technetium-99m mercaptoacetyltriglycine scintigraphy*, y* years

### Paraspinal compartment syndrome

Compartment syndrome, marked by fluid accumulation in a muscle compartment leading to increased pressure and compromised blood supply, predominantly affects the lower leg but can involve other muscle groups, including paraspinal muscles. Identified were ten case reports and one narrative review reporting instances of paraspinal compartment syndrome in individuals presenting with acute LBP (Table 21). Across all case reports, only males were affected. The pain was described as abrupt, severe, sharp, throbbing, constant, or exacerbated by movement [22, 29, 383–390]. Typical accompanying symptoms were pain radiating into the leg and groin, numbness, and sensory deficits [22, 29, 256, 383–385, 389, 390]. Other extravertebral signs and symptoms were generally absent, aside from occurrences of dark urine due to myoglobinuria [22, 385] or fatigue [389]. Notably, all case reports described symptoms that started after weightlifting or heavy exercising. The review also reported aetiologies such as downhill skiing, surfboarding, or weightlifting, as well as iatrogenic causes like aortic or gastric bypass. An elevated creatine kinase (CK) in patients with LBP is a diagnostic clue for paraspinal compartment syndrome. Diagnosis was confirmed by imaging studies or measurement of intramuscular pressure. Compartment syndrome is an emergency which requires immediate referral when suspected.

**Table 21:** Paraspinal compartment syndrome presenting with low back pain

**Author / year / country**	**Design / setting**	**Patient(s)**	**Spinal symptoms**	**Extraspinal symptoms**	**Diagnostic confirmation**	**Other information / comment**
Allerton et al., 2012, Australia [22]	CR* / ED	male, 25 y	LBP (no duration reported) (severe, gradual increase in severity over 4-6 hours, exacerbated by movement, radiating to right groin), altered sensation affecting his right leg	dark urine	MRI, paraspinal compartment pressure	-
Anaya et al., 2014, USA [383]	CR* / ED	male, 35 y	acute LBP (abrupt in onset, sharp, 10/10 on pain scale, constant, midline with no radiation, exacerbated by any flexion, extension, or rotation), hip pain, numbness, and lack of sensation in the lower portion of his back (right > left)	-	MRI	-
Chavez et al., 2013, USA [29]	CR* / ED	male, 25 y	acute LBP (severe, rapidly progressive stabbing and radiating down the left leg), difficulty walking	-	MRI	-
Minnema et al., 2008, USA [384]	CR* / ED	male, 32 y	acute LBP (excruciating, right sided, radiating down the posterior side of his leg), tenderness	-	MRI, paraspinal compartment pressure, elevated CK	-
Wik et al., 2010, Canada [386]	CR* / ED	male, 30 y	acute LBP (severe, bilateral, throbbing, exacerbated by lateral movement, diminished when standing perfectly straight)	-	MRI, paraspinal compartment pressure, elevated CK	-
Khan et al., 2005, Australia [385]	CR* / ED	male, 35 y	acute LBP (spontaneous, severe, unrelenting, right sided, exacerbated by movement, radiating across the abdomen to the groin), numbness in right lumbosacral area	dark urine	MRI, paraspinal compartment pressure, elevated CK	-
Paryavi et al., 2010, USA [387]	CR* / ED	male, 20 y	acute LBP (progressive, severe, debilitating back pain, bilateral)	-	MRI, paraspinal compartment pressure, elevated CK	-
Karam et al., 2010, USA [388]	CR* / ED	male, 23y	acute LBP (rapidly increasing, throbbing)	-	MRI, elevated CK	-
Kitajima et al., 2002, Japan [389]	CR* / ED	male, 25 y	acute LBP (severe, during night, decreased sensation)	fatigue	MRI, paraspinal compartment pressure, elevated CK	-
Xu et al., 2009, China [390]	CR* / not reported	male, 25 y	acute LBP (increasing during training, subsided afterwards, discomfort remained), sensation of tightness (during training)	-	MRI, paraspinal compartment pressure	-
Nathan et al., 2012, USA [391]	NR / -	11 case reports (10% female, 24-67 y)	excruciating LBP (100%) (no duration reported), localised tenderness over the paraspinal region, localised sensory loss paraspinal region, atrophy of paraspinal muscle (9.1%)	-	diagnostic methods: physical examination (absent abdominal sounds, deep abdominal tenderness on palpation) laboratory (CK elevated (90.1%))	aetiology: downhill skiing (27.3%), direct trauma (9.1%), aortic bypass (27.3%), surfboarding (9.1%), gastric bypass (9.1%), weightlifting (9.1%)

* following weight lifting, cross-fit exercise, skiing, or surfboarding

*CK* creatine kinase*, CR* case report*, ED* emergency department, *LBP* low back pain*, MRI* magnetic resonance imaging, *NR* narrative review*, y* years

### Gynaecological diseases

#### Endometriosis

Endometriosis, a gynaecological disease characterised by the presence of endometrial tissue outside the uterus, predominantly affects women in their childbearing years. Given the varying locations of the endometriosis foci, a range of non-specific symptoms, including LBP, can arise. A total of ten case reports, two case series, ten cohort studies, one case-control-study, one cross-sectional study, four narrative and one systematic review were identified (Table 22). The incidence of LBP associated with endometriosis varied greatly from 14.48% [392] to 93.4% [393]. An observational study by Darai et al. [392] suggested a causal relationship between LBP and endometriosis, noting LBP improvement in 55% of women following intervention. However, 18% reported worsening or no change of LBP, indicating that not all reported LBP among endometriosis patients can be directly attributed to endometriosis. Symptoms pointing towards endometriosis were chronic LBP, cyclical LBP, and increasing pain intensity [394–400]. Furthermore, patients with endometriosis commonly complain of dysmenorrhoea (up to 90% [401]) and dyspareunia (up to 85% [401]). Other accompanying symptoms depend on the localisation of the endometriosis foci and include urological symptoms like dysuria [395, 402–404] or gastrointestinal symptoms like rectal bleeding [396], cyclic and non-cyclic dyschezia [402–404], and alterations in bowel habits (constipation, diarrhoea) [405–409]. Due to the delay between the onset of symptoms and the diagnosis of endometriosis [399, 400, 410], endometriosis should be considered, especially in young women with chronic low back pain.

**Table 22:** Endometriosis presenting with back pain

**Author / year / country**	**Design / setting**	**Patient(s)**	**Diagnosis**	**Spinal symptoms**	**Extraspinal symptoms**	**Diagnostic confirmation**	**Other information / comment**
Agrawal et al., 2006, India [394]	CR / not reported	female, 40 y	intramedullary endometriosis of the conus medullaris	chronic LBP (cyclical), sensory loss below L1	weakness in lower limbs, constipation, urinary hesitance	surgery, histological examination	-
Hsieh et al., 2010, Taiwan [395]	CR / ED	female, 42 y	ureteral endometriosis	chronic + acute LBP (cyclical, dull)	dysuria, costovertebral angle tenderness	surgery, histological examination	-
Kanthimathinathan et al., 2007, USA [396]	CR / ED	female, 30 y	intestinal endometriosis	chronic LBP (cyclical)	rectal bleeding	surgery, histological examination	-
Kondo et al., 2009, Japan [411]	CR / not reported	female, 44 y	ureteral endometriosis	chronic + acute LBP	-	biopsy, histological examination	-
Seyam et al., 2014, Saudi Arabia [412]	CR / not reported	female, 32 y	ureteral endometriosis	chronic + acute LBP	-	surgery	-
Uppal et al., 2017, USA [413]	CR / not reported	female, 39 y	endometriosis	chronic LBP (radiating)	abdominal pain	Lupron hormone antagonist therapy	-
Steinberg et al., 2014, USA [397]	CR / not reported	female, 29 y	intramedullary endometriosis of the conus medullaris	chronic LBP (cyclical, radicular)	difficulty walking	-	-
Troyer et al., 2007, USA [414]	CR / ED	female, 25 y	endometriosis	acute + chronic LBP	abdominal pain	laparoscopy and biopsy	-
Generao et al., 2005, USA [398]	CR / not reported	female, 49 y	ureteral endometriosis	chronic LBP	right flank pain, abdominal pain	biopsy	-
Kumar et al., 2012, India [415]	CR / not reported	female, 29 y	urinary tract endometriosis	back pain (no duration or localisation reported)	irritative symptoms	diagnostic laparoscopy with partial cystectomy	-
		female, 36 y		back pain (no duration or localisation reported)	irritative symptoms	-	-
Mu et al., 2014, China [416]	CS / not reported	23 females (age: 22-50 y)	ureteral endometriosis	LBP (73.9%) (no duration reported)	hypogastralgia (64.7%), dyspareunia (29.4%), menoxenia (29.4%), dysmenorrhoea (29.4%), haematuria (11.8%)	not reported	-
Carmignani et al., 2010, Italy [417]	CS / tertiary referral centre	23 females, mean age: 35.6 y	endometriosis	LBP (26.1%) (left: 17.4%, right: 8.7%) (no duration reported)	urinary symptoms (43.5 %), renal colic (17.4%) (left: 13%, right: 4.3%), arterial hypertension (4.3%)	-	-
Byrne et al., 2018, UK [403]	CHS / hospital	4721 females, median age: 35.1 y	rectovaginal endometriosis	LBP (87.9%) (no duration reported), difficulty emptying bladder	premenstrual pain, menstrual pain, non-cyclical pelvic pain, deep dyspareunia, cyclical dyschezia, non-cyclical dyschezia, bladder pain or pain passing urine, frequent bowel movements, urgent bowel movements, incomplete emptying sensation, constipation, melaena	-	-
Chudzinski et al., 2017, France [405]	CHS / hospital	17 females, mean age: 34.72 y	pelvic endometriosis	LBP (47%) (no duration reported)	dysmenorrhea (64.7%), urinary functional signs (47%), dyspareunia (35.2%), primary infertility (35.2%), recurrent pyelonephritis (35.2%), digestive functional signs (29.4%), haematuria (29.4%), dyschezia (17.6%), constipation (17.6%), algomenorrhoea (17.6%), diarrhoea (11.7%), cystalgia (5.8%)	-	-
Darai et al., 2007, France [392]	CHS / hospital	81 females, age: 33.2 y	colorectal endometriosis	LBP (49.4%) (no duration reported)	dysmenorrhea (74.1%), dyspareunia (70.4%), pain or cramping on bowel movement (63%), asthenia (55.6%), pain on defecation (38.3%)	-	-
Darai et al., 2010, France [401]	CHS / hospital	29 females, age: 41.4 y	extensive pelvic endometriosis	LBP (14.48%) (no duration reported)	dysmenorrhea, dyspareunia, diarrhoea, constipation, tenesmus, cramping, dyschezia, asthenia	-	-
Dubernard et al. 2006, France [406]	CHS / hospital	58 females, median age: 31 y	colorectal endometriosis	LBP (51.7%) (no duration reported), after surgery: LBP disappeared (57%), decreased (23%) or remained the same (20%); symptom intensity preoperative (3.5/10), postoperative (0/10)	dysmenorrhoea (84.48%), dyspareunia (74.13%), pain or cramping on bowel movement (68.97%), constipation (58.62%), asthenia (58.62%), pain on defecation (46.55%), tenesmus (37.93%), diarrhoea (25.86%), rectorrhagia (8.62%)	-	-
Redwine et al., 2001, UK [407]	CHS / hospital	84 females, age: 34.98 y	endometriosis	moderate LBP (89.55%) (no duration reported), after surgery: LBP improved (65%), worsened (3%), unchanged (32%)	uterine cramps with menses (94.03%), painful bowl movements (94.03%), constipation (92.54%), diarrhoea (92.54%), fatigue (92.54%), intestinal cramping (91.04%), menstrual pain other than cramps (88.06%), non-menstrual pelvic pain (86.57%), tenderness on exam (83.58%), dyspareunia (52.24%), pelvic pain with exercise (29.85%)	-	-
Stepniewska et al., 2011, Italy [402]	CHS / tertiary referral centre	20 females, mean age: 35 y	endometriosis	LBP (20%) (no duration reported)	dysmenorrhoea (90%), dyspareunia (85%), dyschezia (65%), dysuria (30%), renal colic (5%), recurrent cystitis (5%)	-	-
Thomassin et al., 2004, France [408]	CHS / hospital	27 females, median age: 32 y	colorectal endometriosis	LBP (26%) (no duration reported), postoperatively: LBP disappeared (57%) or decreased (43%), mean pain intensity preoperative: 6 (0-10) versus postoperative: 2.5 (0-8))	tenderness (while palpation of endometriotic nodule) (100%), dysmenorrhea (89%), dyspareunia (89%), rectal symptoms (70%), pain on defecation (63%), non-menstrual pelvic pain (41%), rectorrhagia (33%), asthenia (30%), pain or cramping on bowel movement (26%), diarrhoea and / or constipation (15%)	-	-
Ballard et al., 2010, UK [418]	CHS / hospital	113 females, mean age: 32 y	chronic pelvic pain undergoing a diagnostic laparoscopy	back pain (70%) (no duration or localisation reported)	pain in: hypochondrium, right loin, umbilicus, left loin, right iliac fossa, suprapubic, left iliac fossa, left scapula, middle back, right scapular, left sacroiliac, sacrum, right sacroiliac, legs, perineal	-	-
Schliep et al., 2015, USA [404]	CHS / hospital	190 females with endometriosis, mean age: 32 y; 147 females with other gynaecological conditions, mean age: 33.5 y	endometriosis	LBP (72.1% in patients with endometriosis (EM), 68% in patients with other gynaecological diseases (gynD), 67.7% in patients with normal pelvis (normPel)) (no duration reported)	pain at ovulation (EM: 67.4%; gynD: 49.0%; normPel: 52.2%), dysuria (EM: 22.6%; gynD: 19.1%; normPel: 11.0%), dyschezia (EM: 44.2%; gynD: 32.7%; normPel: 25.7%), pain in groin when lifting (EM: 26.3%; gynD: 27.2%; normPel: 19.9%), pain when bladder is full (EM: 53.2%; gynD: 51.0%; normPel: 42.7%), abdominal pain (EM: 51.1%; gynD: 49.7%; normPel: 44.1%), muscle / joint pain (EM: 53.3%; gynD: 46.0%; normPel: 47.7%), migraine (EM: 53.6%; gynD: 44.6%; normPel: 49.2%)	-	-
Markham et al., 2019, Australia [393]	CCS / hospital	737 females, mean age: 34.9 y (with endometriosis: 529, mean age: 34.7 y)	endometriosis	LBP in endometriosis (93.4%), LBP in women without a gynaecological complaint (70%) (no duration reported)	dysmenorrhoea (100%) in different pain intensities, pain at ovulation (87%), rectal pain (67%), dysuria (43%), pelvic pain other than during menses, ovulation, urination, or intercourse (77%), dyspareunia (85%)	-	-
Soliman et al., 2017, USA [419]	CSS / web-based survey	1269 females, mean age: 34.3 y	endometriosis	LBP 76% (no duration reported)	pelvic pain or cramping during menstrual period (88%), anxiety or stress (75%), fatigue or weariness or anaemia (75%), depressed feelings / mood swings (65%), bloating (65%), heavy bleeding during periods (51%), non-menstrual pelvic pain (47%), dyspareunia (32%)	-	-
Chiantera et al., 2017, Germany [410]	NR / -	-	-	LBP, radicular pain (no duration reported)	dysmenorrhoea, dyspareunia, dyschezia, bowel and bladder complaints, vomiting / emesis, gastric disorders, headache, dizziness, painful ovulation, irregular pelvic pain, chronic fatigue	-	long delay between onset of symptoms and official diagnosis
Jackson et al., 2006, Canada [420]	NR / -	incidence peak: 40 years	-	LBP (no duration reported)	dysmenorrhea, dyspareunia, pelvic pain, pelvic mass, infertility, bowel and bladder symptoms	diagnostic methods: physical examination often unremarkable	often asymptomatic
Engemise et al., 2010, UK [421]	NR / -	-	-	LBP (29 %) (no duration reported)	dysmenorrhoea (40-87%), chronic pelvic pain (20-80%), deep dyspareunia (19-42%), bloating (42%), lethargy (40%), constipation (29%), dyschezia (13-30%), infertility (9%), cyclical rectal bleeding (9%), diarrhoea (8%), menorrhagia (7%), haematuria (3%)	-	clinical presentation varies considerably, significant proportion of those with endometriosis being asymptomatic and diagnosed incidentally
Al Khodairy et al., 2007, Switzerland [399]	NR / -	-	-	sciatica with or without LBP (no duration reported)	pain in the hip and the buttock radiating to leg and foot, onset few days before menstruation	-	delay until diagnosis: 4 months – 15 years; cyclic pattern of sciatic pain is highly suggestive of endometriosis
Young et al., 2015, Australia [400]	SR / -	-	-	back pain (no duration or localisation reported) (“crippling”, “contractions”, “horrific”, “sharp”, “stabbing”, “overwhelmed every other sense in your body”), associated with menstrual cycle, random pain, or continuous pain	pain in pelvis, bladder, bowel, gastrointestinal tracts, and joints, in association with intercourse	-	diagnostic delay more prevalent in primary care settings

*CCS* case control study*, CHS* cohort study, *CR* case report, *CS* case series*, ED* emergency department*, EM* endometriosis*, gynD* other gynaecologic diseases*, LBP* low back pain, *normPel* normal pelvis*, NR* narrative review, *SR* sytematic review,* y* years

#### Miscellaneous

Six case reports and one narrative review encompassing various gynaecological diseases, such as benign cystadenoma [422], endosalpingiosis [423, 424], uterine fibroid [425], uterus-like structure of Müllerian origin [426], spinal intradural Müllerianosis [41], retroverted uterus, and tuboovarian abscess [399] associated with LBP, were identified (Table 23). The patients frequently presented with chronic radicular LBP, especially concurrent with the presence of tumorous tissue. Across all identified studies, notable findings during physical examinations included various neurological signs, e.g., decreasing muscular strength [426], tenderness on palpation [423], positive straight leg test [425], or sensory loss [426]. Therefore, when taking a patient’s history, more attention should be directed toward the presence of chronic LBP [41, 422–424, 426], occasionally exhibiting cyclical patterns [41, 426], primarily among pre-menopausal women [423, 425, 426]. Ahmad et al. [422] also reported abdominal swelling and pain, indicating that these aspects should also be included in history taking and physical examination as potential accompanying symptoms.

**Table 23:** Miscellaneous gynaecological diseases presenting with low back pain

**Author / year / country**	**Design / setting**	**Diagnosis**	**Patient(s)**	**Spinal symptoms**	**Extraspinal symptoms**	**Diagnostic confirmation**	**Other information / comment**
Ahmad et al., 2014, UK [422]	CR / ambulatory care	benign cystadenoma	female, 58 y	chronic LBP, weakness, paraesthesia, numbness	general abdominal swelling & pain	surgery and histological examination	-
Barresi et al., 2017, Italy [423]	CR / not reported	endosalpingiosis	female, 49 y	chronic LBP (radiating)	not reported	surgery and histological examination	-
Murphy et al., 2010, USA [425]	CR / ambulatory care	uterine fibroid	female, 44 y	acute LBP (radiating)	left leg pain	pelvic MRI	-
Sharma et al., 2007, India [426]	CR / not reported	tethered cord syndrome with a uterus-like structure of Müllerian origin	female, 24 y	chronic LBP (cyclical, radiating)	not reported	surgery and histological examination	-
Scheel et al., 2013, Germany [424]	CR / not reported	endosalpingiosis	female, 48 y	chronic LBP	not reported	surgery and histological examination	-
Barresi et al., 2006, Italy [41]	CR / not reported	spinal intradural Müllerianosis	female, 42 y	chronic LBP (in association with menstruation, worsened over time), exacerbated by sciatica mostly involving left lower limb	not reported	surgery and histological examination	-
Al Khodairy et al., 2007, Switzerland [399]	NR / -	retroverted uterus	female, 47 y	LBP (sciatic pain with L5 and S1 root involvement) (no duration reported)	-	-	-
		tuboovarian abscess	female, 25 y	acute LBP (severe right sciatic pain, unilateral with LBP; after a fall 3 weeks before admission)	low grade fever	-	delay until diagnosis: 3 weeks
		fibroids	4 females, age: not reported	unilateral sciatic pain and LBP (no duration reported)	-	-	time / delay to diagnosis from immediate to 4.5 years

*CR* case report*, LBP* low back pain*, MRI* magnetic resonance imaging*, NR* narrative review*, y* years

The inclusion of ovarian cancer invading the spine within the context of red flags remains uncertain. The Cochrane review addressing screening for malignancy in patients with LBP does not refer to ovarian cancer [36]. While this review omits all forms of cancers, it is important to note that LBP has been documented as a symptom in 23% of ovarian cancer cases [427].

### Urological diseases

#### Urinary tract infection and pyelonephritis

Urinary tract infection (UTI) can cause inflammation in any part of the urogenital tract, such as in the kidney, bladder, ureters, or urethra. It commonly presents with symptoms like painful urination, urinary frequency, and sometimes fever [428]. While cystitis usually presents with exactly these symptoms, pyelonephritis is an inflammatory disease of the kidney, which can also present with LBP. It commonly occurs in middle aged and older women. Three case reports, and one case series describing acute LBP as initial clinical presentation of pyelonephritis were included. Additionally, one case report, one cross-sectional study, and two reviews have expanded upon UTI as a potential cause of LBP (Table 24). Notably, 23.3% of patients diagnosed with pyelonephritis report LBP [428], indicating it as a reliable predictor [429]. In a majority of cases, the LBP can be localised as either left- or right-sided [430]. While UTI can be a cause of LBP, it lacks a significant association [431]. On the other hand, LBP has been identified as a symptom associated with an increased probability of urinary tract infection [432]. This connection is especially pertinent in patients with neurogenic bladder and sensory deficits, where LBP can manifest as a non-specific symptom of UTI [433]. In women presenting with acute LBP alongside accompanying symptoms such as general fatigue, signs of poor health or fever, UTI should be considered as differential diagnosis. Other clinical indicators encompass prior sexual intercourse, recent utilisation of spermicidal products, asymptomatic bacteriuria, or previous history of cystitis [433].

**Table 24:** Urinary tract infections and pyelonephritis presenting with low back pain

**Author / year / country**	**Design / setting**	**Patient(s) / diagnosis**	**Spinal symptoms**	**Extraspinal symptoms**	**Diagnostic confirmation**
Germani et al., 2002, Switzerland [434]	CR / ED	male, 52 y / pyelonephritis	acute LBP, radiating	weight loss, malaise, fever	laboratory and abdominal / renal ultrasonography
Nakamura et al., 2005, Japan [435]	CR / hospital	female, 84 y / pyelonephritis	acute LBP	malaise, fever	laboratory and abdominal / renal ultrasonography
Roy et al., 2001, France [436]	CR / hospital	1 male (72 y), 4 females (81 y, 47 y, 54 y, 65 y) / emphysematous pyelitis	LBP (no duration reported)	nausea, chills, fever	CT
Derouiche et al., 2009, Tunisia [430]	CR / hospital	5 females, 1 male (mean age: 55 y) / pyelonephritis	acute LBP (100%)	fever (83.3%), malaise (83.3%)	laboratory and abdominal / renal ultrasonography
Soler et al., 2015, Ireland [429]	CS / not reported	Malta: 9896 (age and sex not reported), the Netherlands: 15318 (age and sex not reported) / UTI	LBP (no duration reported)	flank / axilla symptom, dysuria, fever, abdominal pain / cramps, vomiting, urinary frequency or urgency, nausea	-
Kinouani et al., 2017, France [428]	CSS / hospital	340 patients, patients with urine dipstick test: median age 44 y; patients without urine dipstick test: median age 57 y / UTI	pyelonephritis: LBP (23.3%) (no duration reported)	cystitis: painful urination, urinary frequency; pyelonephritis: fever, painful urination, asthenia	-
MeReC Bulletin, 2006, UK [432]	NR / -	-	back pain (no duration or localisation reported)	dysuria, frequency, haematuria	-
Giesen et al., 2010, Ireland [431]	SR / -	-	back pain (no duration or localisation reported)	dysuria, frequency, fever, flank pain, haematuria, lower abdominal pain, nocturia, urgency, vaginal discharge	-

*CR* case report*, CS* case study*, CSS* cross-sectional study*, CT* computed tomography*, ED* emergency department*, LBP* low back pain*, NR* narrative review*, SR* systematic review*, y* years

#### Urinary kidney stone and hydronephrosis

Urinary kidney stones can develop due to various factors, potentially resulting in complications like ureteral obstructions, which can lead to colic and subsequent hydronephrosis. Only one case report was identified, where acute LBP was the leading presenting complaint (Table 25). The individual had a history of urolithiasis a few years ago. Laboratory tests and pyelography were used for definitive diagnosis. The presence of urinary tract symptoms or a history of urolithiasis may serve as clinical clues in patients with acute LBP [437].

**Table 25:** Urinary kidney stones and hydronephrosis causing low back pain

**Author / year / country**	**Design / setting**	**Patient / diagnosis**	**Spinal symptoms**	**Extraspinal symptoms**	**Diagnostic confirmation**
Nakamura et al., 2002, Japan [437]	CR / ambulatory care	female, 32 y / kidney stone	acute LBP, right sided	cloudiness of urine	pyelography
Mantle et al., 2003, UK [26]	CR / not reported	male, 47 y / hydronephrosis secondary to ureteral hernia	chronic LBP with exacerbation, radiation to the left loin	-	CT
Tieppo Francio et al., 2018, USA [33]	CR / outpatient clinic	male, 60 y / hydronephrosis	chronic LBP with exacerbation, radicular, antalgic gait, paraesthesia, mild limp	-	CT

*CR* case report*, CT* computed tomography*, LBP* low back pain*, y* years

Hydronephrosis is a disease characterised by renal pelvis expansion resulting from obstructed urinary outflow and subsequent retention. In most cases, it is caused by urinary kidney stones. Two case reports were identified, showing an association between LBP and hydronephrosis (Table 25). The reports detailed exacerbated and chronic LBP, accompanied by neurological symptoms like radicular pain, paraesthesia, and mild limping [33]. Otherwise, there were no other clinical signs or symptoms implicating hydronephrosis as an underlying cause of LBP. Diagnosis frequently occurs incidentally, prompted by imaging studies ordered for evaluation of neurological symptoms.

#### Prostatic diseases

Prostatic diseases (including prostatitis, prostatic calculi, and cysts) can also present with acute LBP. Five relevant case reports (Table 26) on the subject were identified. Prostatitis can manifest in young males, while prostatic calculi predominantly occur in older male individuals. Clinical clues that point to prostatic pathology as underlying cause for acute LBP are symptoms of urinary tract infections [438], fever, and occasional incontinence [439].

**Table 26:** Prostatic diseases presenting with low back pain

**Author / year / country**	**Design / setting**	**Patient / diagnosis**	**Spinal symptoms**	**Extraspinal symptoms**	**Diagnostic confirmation**
Bajaj et al., 2008, USA [439]	CR / hospital	male, 66 y / bilateral prostatitis	acute LBP	fever, chills, decreasing renal function, frequency, urgency, and occasional incontinence	SPECT-CT
Godara et al., 2003, India [438]	CR / not reported	male, 50 y / prostatic calculi	acute LBP	symptoms of urinary tract infection	surgery and histopathological examination
Qiu et al., 2018, China [440]	CR / hospital	male, 24 y / prostatic cyst	acute LBP, left-sided	-	abdominal ultrasonography
Rau et al., 2018, Switzerland [441]	CR / ambulatory care	male, 74 y / prostatitis	acute + chronic LBP, radiating in buttock, groins, and thighs; immobility	-	laboratory
Mateos et al., 2003, Spain [442]	CR / not reported	male, 57 y / prostatitis + orchiepididymitis	bilateral acute LBP	fever, chills, perineal pain	ultrasonography + scintigraphy

*CR* case report*, LBP* low back pain*, SPECT-CT* single photon emission computed tomography – computed tomography*, y* years

#### Renal infarction

Renal infarction can result from an embolism entering the renal vein or artery. A case report documenting LBP as part of the clinical presentation was found (Table 27). Accompanying symptoms were vomiting and chest discomfort. There may be abnormalities seen in the blood test, such as elevated troponin I levels [443, 444].

**Table 27:** Low back pain in association with renal infarction

**Author / year / country**	**Design / setting**	**Patient**	**Spinal symptoms**	**Extraspinal symptoms**	**Diagnostic confirmation**
Cahill et al., 2012, UK [443]	CR / not reported	male, 44 y	acute LBP	vomiting, exertional chest discomfort	arterial phase contrast enhanced CT of abdomen

*CR* case report*, CT* computed tomography*, LBP* low back pain*, y* years

#### Renal ischaemia

Renal ischaemia is a rare condition that can result from various causes, for example, exercising. A case report outlining acute LBP within the context of renal ischaemia was identified (Table 28). Additional symptoms encompassed nausea, vomiting, and abdominal pain [443, 444].

**Table 28:** Low back pain in association with renal ischaemia

**Author / year / country**	**Design / setting**	**Patient**	**Spinal symptoms**	**Extraspinal symptoms**	**Diagnostic confirmation**
Maekawa et al., 2017, Japan [444]	CR / ED	male, 26 y	acute LBP, myalgia in lower extremities	abdominal pain, severe nausea and vomiting, low grade fever	CT and renal biopsy

*CR* case report, *CT* computed tomography, *ED* emergency department*, LBP* low back pain*, y* years

#### Infected kidney cysts

Kidney cysts, fluid-filled sacs found in kidneys, can vary in size and can be solitary or multiple. In total, three case reports documented instances of acute LBP associated with infected kidney cysts (Table 29). In patients presenting with acute LBP along with symptoms like fever and vomiting [445], kidney cysts should be considered as differential diagnosis. Furthermore, if the patient’s past medical history includes previous kidney diseases, this could serve as a valuable clinical clue.

**Table 29:** Infected kidney cysts presenting with low back pain

**Author / year/ country**	**Design / setting**	**Patient**	**Spinal symptoms**	**Extraspinal symptoms**	**Diagnostic confirmation**
Ito et al., 2016, Japan [445]	CR / hospital	male, 58 y	acute LBP	fever, vomiting, and feeling of abdominal enlargement	blood culture and CT
Jensen et al., 2020, Denmark [446]	CR / hospital	female, 74 y	severe acute LBP (NRS: 9/10)	low grade fever	blood culture and PET/CT
Mandai et al., 2014, Japan [447]	CR / hospital	male, 48 y	acute LBP, right sided	fever, arthralgia, anorexia	MRI + laboratory

*CR* case report, *CT* computed tomography, *LBP* low back pain, *MRI* magnetic resonance imaging*, NRS* numeric rating scale, *PET* positron emission tomography*, y* years

### Low back pain

Multiple narrative reviews and one systematic review were identified (Table 30). Major differences emerged regarding the classification of LBP causes and terminology. The most commonly proposed classification systems are based on either mechanical/non-mechanical [3–6, 8, 10, 12, 448–450] or specific/non-specific [37, 451–454] causes of LBP. In certain instances, degenerative diseases [455, 456] or radiculopathy [12, 13, 16, 449, 453–455, 457–459] were also designated as major categories. Several classification systems further isolate extravertebral diseases as a distinct class [3–6, 8, 10, 12, 13, 16, 37, 448–450, 454–461]. Various terms have emerged to describe these extravertebral causes, such as visceral diseases, non-spinal diagnoses or aetiologies, medical causes, and referred pain. The lack of a clear definition of extravertebral LBP was also reflected in the listing of various diseases according to the classification system. For example, intestinal infections were included in non-mechanical LBP [4] despite explicit classification of them as an extravertebral cause. Many classification systems adopt the concept of red flags as indications of specific LBP, which generally do not explicitly cover extravertebral pathologies. The most frequently referenced publication regarding extravertebral pathologies was the work by Deyo and Weinstein in 2001 [3]. They estimated a prevalence of 2% for extravertebral LBP without specifying the clinical setting, such as ambulatory care versus emergency room, or providing a data source for this assumption. However, recent research studies have found disparities from this estimate with prevalences ranging from <1% [458] to 10% [8], which can be explained by the assortment of pathologies grouped within the extravertebral LBP category.

**Table 30:** Publications of low back pain in general

**Author / year / country**	**Design**	**Classification**	**Extravertebral causes of LBP**	**Reported prevalence**	**Citation of Deyo and Weinstein 2001**
Deyo et al., 2001, USA [3]	NR	mechanical LBP and leg pain, nonmechanical spinal conditions, visceral disease	prostatitis, endometriosis, chronic pelvic inflammatory disease, nephrolithiasis, pyelonephritis, perinephric abscess, aortic aneurysm, pancreatitis, cholecystitis, penetrating ulcer	mechanical: 97%, nonmechanical: 1%, visceral: 2%	-
Müller et al., 2001, Germany [4]	NR	mechanical, non-mechanical, visceral	visceral: prostatitis, endometriosis, nephrolithiasis, pyelonephritis, perinephric abscess, aortic aneurysm, pancreatitis, pancreas carcinoma, cholecystitis, penetrating ulcer; non-mechanical: epidural abscess, herpes zoster, intestinal infections	2%	yes
Atlas et al., 2001, USA [448]	NR	mechanical LBP, nonmechanical LBP, visceral disease	prostatitis, endometriosis, chronic pelvic inflammatory disease, nephrolithiasis, pyelonephritis, perinephric abscess, AAA, aortoiliac disease, pancreatitis, cholecystitis, perforated bowel	not reported	no
Hicks et al., 2002, USA [5]	NR	mechanical diseases, nonmechanical spinal diseases, visceral diseases	nephrolithiasis, pyelonephritis, prostatitis, pelvic inflammatory disease, endometriosis, AAA	< 2% (caused by malignancy, infection, or visceral cause)	yes
Zimmermann et al., 2002, USA [462]	NR	“referred pain”	-	not reported	no
Jarvik et al., 2002, USA [6]	NR	mechanical LBP or leg pain, nonmechanical spinal conditions, visceral disease	pelvic organ involvement (prostatitis, endometriosis, chronic pelvic inflammatory disease), renal involvement (nephrolithiasis, pyelonephritis, perinephric abscess), aortic aneurysm, gastrointestinal involvement (pancreatitis, cholecystitis, penetrating ulcer)	2%	yes
Carragee et al., 2004, USA [450]	NR	mechanical, nonmechanical, visceral disease	nephrolithiasis, prostatitis, pelvic inflammatory disease	2% (malignancies, infection, visceral causes and other “red herrings”)	no
Devereaux et al., 2004, USA [7]	NR	not reported	dissecting aortic aneurysm, renal colic, tumours of multiple abdominal organs	not reported	yes
Stowell et al., 2005, Lebanon [463]	NR	not reported	AAA, gynaecological diseases (i.e., endometriosis, pelvic inflammatory disease, ovarian cyst), gastrointestinal infection (i.e., peritonitis, appendicitis, pancreatitis), renal disorders (i.e., nephrolithiasis, pyelonephritis, UTI), intestinal obstruction	not reported	no
Diamond et al., 2006, USA [8]	NR	mechanical LBP, nonmechanical LBP, visceral disease	prostatitis, endometriosis, chronic pelvic inflammatory disease, nephrolithiasis, pyelonephritis, perinephric abscess, aortic aneurysm, pancreatitis, cholecystitis, penetrating ulcer	mechanical disorders: 90%, manifestation of systemic illness: 10%	yes
Winters et al., 2006, USA [9]	NR	not reported	cardiovascular, pulmonary, gastrointestinal, and urogenital system disease	not reported	yes
Klineberg et al., 2007, USA [464]	NR	not reported	dissecting aortic aneurysm, ectopic pregnancy, myocardial infarction, acute pancreatitis, duodenal ulcers, pyelonephritis, visceral trauma, cholecystolithiasis, endometriosis, fibroids, nephrolithiasis, pelvic inflammatory disease, pregnancy, prostatitis, UTI	not reported	no
Ponka et al., 2007, Canada [465]	NR	not reported	pelvic infection, nephrolithiasis, pancreatic disease and AAA, UTI, prostate cancer	not reported	no
Weiland et al., 2007, Germany [466]	NR	not reported	aortic bifurcation syndrome, aortic aneurysm, zoster radiculitis, nephrolithiasis, pancreatitis, ruptured aortic aneurysm	not reported	no
Kinkade et al., 2007, USA [10]	NR	mechanical LBP, nonmechanical spinal conditions, non-spinal / visceral disease	pelvic organs (prostatitis, pelvic inflammatory disease, endometriosis), renal organs (nephrolithiasis, pyelonephritis), aortic aneurysm, gastrointestinal system (pancreatitis, cholecystitis, peptic ulcer), shingles	2%	yes
Graw et al., 2008, USA [460]	NR	spinal diagnoses, non-spinal diagnoses	neoplasm, infection, systemic medical conditions, intrapelvic, gynaecological conditions, renal disease, AAA, sacroiliac joint dysfunction, hip pathology, diabetic neuropathy	not reported	yes
Cohen et al., 2008, USA [449]	NR	mechanical, neurogenic, nonmechanical, referred visceral pain, other	gastrointestinal diseases (inflammatory bowel disease, pancreatitis, diverticulitis), renal disease (nephrolithiasis, pyelonephritis), abdominal aortic aneurysm	1-2%	yes
Ludwig et al., 2010, Germany [461]	NR	vertebral & extravertebral causes of LBP	visceral diseases, gynaecological diseases, urologic diseases	not reported	not reported
Dagenais et al., 2010, USA [467]	SR	not reported, specific causes of LBP	aortic aneurysm, enteropathic disease, endocarditis, nephrolithiasis, or pancreatitis	not reported	no
Miura et al., 2011, Japan [11]	NR	not reported	aortic disease (dissection aortic aneurysm, true aortic aneurysm), oesophageal rupture, pancreatitis, cholecystitis and cholelithiasis, kidney and urinary tract disease	not reported	yes
Manusov et al., 2012, USA [12]	NR	mechanical, non-mechanical and visceral LBP; or: nonspecific LBP, radicular back pain and worrisome or medical red flags	prostatitis, endometriosis, chronic pelvic inflammatory disease, nephrolithiasis, pyelonephritis, perinephric abscess, aortic aneurysm, pancreatitis, cholecystitis, penetrating ulcer	2%	yes
Schulte-Mattler et al., 2013, Germany [456]	NR	idiopathic back pain, degenerative process of intervertebral discs or facet joints, mechanical spinal causes, nonmechanical spinal causes, visceral causes	prostatitis, endometriosis, nephrolithiasis, pyelonephritis, perinephric abscess, aortic aneurysm, pancreatitis, cholecystitis, penetrating ulcer, pregnancy; non-mechanical spinal causes: epidural abscess, zoster	2%	not reported
Amirdelfan et al., 2014, USA [459]	NR	structural aetiologies of LBP, neurogenic aetiologies of LBP, extraspinal aetiologies	rheumatological conditions (rheumatoid arthritis, ankylosing spondylitis, ossification of posterior longitudinal ligament, Paget’s disease, osteoporosis, Reiter's syndrome, psoriatic spondylitis, polymyalgia rheumatica); renal and urologic conditions (obstructive (stones, prostate), inflammatory, neoplastic, infectious (pyelonephritis)); gastrointestinal conditions (ischaemic bowel disease, motility disorders, gastrointestinal inflammation, gastrointestinal ulceration and perforation, gallbladder disease, diverticulitis, appendicitis (retrocecal), pancreatitis); pelvic and gynaecological disorders (pregnancy, menstruation, ovarian cysts, endometriosis, uterine fibroids, pelvic adhesions, ectopic pregnancy, foreign body, inflammatory pelvic disorders); cardiovascular disorders (vascular insufficiency, superior mesenteric artery syndrome, renal artery stenosis, venous obstruction, AAA, endocarditis, aortoiliac disease); infection (discitis, osteomyelitis, Pott's disease, postherpetic neuralgia); neoplasm (neurospinal aetiology, abdominal aetiology, metastatic); psychological (hypochondria, somatoform disorder, factitious disorder, secondary gain issues, malingering)	not reported	no
Golob et al., 2014, USA [13]	NR	nonspecific “mechanical”, back pain with lower extremity symptoms, systemic and visceral diseases	prostatitis, endometriosis, chronic pelvic inflammatory disease, nephrolithiasis, pyelonephritis, perinephric abscess, aortic aneurysm, pancreatitis, cholecystitis, penetrating ulcer	serious systemic pathologic condition: 5%	yes
Jones et al., 2014, UK [457]	NR	simple mechanical LBP, LBP with radiculopathy, serious pathological LBP, visceral disease masquerading as spine pathology	AAA, duodenal ulceration, cholecystolithiasis, nephrolithiasis, prostatitis, UTI, fibroids	2%	no
Melcher et al., 2014, Germany [452]	continuous medical education	nonspecific (includes non-vertebral), specific	AAA, iliac artery occlusion, pancreatitis, urolithiasis, pyelonephritis, psoas abscess, psoas haematoma, pelvic mass, fracture of the femoral neck, coxitis, trochanteric bursitis, piriformis syndrome, polyneuropathy	not reported	no
Patrick et al., 2014, USA [453]	NR	nonspecific LBP, back pain associated with radiculopathy or spinal stenosis, back pain associated with a specific spinal cause	nephrolithiasis, pyelonephritis, prostatitis, endometriosis, ovarian cysts, esophagitis, gastric and peptic ulcer disease, cholelithiasis and cholecystitis, pancreatitis, diverticulitis, other intra-abdominal infection, abdominal or thoracic aortic aneurysm, cardiac ischaemia or myocardial infarction, intramedullary spinal cord tumours	not reported	no
Chou et al., 2014, USA [454]	NR	nonspecific LBP, back pain potentially associated with radiculopathy or spinal stenosis, back pain potentially associated with another specific systemic or spinal cause	intra-abdominal visceral disease: gastrointestinal (peptic ulcer or pancreatitis), genitourinary (nephrolithiasis, pyelonephritis, prostatitis, pelvic infection, or tumour), vascular (aortic dissection)	not reported	no
Hooten et al., 2015, USA [14]	NR	medical (non-musculoskeletal), musculoskeletal	neoplastic (metastatic carcinoma, multiple myeloma, lymphoma, leukaemia, spinal cord tumours), inflammatory (ankylosing spondylitis, psoriatic spondylitis, rheumatoid arthritis, Reiter’s syndrome, enteropathic spondylitis), visceral (endometriosis, prostatitis, nephrolithiasis, aortic aneurysm, pancreatitis), infectious (osteomyelitis, epidural abscess, discitis, herpes zoster, pyelonephritis), vascular (aortic aneurysm, aortic dissection, spinal haemangioma, inferior vena cava obstruction, sickle cell crisis), endocrine (osteoporotic fracture, Paget’s disease), traumatic (vertebral fracture, rib fracture, pelvic fracture, hip fracture)	not reported	yes
Selkirk et al., 2016, USA [15]	NR	mechanical back pain, nonspecific LBP, referred pain	prostatitis, pancreatic disease, gallbladder disease, aortic aneurysm, pyelonephritis; infectious causes: epidural abscess, paraspinous abscess, herpes zoster, varicella zoster; causes of radiculopathy and radicular pain: piriformis syndrome, diabetic amyotrophy	not reported	yes
Singleton et al., 2016, USA [16]	NR	benign, self-limited musculoskeletal causes, spinal pathologies that can cause severe neurologic disability, other abdominal or retroperitoneal processes that can present with back pain	aortic diseases (aneurysm, dissection, ulceration and aortitis), genitourinary disease (ureteral colic, renal infarction and tumour, prostatitis), gastrointestinal causes (pancreatitis and pancreatic cancer, penetrating peptic ulcer, cholecystitis and cholangitis), retroperitoneal haematoma, systemic infections including endocarditis, psoas abscess and other localized abscess	not reported	yes
Verhagen et al., 2016, The Netherlands [468]	NR	not reported	aneurysm	not reported	no
Maher et al., 2017, Australia [451]	NR	specific, non-specific LBP	-	2%	yes
Vlaeyen et al., 2018, Belgium [458]	NR	visceral disorder, specific spinal disease, radicular syndromes or nonspecific LBP	-	< 1% (caused by visceral or spinal disease)	yes
Chenot, 2018, Germany [37]	NR	specific, non-specific, extravertebral	cardiac ischaemia, aortic aneurysm, pyelonephritis, nephrolithiasis, ischaemia / thrombosis of intraabdominal organs, iliosacral joint discomfort, gynaecological / urological diseases, cholelithiasis, pancreatitis, penetrating ulcer, nephrolithiasis	2%	no
Reith et al., 2020, Germany [455]	NR	unspecific / degenerative, radicular, acute vertebral / extravertebral	-	unspecific degenerative: 85-90%, radicular: 1-2%, acute vertebral / extravertebral: <10%	no

*AAA* abdominal aortic aneurysm*, LBP* low back pain*, NR* narrative review, *SR* systematic review, *UTI* urinary tract infection

Solely a singular case series involving 95 patients (34.7% female) from Japan presenting themselves at an emergency department (ED) with LBP [469] was identified. Within this group, a total of 66.2% were diagnosed to have a urological disease. Other reported disorders were vascular diseases like AAA or aortic dissection and pancreatitis. In most cases the diagnosis was confirmed by using a CT. However, given that primary care services in Japan are frequently attached to hospitals, the generalisability of this finding is limited.

### Miscellaneous

#### Spinal epidural lipomatosis

Spinal epidural lipomatosis is a rare condition characterised by excessive proliferation of adipose tissue within the epidural space leading to spinal canal stenosis. In total, nine case reports, one case series, one cohort study and one narrative review reporting LBP associated with spinal epidural lipomatosis were identified (Table 31). Predominantly, this condition manifested in middle-aged and older male individuals. An association with elevated body mass index (BMI), previous treatment with corticosteroids or other endocrinological disorders, e.g., Cushing syndrome, is postulated [470]. Due to neurological symptoms, such as limb weakness or numbness [470–473], an MRI was usually performed, ultimately confirming the diagnosis.

**Table 31:** Spinal epidural lipomatosis presenting with low back pain^a^

**Author / year / country**	**Design / setting**	**Patient(s)**	**Spinal symptoms**	**Diagnostic confirmation**
Chan et al., 2009, Taiwan [471]	CR / hospital outpatient depart-ment	male, 56 y, BMI: 24.1 kg/m^2^	chronic LBP (sustained, worsening), left leg weakness and numbness, inability to walk long distances	MRI
		male, 44 y, BMI 23.4 kg/m^2^	chronic LBP (progressive worsening, inability to walk for long distances), left extremity weakness and numbness	MRI
Duran et al., 2016, Turkey [474]	CR / not reported	female, 46 y, BMI: 32 kg/m^2^	chronic LBP (exacerbating over three months, deep, sharp), radicular pain in the proximal leg	MRI
Min et al., 2007, South Korea [475]	CR / not reported	male, 70 y, BMI: normal	chronic LBP (VAS 8/10, aggravating by standing and walking, relieved by sitting and leaning forward during walking), radiating to both lower limbs.	MRI
McCormick et al., 2014, USA [476]	CR / hospital	male, 79 y, BMI: 42.1 kg/m^2^	chronic LBP (progressive, VAS 8/10, stabbing, relieved by sustained lumbar flexion positions and exacerbated by walking or standing), radiation to the right anterolateral thigh	MRI
		male, 47 y, BMI: 32.5 kg/m^2^	chronic LBP (VAS 9/10, burning and tingling, exacerbated by sitting, standing, and walking), radiation to anterolateral thigh and lateral posterior leg	MRI
		male, 50 y, BMI: 48 kg/m^2^	acute LBP (severe, VAS 10/10, achy and numb, began upon standing from a seated position, patient felt a “pop”, exacerbated by standing, walking, and ascending stairs)	MRI
Al-Khawaja et al., 2008, Australia [473]	CR / not reported	male, 68 y, BMI: not reported	chronic LBP (aggravating by standing longer than 10 minutes or walking greater than 200 m), claudication right anterior thigh and lateral lower leg	MRI
Botwin et al., 2004, USA [477]	CR / not reported	female, 78 y, BMI: 28.2 kg/m^2^	chronic LBP (VAS 8/10, aggravated by standing and walking, relieved by sitting and leaning forward), radiation to the left lower limb	MRI
		male, 68 y, BMI: 32.6 kg/m^2^	chronic LBP (VAS 8/10, aggravated by standing and walking, relieved by sitting and leaning forward during walking), radiation to the right lower limb	MRI
Choi et al., 2012, Korea [478]	CR / not reported	male, 67 y, BMI: 25.5 kg/m^2^	back pain (no duration or localisation reported), leg tightness in both legs, neurogenic claudication	MRI, MR myelogram
Lisai et al., 2001, Italy [479]	CR / not reported	male, 67 y, BMI: not reported	chronic LBP, bilateral pain radiating to his feet	CT / MRI
		male, 54 y, BMI: not reported	chronic LBP, radiating pain in both legs and intermittent sacral nerve dysfunction including penile erection, incontinence, perineal pain	MRI
Maillot et al., 2006, France [480]	CR / hospital	male, 63 y, BMI: 32.6 kg/m^2^	chronic LBP (diurnal on standing and walking, walking limited to 200 m), pain in lower limbs, radicular pain in the left calf	CT myelography
Borré et al., 2003, Argentina [470]	CS / hospital	2528 (57% female), mean age: 47.3 y	non-specific LBP (no duration reported) (LEL grade I: 0%; LEL grade II: up to 14.5%; LEL grade III: up to 100%); radicular pain, numbness and dysaesthesias, neurogenic claudication, cauda equina syndrome; LEL grade I: no symptomatic patients; LEL grade II: 14.5% were symptomatic with weakness and / or dysaesthesia (no other substantial pathologic findings in MRIs); LEL grade III: 100% were symptomatic, 42.3% showed other pathological findings	-
Ishikawa et al., 2006, Japan [472]	CHS / hospital	7 (29% female), mean age: 65.7 y, mean BMI: 27.1 kg/m^2^	LBP (queried) (no duration reported), neurological deficits in the lower extremities, with intermittent claudication as seen in cauda equina syndrome	-
Al-Khawaja et al., 2008, Australia [473]	NR / -	111 cases: *44% idiopathic:* 10% female, 70% obese, mean age: 46 y *56% secondary:* 20% female, 70% obese, mean age: 44 y	*idiopathic lumbar lipomatosis:* 65% back (no duration or localisation reported) or leg pain, 30% leg weakness, 30% claudication; *secondary lumbar lipomatosis:* 50% back (no duration or localisation reported) or leg pain, 25% leg weakness, 10% claudication	-

^a^in all publications no extraspinal symptoms were reported

*BMI* body mass index*, CHS* cohort study, *CR* case report, *CS* case series*, CT* computed tomography*, LBP* low back pain, *LEL* lumbosacral epidural lipomatosis*, MR* magnetic resonance*, MRI* magnetic resonance imaging*, NR* narrative review*, VAS* visual analogue scale*, y* years

#### Episacral lipoma – Back mice

Back mice are subfascial fat herniations in the back. They are often accompanied by painful swellings, but one case report showed that they can also cause LBP (Table 32). Ultrasound examination is used to confirm the diagnosis.

**Table 32:** Case reports of back mice presenting with low back pain

**Author / year / country**	**Setting**	**Patient**	**Symptoms**	**Diagnostic confirmation**
Tiegs-Heiden et al., 2018, USA [481]	hospital	female, 47 y	chronic LBP (intermittent, lasting about a week at a time and then remitting), palpable mass in the right lower back	ultrasound

*LBP* low back pain,* y* years

#### Hip pathology

Hip and lumbar spine pathologies often occur in combination and may be difficult to separate [482–484]. Pathologies of the lower back, e.g. in the iliosacral joint, or radicular symptoms can present predominantly with hip pain or pain in the thigh. Hip pain was frequently reported in the case reports reviewed, e.g. in systemic diseases affecting the hip and facet joints [88, 91, 92], but it was also part of the presentation in cases involving other organs [23, 380]. On the other hand, hip pathology can lead to LBP. An observational study of 25 patients (32-84 years) with hip and spinal pain showed improvement of back pain following total hip replacement [482]. Limited range of motion of the hip has been observed in patients with chronic LBP compared to healthy individuals, with improvement noted following hip exercise [485]. This is in line with other studies and a recent systematic review, despite low certainty evidence that hip strenghtening can improve LBP [486]. Examination of the hip (forced internal rotation) should be part of the clinical examination in patients presenting with pain radiating into the thigh.

### Summary of clinical clues for the diagnosis of extravertebral LBP (Table 33)

**Table 33:** Summary of clinical clues for the diagnosis of extravertebral low back pain

**Disease**	**Patient characteristics**	**Acute / chronic LBP**	**Clinical clue / symptoms**	**Diagnostic**
***Systemic diseases***				
Gout	male > female, older age	acute and chronic	h/o gout, tophaceous gout	dual energy CT
Pseudogout	older age	mostly subacute or chronic	-	biopsy during surgery
Skeletal fluorosis	endemic environmental, industrial and accidental exposure to fluoride	chronic, progressive	neurological manifestation (e.g., paraparesis), mottling teeth	x-ray
Spinal sarcoidosis	middle age	chronic	h/o sarcoidosis	MRI, bone biopsy
Hyperparathyroidism	female > male	chronic	constitutional symptoms (e.g., weakness), prior history of urolithiasis and ESKD	laboratory assessment, surgery / biopsy
Vitamin D deficiency / insufficiency	female > male, older age (postmenopausal)	chronic	-	laboratory assessment
Ochronosis / alkaptonuria	male > female, middle age	chronic	pigmentation of the sclera and ear and darkening of morning urine, intervertebral disc calcification on imaging	laboratory assessment (measurement of homogentisic acid in the urine)
***Arterial diseases***				
Abdominal aortic aneurysm	middle aged and older, male, cardiovascular risk factors	chronic > acute (depending on type)	pulsating abdominal mass, abdominal pain, progressive exacerbation of symptoms	ultrasound, confirmation: CT, MRI
Acute aortic syndrome (including aortic dissection, intramural haematoma and penetrating aortic ulcer)	middle aged, male	acute	chest discomfort, abdominal pain, nausea / vomiting, dyspnoea	CTA
Fistula (including aorto-enteric, aorto-venous and, aorto-caval fistula)	middle-aged to older, male	acute and chronic	depending on affected structures: abdominal pain, vomiting, neurological symptoms	CT / MRI
Peripheral arterial disease	male	acute and chronic	leg pain	vascular examination, Doppler
***Venous diseases***				
Deep vein thrombosis	younger age	rather acute	leg swelling, radicular symptoms	ultrasound, CT / MRI
Varicosis (epidural, intradural, gluteal)	female > male, any age	acute and chronic	leg pain, neurological symptoms (e.g., paresis)	MRI
***Paraspinal haematoma***				
Subdural, epidural, subarachnoid, other localisations	male > female, middle aged to older age	acute	sudden onset paraparesis, symptoms suggestive of cauda equina syndrome (urinary and faecal incontinence), oral anticoagulation	MRI
***Chronic periaortitis***				
Retroperitoneal fibrosis	male, age: 40-60 years	acute and chronic	constitutional symptoms, symptoms of ureteral obstruction, autoimmune or IgG4-related diseases in patients’ history	CT / MRI + biopsy
Inflammatory abdominal aortic aneurysm	5-10 years younger than patients with AAA	chronic	constitutional symptoms (e.g., fever), abdominal pulsating mass, abdominal pain	CT + biopsy
***Cardiological diseases***				
Myocardial infarction	middle aged to older	acute	chest pain, shock symptoms	CT
***Gastroenterological diseases***				
Gallstone disease / cholecystitis	female > male	acute and chronic	upper abdominal pain	ultrasound, MRI / MRCP
Pancreatitis	-	acute	abdominal pain, appetite loss, jaundice	ERC / biopsy
***Paraspinal compartment syndrome***				
-	male > female, younger or middle aged	acute	heavy exercise	MRI, pressure measurement
***Gynaecological diseases***				
Endometriosis	women of childbearing age	rather chronic	dyspareunia, dysmenorrhoea, cyclical pain	referral to gynaecologist
***Urological diseases***				
UTI / pyelonephritis	female > male, increasing with age	rather acute	malaise, fever	urinary and / or blood culture
Urinary kidney stone / hydronephrosis	male > female, middle aged	acute or exacerbation	haematuria	urine dipstick and ultrasound
Prostatic disease	male, any age	rather acute	urgency, fever	urine dipstick, referral to urologist
Renal infarction/renal ischaemia	male > female, younger or middle aged	rather acute	abdominal pain, nausea and vomiting	ultrasound, CT

*AAA* abdominal aortic aneurysm, *CT* computed tomography, *CTA* computed tomography angiography*, ERC* endoscopic retrograde cholangiography*, ESKD* end stage kidney disease, *h/o* history of*, IgG4* immunoglobulin G4*, MRCP* magnetic resonance cholangiopancreatography*, MRI* magnetic resonance imaging*, UTI* urinary tract infection

## Discussion

### Summary of evidence

It can be difficult to distinguish clinically between vertebral and extravertebral causes of LBP and there is limited research to date. This is further complicated by the wide range of differential diagnoses and the rare incidence of extravertebral disorders that mimic LBP. This scoping review attempts to provide a comprehensive overview of the aetiologies underlying extravertebral LBP, with a particular focus on identifying symptoms indicative of extravertebral pathology. The available body of evidence, largely derived from case reports and retrospective cohort studies, does not allow epidemiological conclusions to be drawn regarding the prevalence of extravertebral LBP. The diagnosis of extravertebral pathology is frequently made incidentally by imaging or intraoperatively. However, as summarised in Table 33**,** this review has identified clinical signs and symptoms that may facilitate the identification of specific aetiologies of extravertebral LBP.

### Interpretation of the results

The large number of case reports highlights the clinical relevance of individuals presenting with LBP ultimately attributed to extravertebral causes. However, the clinical relevance of these reports varies widely, ranging from life-threatening conditions to those of lesser clinical consequence. This variability is not surprising, given the diverse range of extravertebral causes for LBP. Accurately estimating the prevalence of extravertebral LBP proves challenging and most likely depends on the clinical setting. It is reasonable to hypothesise that the prevalence is lower in the primary care setting. Deyo, without providing a specific source, estimated the prevalence at 2 % in primary care [3], a figure that has subsequently been cited in numerous LBP reviews [4, 7–11, 13, 14, 16, 449, 451, 458, 460, 487], albeit this has never been confirmed in an epidemiological study.

Many case reports lack details regarding the clinical setting, but it is reasonable to assume that they predominantly originate from specialist clinics or hospitals, as these comprise the majority of reports. Unfortunately, only a limited number of reports indicated whether patients were referred from primary care and the specific reasons for their referral [54, 137, 211, 229, 263, 274, 279, 297, 374]. It has been suggested that non-mechanical back pain may indicate the possibility of extravertebral LBP [3, 464]. Although this assumption seems plausible, there is currently a lack of empirical evidence to substantiate it. This is consistent with the situation of the “red flags” for specific low back pain, which similarly also lack a solid epidemiological foundation [77, 468, 488].

Retrospective case series focussing on specific pathologies often demonstrate LBP as part of the clinical presentation, as seen, for example, in endometriosis [416, 417] or retroperitoneal fibrosis [294, 300, 304, 320, 322, 323, 326, 327, 330, 333, 334, 353, 361, 362]. The practical utility of these observational studies for the clinician, who frequently encounter LBP, is limited due to the relative rarity of these pathologies in patients presenting with LBP as leading symptom. However, the definitive causal relationship with LBP remains uncertain, and the possibility of coincidence must be considered. Nonetheless, when patients present with an unusual combination of symptoms that deviate from the usual clinical picture, consideration of extravertebral pathologies becomes important. Certain extravertebral pathologies are less likely overlooked, as they present with symptoms such as paraplegia [171, 183, 188, 230, 247, 281], and always warrant advanced imaging or surgical exploration, ultimately leading to the correct diagnosis.

Case reports frequently conclude that especially primary care providers should remain vigilant for specific pathologies in patients presenting with LBP. However, given that LBP is a common reason for consultation and the majority of patients with low back pain do not exhibit serious underlying vertebral or extravertebral pathology, this approach appears impractical. The extensive examinations and test required to cover all potential differentials would exceed what is feasible and reasonable. Therefore, a primary care strategy of watchful waiting seems appropriate in the absence of serious symptoms necessitating urgent investigation.

This scoping review identified two categories of extravertebral back pain, classifying them based on symptoms that led to incidental diagnoses or cases with symptoms prompting clinical suspicion of non-vertebral pathology, such as a pulsatile swelling and AAA [101, 102, 104, 106–110, 115, 127, 131, 147, 149]. This differentiation holds forensic implications when patients litigate against health professional for misdiagnosis. Negligence can only be assumed if the clinical presentation rendered an extravertebral cause of LBP reasonably probable.

The majority of clinical guidelines [1] did not explicitly recommend considering extravertebral causes in the evaluation of low back pain. Based on the findings of this scoping review, it is recommended that the clinical assessment of LBP should incorporate a brief consideration of possible extravertebral causes as a measure to improve patient safety.

### Comparison with existing literature

There is limited comprehensive literature on the topic, with the majority of reviews on red flags focussing on spinal pathologies, neglecting extraspinal pathologies [36, 468, 487, 488]. Reviews addressing extravertebral causes tend to be narrative in nature, lacking a systematic literature search dedicated to extravertebral causes [3, 7–11, 13, 14, 37, 449, 451, 458, 460]. Siddiq et al. conducted a focused systematic review on differential diagnosis of sciatica, emphasising musculoskeletal causes, which incidentally identified a few extraspinal causes of sciatica [489]. Maselli et al. provided a more targeted review of red flags in thoracolumbar pain, highlighting myocardial infarction, reflux, and pulmonary disease as important differential diagnosis [35]. Our review, focused on lumbar pain, excluded thoracic pain and treatment complications. The clinical clues for extravertebral LBP identified in our review (Table 33) exhibit limited reliability, akin to “red flags” for spinal pathologies [35, 36, 468, 487, 488].

### Strength and limitations

To the best of current knowledge, this scoping review is the first attempt to comprehensively evaluate extravertebral LBP and provide an overview of associated clinical clues (Table 33). A detailed review addressing extravertebral causes that mimic radicular pain, encompassing infectious pathologies and post-injection complications, has already been published and was consequently excluded from this scoping review [489].

### Limitations of the review

Clear decisions regarding the inclusion or exclusion of case reports often presented challenges, particularly in distinguishing between “red flag” pathologies and extravertebral diseases. Furthermore, the pragmatic decision to exclude cancers, metastases, and benign tumours from this review was made due to the extensive spectrum of pathologies involved.

Determining whether LBP was the primary complaint in the clinical presentation and differentiating it from related neurological diseases and leg pain was often impossible based on the publications. Despite the focus on low back pain, precise descriptions of the localisation of back pain were frequently absent. Furthermore, given low back pain’s high prevalence and chronic nature, it was not always feasible to differentiate between LBP as an independent condition or as part of the clinical manifestation of an underlying disease. As a result, some of the exclusion decisions may seem arbitrary. Moreover, not all reports provided details on the outcomes following specific interventions.

Complications from prior surgeries, pharmaceutical treatments, invasive or local procedures associated with LBP were excluded, as it was assumed that clinicians assessing the presenting complaint would consider these aspects. It is worth noting that this review’s search was limited to English and German only, therefore potentially introducing language bias. However, we assume that missing some case reports or case series published in other languages would have minimal impact on the scope of the review. Including those case reports and case series would not allow for a better inference on the epidemiology of extravertebral LBP.

### Limitations of the studies

In numerous studies, LBP was mentioned as part of the clinical presentation; however, it was not consistently stated how the reported presence of LBP related to accompanying symptoms. The description of back pain was often inadequate in terms of location (lumbar versus thoracic or cervical pain), duration, and other important clinical circumstances, such as movement-related (mechanical) pain. Although a standard for reporting case reports has been issued [490], we did not attempt a formal quality assessment of the case reports or case series, since the majority of them did not meet the standard set and many of them where published before the CARE guidelines where established.

Some retrospective cohort studies examined the presence of LBP in patients with an established diagnosis, e.g., aortic dissection. From the perspective of clinicians evaluating patients with LBP, this information is of limited value given the epidemiological aspects of LBP. No prospective studies investigating the epidemiology of extra-vertebral pathologies presenting with LBP were found.

## Conclusion

The differential diagnosis of extravertebral LBP is extensive. However, it is reasonable to assume that the prevalence is relatively low and varies depending on the clinical setting. It is essential that a distinction is made between two categories of extravertebral LBP: cases where clinical presentation indicates a likely extravertebral cause, and those where extravertebral LBP is diagnosed incidentally, such as through advanced imaging or intraoperatively.

### Implication for practice

Extravertebral LBP is often diagnosed incidentally in the absence of symptoms indicative of extravertebral pathologies. However, this review identified symptoms suggestive of possible extravertebral LBP, yet the association is predominantly weak and lacks reliable quantification. Clinicians should therefore consider potential extravertebral causes when evaluating patients with LBP, particularly in instances where LBP appears in combination with atypical symptoms such as abdominal pain or leg swelling, or in patients with demographic characteristics (age and sex) predisposing to specific pathologies.

### Implication for research

Given the diverse aetiology and rarity of extravertebral LBP, it is unlikely that more reliable data on its prevalence and presentation will emerge, particularly in primary care settings. In such settings, the prevalence of serious spinal pathologies is already low, and that of extravertebral ones is likely even lower. Therefore, specific settings within specialist care may be more conducive to systematic evaluations for extravertebral LBP. Prospective studies should prioritise reporting on non-spinal pathologies presenting with low back pain. Additionally, case reports and case series should offer a more comprehensive basis for investigating LBP. Moreover, more focused reviews targeting specific pathologies could enhance guidance for clinicians in identifying when to suspect a particular pathology.

### Implication for clinical guideline makers

Guidelines on low back pain should address extravertebral causes of LBP beyond the conventional spinal pathologies typically highlighted by “red flags”. Moreover, achieving consensus on the terminology used to denote such causes is imperative. The integration of an evaluative step within the treatment algorithm of LBP guidelines, tailored to assess the potential for extravertebral LBP, could help to improve recognition and management for these conditions.

## Data Availability

The datasets used and/or analysed during the current study are available from the corresponding author on reasonable request.

## Appendix 1

**Table 34:** Preferred Reporting Items for Systematic reviews and Meta-Analyses extension for Scoping Reviews (PRISMA-ScR) Checklist

**Section**	**Item**	**Prisma-scr checklist item**	**Reported on page #**
**Title**			
Title	1	Identify the report as a scoping review.	1
**Abstract**			
Structured summary	2	Provide a structured summary that includes (as applicable): background, objectives, eligibility criteria, sources of evidence, charting methods, results, and conclusions that relate to the review questions and objectives.	2-3
**Introduction**			
Rationale	3	Describe the rationale for the review in the context of what is already known. Explain why the review questions/objectives lend themselves to a scoping review approach.	3-4
Objectives	4	Provide an explicit statement of the questions and objectives being addressed with reference to their key elements (e.g., population or participants, concepts, and context) or other relevant key elements used to conceptualize the review questions and/or objectives.	4
**Methods**			
Protocol and registration	5	Indicate whether a review protocol exists; state if and where it can be accessed (e.g., a Web address); and if available, provide registration information, including the registration number.	Not applicable. PROSPERO does not register scoping reviews
Eligibility criteria	6	Specify characteristics of the sources of evidence used as eligibility criteria (e.g., years considered, language, and publication status), and provide a rationale.	5
Information sources*	7	Describe all information sources in the search (e.g., databases with dates of coverage and contact with authors to identify additional sources), as well as the date the most recent search was executed.	5
Search	8	Present the full electronic search strategy for at least 1 database, including any limits used, such that it could be repeated.	86-87
Selection of sources of evidence†	9	State the process for selecting sources of evidence (i.e., screening and eligibility) included in the scoping review.	5
Data charting process‡	10	Describe the methods of charting data from the included sources of evidence (e.g., calibrated forms or forms that have been tested by the team before their use, and whether data charting was done independently or in duplicate) and any processes for obtaining and confirming data from investigators.	6
Data items	11	List and define all variables for which data were sought and any assumptions and simplifications made.	6
Critical appraisal of individual sources of evidence§	12	If done, provide a rationale for conducting a critical appraisal of included sources of evidence; describe the methods used and how this information was used in any data synthesis (if appropriate).	Most case reports were published before CARE was released
Synthesis of results	13	Describe the methods of handling and summarizing the data that were charted.	Not applicable
**Results**			
Selection of sources of evidence	14	Give numbers of sources of evidence screened, assessed for eligibility, and included in the review, with reasons for exclusions at each stage, ideally using a flow diagram.	5
Characteristics of sources of evidence	15	For each source of evidence, present characteristics for which data were charted and provide the citations.	Not applicable
Critical appraisal within sources of evidence	16	If done, present data on critical appraisal of included sources of evidence (see item 12).	Not applicable
Results of individual sources of evidence	17	For each included source of evidence, present the relevant data that were charted that relate to the review questions and objectives.	9-40 (+ tables page 90-128)
Synthesis of results	18	Summarize and/or present the charting results as they relate to the review questions and objectives.	40-42
**Discussion**			
Summary of evidence	19	Summarize the main results (including an overview of concepts, themes, and types of evidence available), link to the review questions and objectives, and consider the relevance to key groups.	42-44
Limitations	20	Discuss the limitations of the scoping review process.	45-47
Conclusions	21	Provide a general interpretation of the results with respect to the review questions and objectives, as well as potential implications and/or next steps.	47-48
**Funding**			
Funding	22	Describe sources of funding for the included sources of evidence, as well as sources of funding for the scoping review. Describe the role of the funders of the scoping review.	49

## Appendix 2

**Table 35:** MeSH-terms for MEDLINE, Embase and Cochrane Library

**MeSH-Terms MEDLINE and Embase**
uncommon OR unusual OR rare OR medical condition AND cause* AND low* AND back pain NOT fract* NOT child* NOT vertebroplast* NOT coccydynia
Differential Diagnos* AND low* AND Back Pain NOT joint NOT osteoporos* NOT fract* NOT disc* NOT spondyl* NOT spinal stenosis NOT radic* NOT musc* NOT child* NOT vertebroplast* NOT coccydynia
Prostatitis OR Endometriosis OR Extrauterine pregnancy OR Ovarian carcinoma OR Hematocolpos AND low* AND Back Pain NOT fract* NOT child* NOT vertebroplast* NOT coccydynia
Chronic pelvic inflammatory disease OR Nephrolithiasis OR Pyelonephritis OR Perinephric Abscess OR Renal Ischemia OR Renal Vein Thrombosis OR Kidney cell Carcinoma AND low* AND Back Pain NOT fract* NOT child* NOT vertebroplast* NOT coccydynia
Aortic aneurysm OR Myocardial infarction OR Pulmonary embolism OR Aortic dissection OR Aortic rupture OR Vena cava thrombosis OR Lung Cancer AND low* AND back pain NOT fract* NOT child* NOT vertebroplast* NOT coccydynia
Pancreatitis OR Cholecystitis OR Penetrating ulcer OR Intestinal ischemia OR Thrombosis of intraabdominal organs OR Hepatic vein Thrombosis OR Duodenal ulcer OR Gall stones OR Retroperitoneal fibrosis AND low* AND Back Pain NOT fract* NOT child* NOT vertebroplast* NOT coccydynia
Lymphoma OR Gout OR Ochronosis OR Hypovitaminosis OR Vitamin D Deficiency OR hyperparathyroidism OR Hypercortisolism OR Neuroschistosomiasis AND low* AND Back Pain NOT fract* NOT child* NOT vertebroplast* NOT coccydynia
atypical AND cause* AND low* AND back pain NOT fract* NOT child* NOT vertebroplast* NOT coccydynia
medical condition* AND cause* AND acute back pain NOT child* NOT vertebroplast* NOT coccydynia
cancer* AND cause* AND low* AND back pain NOT fract* NOT child* NOT vertebroplast* NOT coccydynia
infect* AND fever* AND cause* AND low* AND back pain NOT fract* NOT child* NOT vertebroplast* NOT coccydynia
metabol* disease AND cause* AND low* AND back pain NOT fract* NOT child* NOT vertebroplast* NOT coccydynia
genet* disease AND cause* AND low* AND back pain NOT fract* NOT child* NOT vertebroplast* NOT coccydynia
unusual OR uncommon OR rare OR medical condition AND cause* AND lumbar AND radiculopathy NOT fract* NOT child* NOT vertebroplast* NOT coccydynia
**MeSH-Terms Cochrane**
uncommon cause* AND low* AND back pain NOT fract*
unusual cause* AND low* AND back pain NOT fract*
rare cause* AND low* AND back pain NOT fract*
differential diagnos* AND low* AND back pain NOT fract*
medical condition AND cause* AND low* AND back pain NOT fract*
Prostatitis AND low* AND back pain NOT fract*
Endometriosis AND low* AND back pain NOT fract*
Extrauterine pregnancy AND low* AND back pain NOT fract*
Ovarian carcinoma AND low* AND back pain NOT fract*
Hematocolpos AND low* AND back pain NOT fract*
Chronic pelvic inflammatory disease AND low* AND back pain NOT fract*
Nephrolithiasis AND low* AND back pain NOT fract*
Pyelonephritis AND low* AND back pain NOT fract*
Perinephric abscess AND low* AND back pain NOT fract*
Renal ischemia AND low* AND back pain NOT fract*
Renal vein thrombosis AND low* AND back pain NOT fract*
Kidney cell Carcinoma AND low* AND Back Pain NOT fract*
Aortic aneurysm AND low* AND Back Pain NOT frac*
Myocardial infarction AND low* AND Back Pain NOT fract*
Pulmonary embolism AND low* AND Back Pain NOT fract*
Aortic dissection AND low* AND Back Pain NOT fract*
Aortic rupture AND low* AND Back Pain NOT fract*
Vena cava thrombosis AND low* AND Back Pain NOT fract*
Lung Cancer AND low* AND Back Pain NOT fract*
Pancreatitis AND low* AND Back Pain NOT fract*
Cholecystitis AND low* AND Back Pain NOT fract*
Penetrating ulcer AND low* AND Back Pain NOT fract*
Intestinal ischemia AND low* AND Back Pain NOT fract*
Thrombosis of intraabdominal organs AND low* AND Back Pain NOT fract*
Hepatic vein Thrombosis AND low* AND Back Pain NOT fract*
Duodenal ulcer AND low* AND Back Pain NOT fract*
Gall stones AND low* AND Back Pain NOT fract*
Retroperitoneal fibrosis AND low* AND Back Pain NOT fract*
Lymphoma AND low* AND Back Pain NOT fract*
Gout AND low* AND Back Pain NOT fract*
Ochronosis AND low* AND Back Pain NOT fract*
Hypovitaminosis AND low* AND Back Pain NOT fract*
Vitamin D Deficiency AND low* AND Back Pain NOT fract*
Hyperparathyreoidism AND low* AND Back Pain
Hypercortisolism AND low* AND Back Pain
Neuroschistosomiasis AND low* AND Back Pain
atypical AND cause* AND low* AND back pain NOT fract*
medical condition* AND cause* AND acute back pain
cancer* AND cause* AND low* AND back pain NOT fract*
infect* AND fever AND cause* AND low* AND back pain
metabol* disease AND cause* AND low* AND back pain NOT fract*
genet* disease AND cause* AND low* AND back pain NOT fract*
unusual AND cause* AND lumbar AND radiculopathy NOT fract*
uncommon AND cause* AND lumbar AND radiculopathy NOT fract*
rare AND cause* AND lumbar AND radiculopathy NOT fract*
medical condition AND cause* AND lumbar AND radiculopathy NOT fract*

## Appendix 3

**Table 36:** Excluded pathologies

**Category of excluded pathologies**	**Excluded pathologies**
Infection of the spine and paravertebral muscles (red flag pathology)	• inflammatory spondyloarthropathy • axial spondyloarthropathy / spondylarthritis • Bechterew’s disease / ankylosing spondylitis • discitis / septic discitis • septic arthritis (zygapophyseal) • osteomyelitis • lumbar interspinous bursitis • spondylodiscitis • juxtafacet disc infection • myelitis • helminthosis: hydatid, cysticercosis • abscess (spinal, intradural extramedullary, subdural, epidural) • psoas abscess • arachnoiditis (spinal, ossificans) • Potts disease / spinal tuberculosis • Elsberg-syndrome (radiculomyelitis) • meliodosis • neuroschistosomiasis • tuberculosis of liver and other organs • Guillain Barré syndrome • zoster and herpes infection • borreliosis with radiculopathy
Benign and malignant tumour (red flag pathology)	• benign and malignant tumour of the spine and spinal structures including metastases, invasive infiltration and intramedullary tumour • plasmacytoma • Non-Hodgkin-Lymphoma • thymoma • multiple myeloma • lymphangioma • intestine mesenteric low-grade fibromyxoid sarcoma • adenocarcinoma • pheochromocytoma • tumour in other organs: renal, rectal, lung, prostate, ovaries, neuroendocrine, liver, pancreas, lipomatosis, uterus, (intra)peritoneal • pigmented villonodular synovitis
Selected neurological disorders	• bowstring disease (caused by nerve compression lesion) • dorsal root ganglion compression • lumbar dorsal ramus syndrome • foot drop • meralgia paraesthetica • superior cluneal nerve entrapment
Rheumatic inflammatory disease	• rheumatoid arthritis • rheumatoid nodules • axial psoriatic spondyloarthropathy • Behcet’s disease
Specific LBP with anatomical changes of the spine and disc, including malformation of the spine, spinal cord, and meninges	• Modic changes • lumbar lordosis • scoliosis / pseudoscoliosis • Scheuermans disease • lumbar disc herniation / spinal cord herniation / Schmorl node / ruptured anulus fibrosus • degenerative disc disease • internal disc rupture • lumbar facet joint syndrome • Baastrup disease • cysts (discal, lumbar synovial, extradural meningeal, cystic dilatation of ventriculus terminalis, extraforaminal, epidermoid, tarlov, perineural, ependymal, sacral extradural arachnoid cyst, lateral sacral, zygapophyseal, zygapophyseal joint synovial) • malformation (pseudomeningocele, meningocele, myelomeningocele, duplicated filum terminal, tethered cord) • spondylolisthesis / retrolisthesis • spinal stenosis • spondylolysis • pseudarthrosis • Bertolotti syndrome • progressive necrotic myelopathy • ectopic ganglion in cauda equina • dural ectasia
Functional diagnosis	• sacro-iliac-joint syndrome • piriformis syndrome / pyomyositis • fibromyalgia (as a functional physical discomfort) • lumbar spinal instability • lumbago • coccygodynia • zygapophyseal joint pain • quadratus femoris syndrome / muscle rupture • GNE myopathy
Bone disease	• osteoporosis • osteopetrosis • melorheostosis • Morbus Paget • osteonecrosis of the spine • fibrous dysplasia
Complication of interventions	• textiloma • retrievable filter • injection therapies • surgery • drug-treatment complication (e.g., epidural hematoma due to aspirin / anticoagulant) • erythema ab igne • ice-pack induced perniosis • epidural pneumorrhachis • lost intrauterine device
Trauma / injury / fracture (red flag pathology)	• injury • traumatic compartment syndrome • non-traumatic fracture
Pregnancy-related LBP	• complication of treatment / pregnancy / birth / afterbirth
Other	• depression, somatoform disorders • Stiff-Person-Syndrome • thoracic or cervical pain • Whipple’s disease • decompression sickness • histiocytosis (Rosai-Dorfman, Erdheim-Chester) • CARASIL / CADASIL • ligamentum flavum haematoma • pelvic congestion syndrome • animal model • children and adolescents

## Abbreviations

25-OH: 25-Hydroxy
AAA: Abdominal aortic aneurysm
AAD: Acute aortic dissection
AAS: Acute aortic syndrome
ACF: Aorto-caval fistula
AD: Aortic dissection
ADF: Aorto-duodenal fistula
AIP: Autoimmune pancreatitis
ANA: Antinuclear antibody
AV: Arterio-venous
AVM: Arterio-venous malformation
BMI: Body mass index
BP: Blood pressure
CADASIL: Cerebral autosomal dominant arteriopathy with subcortical infarcts and leukencephalopathy
CARASIL: Cerebral autosomal recessive arteriopathy with subcortical infarcts and leukencephalopathy
CCS: Case-control study
CE-CT: Contrast enhanced computed tomography
CHF: Congestive heart failure
CHS: Cohort study
CK: Creatine kinase
COPD: Chronic obstructive pulmonary disease
CR: Case report
CRP: C-reactive protein
CS: Case series
CSS: Cross-sectional study
CT: Computed tomography
CTA: Computed tomography angiography
DDx: Differential diagnosis
DSA: Digital subtraction angiography
DVT: Deep venous thrombosis
e.g.: Exempli gratia
ECG: Electrocardiogram
ED: Emergency department
EM: Patients with endometriosis
ERC: Endoscopic retrograde cholangiography
ERCP: Endoscopic retrograde cholangiopancreatography
ESKD: End stage kidney disease
ESR: Erythrocyte sedimentation rate
f: Female
fDDx: Part of first differential diagnosis
FDG: Fluorodeoxyglucose
FDG-PET: Fluorodeoxyglucose-positron emission tomography
Ga-67: Gallium-67
GI: Gastrointestinal
GNE: Glucosamine (UDP-N-acetyl)-2-epimerase/N-acetylmannosamine kinase gene
GP: General practitioner
gynD: Gynaecologic diseases
h/o: History of
HLA-DR: Human leukocyte antigen – DR isotype
IAAA: Inflammatory abdominal aortic aneurysm
IgG4: Immunoglobulin G4
IL-6: Interleukin 6
IMH: Intramural haematoma
IRAD: International Registry of Aortic Dissection
IRF: Idiopathic retroperitoneal fibrosis
IVC: Inferior vena cava
IVU: Intravenous urogram
LBP: Low back pain
LEL: Lumbosacral epidural lipomatosis
LOC: Loss of consciousness
LP: Lumbar puncture
m: Male
marf: Marfan
MeSH: Medical Subject Headings
MR: Magnetic resonance
MRA: Magnetic resonance angiography
MRCP: Magnetic resonance cholangiopancreatography
MRI: Magnetic resonance imaging
msCT: multi-slice computed tomography
nfDDx: Not part of first differential diagnosis
nmarf: Non-Marfan
NR: Narrative review
NRS: Numeric rating scale
NSAID: Non-steroidal anti-inflammatory drugs
NSAR: Non-steroidal anti-rheumatic drug
normPel: Normal pelvis
PAU: Penetrating aortic ulcer
PET: Positron emission tomography
pf: Painful
pl: Painless
PTGBA: Percutaneous transhepatic gallbladder aspiration
PTGBD: Percutaneous transhepatic gallbladder drainage
RCT: Randomised controlled trial
RPF: Retroperitoneal fibrosis, retroperitoneal fibrosis
SAH: Subarachnoid haemorrhage
Sino-RAD: Registry of Aortic Dissection in China
SLE: Systemic lupus erythematosus
SPECT-CT: Single photon emission tomography - computed tomography
SR: Systematic review
STEMI: ST-elevation myocardial infarction
Tc-99m MAG3-scintigraphy: Technetium-99m mercaptoacetyltriglycine scintigraphy
TCC: Transitional cell carcinoma
TEE: Transoesophageal echocardiogram
TLOC: Transient loss of consciousness
TNF: Tumor necrosis factor
TTE: Transthoracic echocardiogram
UDT: Urine dipstick test
US: Ultrasonography
UTI: Urinary tract infection
VAS: Visual analog scale
vit: Vitamin
w: With
wo: Without
y: Years
